# Spatial tumour gene signature discriminates neoplastic from non-neoplastic compartments in colon cancer: unravelling predictive biomarkers for relapse

**DOI:** 10.1186/s12967-023-04384-0

**Published:** 2023-08-05

**Authors:** Katja Sallinger, Michael Gruber, Christin-Therese Müller, Lilli Bonstingl, Elisabeth Pritz, Karin Pankratz, Armin Gerger, Maria Anna Smolle, Ariane Aigelsreiter, Olga Surova, Jessica Svedlund, Mats Nilsson, Thomas Kroneis, Amin El-Heliebi

**Affiliations:** 1 Division of Cell Biology, Histology and Embryology, Gottfried Schatz Research Centre, Medical University of Graz, Graz, Austria; 2 Center for Biomarker Research in Medicine (CBmed), Graz, Austria; 3 Division of Oncology, Department of Internal Medicine, Medical University of Graz, Graz, Austria; 4 Department of Orthopaedics and Trauma, Medical University of Graz, Graz, Austria; 5 Diagnostic and Research Institute of Pathology, Medical University of Graz, Graz, Austria; 6 Science for Life Laboratory, Department of Biochemistry and Biophysics, Stockholm University, 17165 Solna, Sweden; 7 10x Genomics, Life City, Solnavägen 3H, 113 63 Stockholm, Sweden; 8 Biotechmed, Graz, Austria

**Keywords:** In situ sequencing, Spatial transcriptomics, colon cancer, Predictive biomarker, Tumour compartment, Tumour gene signature

## Abstract

**Background:**

Opting for or against the administration of adjuvant chemotherapy in therapeutic management of stage II colon cancer remains challenging. Several studies report few survival benefits for patients treated with adjuvant therapy and additionally revealing potential side effects of overtreatment, including unnecessary exposure to chemotherapy-induced toxicities and reduced quality of life. Predictive biomarkers are urgently needed. We, therefore, hypothesise that the spatial tissue composition of relapsed and non-relapsed colon cancer stage II patients reveals relevant biomarkers.

**Methods:**

The spatial tissue composition of stage II colon cancer patients was examined by a novel spatial transcriptomics technology with sub-cellular resolution, namely in situ sequencing. A panel of 176 genes investigating specific cancer-associated processes such as apoptosis, proliferation, angiogenesis, stemness, oxidative stress, hypoxia, invasion and components of the tumour microenvironment was designed to examine differentially expressed genes in tissue of relapsed versus non-relapsed patients. Therefore, FFPE slides of 10 colon cancer stage II patients either classified as relapsed (5 patients) or non-relapsed (5 patients) were in situ sequenced and computationally analysed.

**Results:**

We identified a tumour gene signature that enables the subclassification of tissue into neoplastic and non-neoplastic compartments based on spatial expression patterns obtained through in situ sequencing. We developed a computational tool called Genes-To-Count (GTC), which automates the quantification of in situ signals, accurately mapping their position onto the spatial tissue map and automatically identifies neoplastic and non-neoplastic tissue compartments. The GTC tool was used to quantify gene expression of biological processes upregulated within the neoplastic tissue in comparison to non-neoplastic tissue and within relapsed versus non-relapsed stage II colon patients. Three differentially expressed genes (*FGFR2*, *MMP11* and *OTOP2*) in the neoplastic tissue compartments of relapsed patients in comparison to non-relapsed patients were identified predicting recurrence in stage II colon cancer.

**Conclusions:**

In depth spatial in situ sequencing showed potential to provide a deeper understanding of the underlying mechanisms involved in the recurrence of disease and revealed novel potential predictive biomarkers for disease relapse in colon cancer stage II patients. Our open-access GTC-tool allowed us to accurately capture the tumour compartment and quantify spatial gene expression in colon cancer tissue.

**Supplementary Information:**

The online version contains supplementary material available at 10.1186/s12967-023-04384-0.

## Introduction

Colorectal cancer is the third most commonly diagnosed cancer, and the second leading cause of cancer associated mortality worldwide [1]. In the EU alone, the incidence of colorectal cancer has been steadily increasing by a factor of 0.4% each year which is associated to life style effects [2]. The 5-year overall survival is strongly associated with stage at diagnosis, estimated at 93%, 88%, 81% and 32% for stages I, II, III and IV, respectively [3]. Therapeutic management has improved significantly over the last decade, including advances in screening, (neo) adjuvant treatment, targeted- and immune checkpoint therapies [4]. Surgery remains the primary treatment approach for nonmetastatic colon cancer, with histopathological staging guiding the decision to administer adjuvant chemotherapy for a duration of up to 6 month. While the efficacy of adjuvant chemotherapy has been firmly established for patients diagnoses with stage III colon cancer, its utility in the context of stage II disease remains a topic of ongoing debate and discussion [5]. The European society for medical oncology (ESMO) released clinical practice guidelines for treating stage II colon cancer patients [6]. In general, adjuvant therapy options need to be discussed with the patient, addressing the expected benefit from chemotherapy versus the risk of complications. The risk of relapse after colon cancer resection can be estimated by assessing the tumour, node, metastasis (TNM) stage, mismatch repair (MMR)/microsatellite instability (MSI) status, and number of lymph nodes sampled [6]. In a low-risk scenario, the colon cancer is resected and the patient does not receive adjuvant chemotherapy. In patients with high-risk features who are “fit” according to the Carlson Comorbidity Index shall be treated with adjuvant chemotherapy [6]. These high-risk features include clinic-pathological parameters such as T4 tumours, perineural or lymphovascular invasion, poorly or undifferentiated tumour grade, intestinal obstruction or tumour perforation [6, 7]. However, these high-risk features are unreliable in predicting beneficial effects of adjuvant treatment [7]. Vice versa, low-risk patients may develop tumour recurrence quite early after surgery [7]. The overall survival benefit in stage II colorectal cancer (CRC) trials for patients treated with chemotherapy indicates no or just a little improvement (below 5%) [8]. Additionally, chemotherapy treatment is associated with side effects such as pain, insomnia, vomiting, diarrhoea, increased amounts of white blood cells and potential toxicities, thus adjuvant therapy for all patients with diagnosed stage II colon cancer would be an overtreatment with no or little benefit in outcome but a potential risk of reduced quality of life [9]. Therefore, more precise biomarkers indicative for early recurrence in stage II colon cancer are needed [10]. Various biomarker panels have been examined in vivo for the purpose of recurrence prediction. Yamanaka et al. conducted a study in which they utilized a 12-gene recurrence assay to identify patients at high risk of tumor recurrence. These individuals were recommended to receive additional chemotherapy treatment [11]. Similarly, Kopetz et al. developed an 18-gene expression-based classifier called ColoPrint. This classifier serves the purpose of identifying patients with a high risk of disease recurrence, thus enabling the selection of individuals who would benefit from adjuvant chemotherapy [12]. Furthermore, other studies conducted on CRC tissue and colon cancer cell lines have provided additional evidence supporting the potential of small nucleolar RNAs as predictive biomarkers for high-risk recurrence and poor prognosis in patients with CRC stage II [13]. In a separate development, Pages et al. developed a tool called the Immunoscore, which relies on immunohistochemistry staining of CD3+ and CD8+ cells. Through this innovative approach, the study encompassed 2681 patients who were classified into low-, intermediate-, and high-risk groups. Importantly, patients with a high Immunoscore demonstrated the lowest risk of disease recurrence [14].

Although aforementioned approaches are promising, no method was able to reach the clinical routine. Each approach is either based on small antibody combinations (CD3+CD8 immunohistochemistry), bulk tissue analysis using reverse transcription-polymerase chain reaction (RT-PCR) or array based approaches [10, 15]. Bulk analyses, however, only inform on the average sub-clonal composition with strong bias towards the largest clones present [16]. Similarly, the spatial histological architecture is lost in bulk RNA or DNA analysis due to tissue lysis [17]. As such, important biological processes, i.e.: proliferation or metabolic dysregulations in cancer are only indirectly measurable without providing insights into their spatial interactions [18]. Understanding the spatial expression patterns of neoplastic tumour tissue and their surrounding microenvironment on a subcellular level, however, can improve the knowledge of disease recurrence [19].

Driven by the advent of single cell RNA sequencing (scRNAseq), our knowledge of the basic molecular mechanisms in colon cancer has increased substantially within the last years [20]. Using scRNAseq the transcriptomic diversity of different cell types has been revealed in high detail whereby the major drawback persists to be the loss of spatial information due to the dissociation of tissue structure. To overcome this issue spatial transcriptomics approaches have been developed such as hybridisation-based in situ sequencing (HybISS) and direct RNA hybridisation-based in situ sequencing (dRNA-HybISS) allowing for highly multiplexed spatial mapping of transcripts within tissues [21].

Here, we hypothesise that the spatial histological expression patterns of relapsed and non-relapsed colon cancer stage II patients differ. Therefore, we aimed to investigate the spatial tissue composition in yet unprecedented resolution by dRNA-HybISS down to single-cells and -molecules beyond current spatial transcriptomics approaches in colon cancer stage II patients. First, we designed a panel of 176 genes to examine the expression of various important biological processes in colon cancer including angiogenesis, apoptosis, proliferation, stemness, hypoxia, oxidative stress, invasion, and energy metabolism as well as markers for components of the microenvironment including cancer associated fibroblasts. Based on the expression pattern of the targeted ISS genes, in particular a tumour gene signature, tissue compartments can be automatically generated to subclassify the investigated tissue into neoplastic and non-neoplastic compartments. By using these gene expression-based tissue compartments we are able to quantify gene expression related to biological processes shown to be upregulated within the neoplastic tissue in comparison to non-neoplastic tissue. Second, we statistically evaluated which spatially differential expressed genes are predictive for tumour recurrence.

Summarized, we identified a spatial tumour gene signature and developed a computational tool to classify tissue into neoplastic and non-neoplastic tissue by in situ sequencing informed expression. We thereby identified *FGFR2*, *MMP11* and *OTOP2* as three differentially expressed genes in the neoplastic tissue predicting tumour recurrence in stage II colon cancer.

## Materials and methods

### Study design–patient cohort

For this retrospective study, 10 patients were selected with diagnosed stage II colon cancer from the Biobank Graz. Each patient was observed for at least 3 years after tumour resection and their final tumour recurrence status labelled as either relapsed (5 patients, 50%) or non-relapsed (5 patients, 50%). To ensure a homogeneous patient population, we selected patients who shared the same diagnosis of stage II colon cancer and had undergone surgical resection as the primary treatment modality. Importantly, none of the patients in the study received any additional adjuvant chemotherapy following the surgery. This standardized approach allowed us to focus specifically on the role of the spatial tumor composition for relapse prediction, without confounding factors related to postoperative treatments. Tumour tissue was formalin fixed and paraffin embedded (FFPE). Neoplastic and non-neoplastic colonic tissue was present in each tissue section (Additional file 1: Table S1).

### Ethic approval

The study protocol was approved by the Ethics Committee of the Medical University of Graz (29-187 ex 16/17) following the declaration of Helsinki and good clinical practice, and written informed consent was obtained by all patients.

### Panel design

A panel of padlocks was designed to target 176 transcripts with the intent of investigating different biological processes within the tumour and its microenvironment. The panel includes genes involved in angiogenesis, apoptosis, autophagy, necrosis, proliferation, oxidative stress, hypoxia, stemness, invasion, epithelial–mesenchymal transition (EMT), energy metabolism as well as different epithelial cells, tumour associated stromal cells and immune cells (Additional file 1: Table S2). The exact target sequences and padlock probes design is propriety information by Cartana (Stockholm, Sweden, now part of 10x Genomics, California, USA) and are not known by the authors.

### Tissue preparation and ISS library preparation

For application of the in situ sequencing method, 5 μm FFPE tissue sections were processed according to the manufacturer’s protocols and kits (High Sensitivity library preparation kit, Cartana). In short, sections were baked at 60 °C for 1 h, deparaffinised in xylene or Histolab Clear (Sanova Pharma, Vienna, Austria), rehydrated and permeabilised in a steamer using citrate buffer of pH6 for 45 min. The sections were then dehydrated in an ethanol series and air-dried followed by the attachment of hybridization chambers (Secure Seal, Grace Biolabs, Oregon, USA). All hybridisation steps were performed in RNAse free humidity chambers. Padlock probes were then directly hybridised to the RNA at 37 °C overnight, followed by ligation at 37 °C for 2 h. After the ligation process, a circular oligonucleotide was formed and amplified overnight at 30 °C in a rolling circle amplification (RCA) reaction, resulting in RCA products (RCP).

### Sequencing and stripping

Adapter probes (Sequencing kit, Cartana) were hybridised at 37 °C for 1 h, followed by a washing step with washing buffer 2 (WB2). Afterwards the sequencing probes were hybridised at 37 °C for 30 min. The sections were washed with WB2, mounted with SlowFade Gold Antifade Mountant (Thermo Fisher Scientific, Massachusetts, USA) and imaged. The procedure for every sequencing cycle was as follows: after each sequencing cycle, adapter- and sequencing-probes were stripped off by adding three times 100% formamide to each slide for 1 min at room temperature. This step was followed by a washing step with WB2. The ISS cycles were repeated for a total of 5 times, with 5 different adapter probe pools and imaged in 5 channels (DAPI, FITC, Cy3, Cy5, Cy7). After imaging of the last sequencing cycle, the probes were stripped off one more time and samples were imaged to obtain the autofluorescent background of the tissue in each channel for background correction.

### Imaging

Imaging was performed using a digital slide scanner (Slideview VS200, Olympus, Tokio, Japan) using a LED source (Excelitas Technologies, X-Cite Xylis, Mississauga, Canada). Fluorescence filter cubes and wheels were equipped with a pentafilter (AHF, excitations: 352–404 nm, 460–488 nm, 542–566 nm, 626–644 nm, 721–749 nm; emissions 416–452 nm, 500–530 nm, 579–611 nm, 665–705 nm, 767–849 nm). The images were obtained with a sCMOS camera (2304 × 2304, ORCA-Fusion C14440-20UP, 16 bit, Hamamatsu, Japan), and Olympus universal-plan super apochromat 40× (0.95 NA/air, Olympus). For each slide and cycle imaging in DAPI, FITC, Cy3, Cy5 and Cy7 was performed. Extended focus imaging (EFI) was used to automatically discard unfocused z-stack images, resulting in bright and focused in situ signals.

### Image analysis

Imaging data was analysed with a custom pipeline provided by Cartana (part of 10x Genomics) and published pipelines found in the repository (https://github.com/Moldia/HybrISS) handling image processing and gene calling [22]. All code was written in MATLAB. Additionally, a CellProfiler (v.2.1.1) pipeline with the ImageJ plugins MultiStackReg, StackReg and TurboReg was used to perform a second, more exact alignment between the cycles [23]. Used pipelines can be found in the repository (https://github.com/spatialhisto/GTC). Images from all sequencing cycles were exported into .tiff- format and aligned through the DAPI channel of the first sequencing cycle with the channels of each sequencing cycle. Then, images were split into multiple smaller images to allow analysis in CellProfiler.

As each fluorescent colour had different intensity values for RCP signals in their respective colours, we normalized the intensity values to 10,000 and computed the corresponding multiplication factor. E.g. the median intensity of RCP signal in Cy5 was 5000 and, therefore, was multiplied by two to reach 10,000. This multiplication factor was calculated for each fluorescent colour and then used to normalise the median intensity of all RCP signals. The received multiplication factor for each channel was integrated in the CellProfiler pipeline and the background of each channel was subtracted from each sequencing cycle to reduce the autofluorescence of the tissue. A pseudo-general stain was created by combining the 4 readout detection probe channels of the first sequencing cycle into one merged image. Additionally, a pseudo-anchor for each sequencing cycle was generated to perform a second alignment to the pseudo-general stain. The RCPs of the pseudo-general stain were detected to obtain the x- and y-coordinates of the ISS genes. Based on these positions, the fluorescence intensities in each of the 4 channels (FITC, Cy3, Cy5 and Cy7) were measured. This procedure was performed for all sequencing cycles to derive the measured intensities. The highest intensity value in each sequencing cycle was then assigned as real signal and further used for decoding with MATLAB [22]. For the verification of the signals, the selected transcripts were plotted on a DAPI-stained image [22].

### Quality assessment of FFPE tissue samples

All tissue samples were processed according to established SOPs for routine lab procedures of the Diagnostic and Research Institute of Pathology, Medical University of Graz (Austria). Quality of RNA was assessed by quantifying the expected decoded transcript of the in situ sequencing raw data which is included in the described MATLAB pipeline and is depicted in Additional file 1: Table S3.

### Tissue compartment building by morphology and virtual H&E

**Fig. 1:**
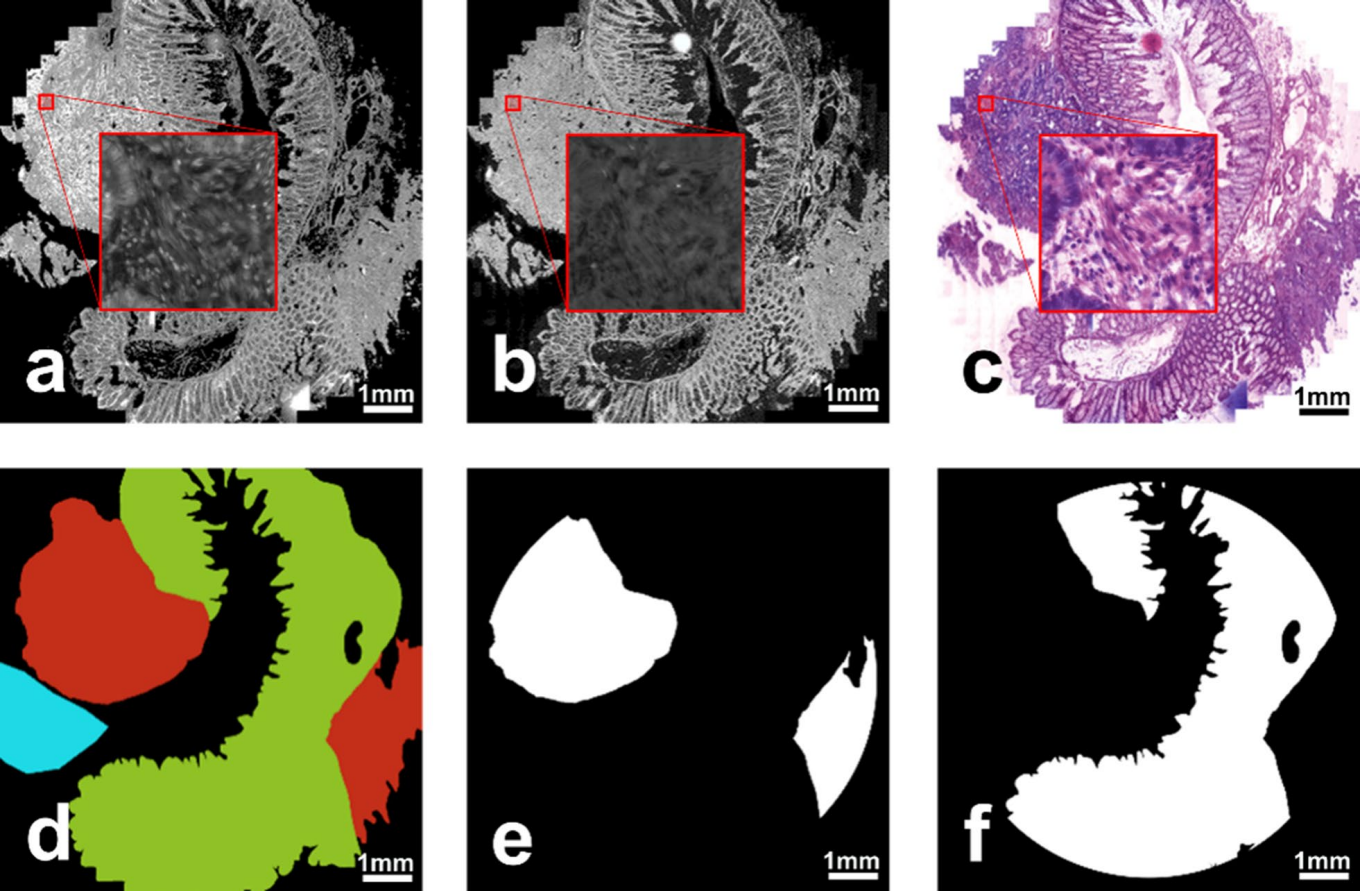
Generation of the virtually stained H&E image and compartment building. **a** DAPI-stained image, **b** FITC-stained image used for calculating of **c** the virtually stained H&E image of the tissue sample. **d** The tissue areas in the tissue sections as classified by a pathologist: red–neoplastic tissue, green–non-neoplastic tissue. The blue area marks a region that was excluded from the analysis due to high autofluorescence or lost tissue during hybridisation. The derived representative binary tissue compartment (TC) **e** for the neoplastic and **f** for the non-neoplastic tissue

To combine the advantage of ISS and H&E morphology on the exact same tissue section, we created computationally (virtually) stained H&E images of the ISS hybridised tissue sections based on DAPI and FITC images, as described by Giacomelli et al. [24] (Fig. 1 and Additional file 1: Method S1). In short, by virtually colouring the DAPI image with a colour representing the haematoxylin staining (blue) and the FITC image with a colour representing the eosin staining (red), an H&E image was obtained similar to a stained H&E image. This was possible as the DAPI channel stained the cell nuclei, while the FITC autofluorescence provided the cell outlines and fibers of the tissue. The virtually stained H&E images of the patient tissue samples were subsequently evaluated by a colon cancer specialised pathologist who assigned tissue areas into neoplastic and non-neoplastic areas (“morphology-based” approach). After morphology-based classification by the pathologist, the two different tissue compartments, i.e.: neoplastic and non-neoplastic area per tissue sample, were outlines and one compartment was converted into black, the other into white images (= binary image) as described in Additional file 1: Method S2. By this approach, each detected gene transcript could be assigned to either the neoplastic area, or non-neoplastic area.

### Tissue compartment building by gene expression

**Fig. 2:**
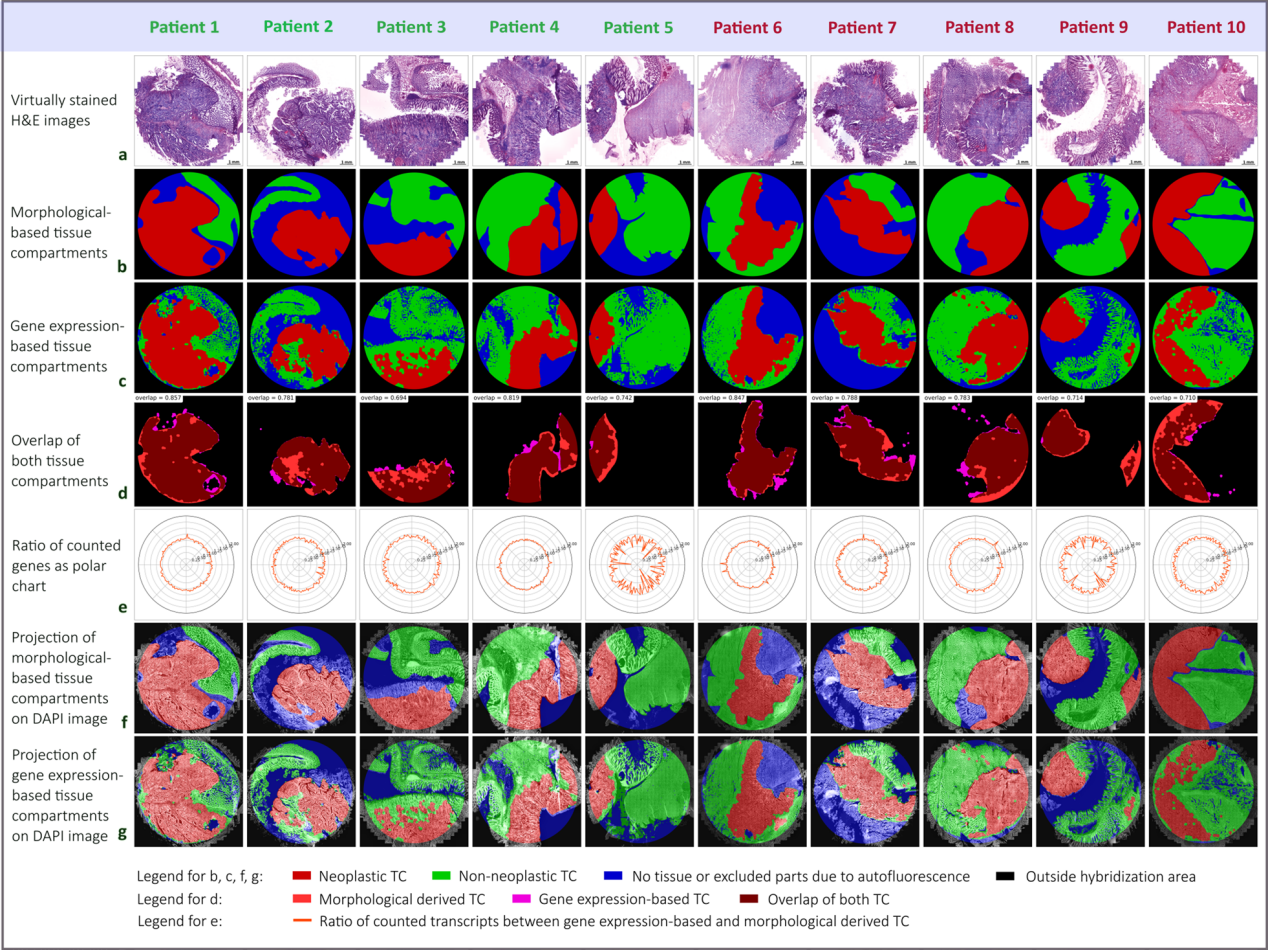
Generation of expression-based tissue compartments and overlap with morphological tissue compartments. **a** The virtually stained H&E images of the samples from non-relapsed (patient 1–5) and relapsed patients (patient 6–10). **b** Tissue classified into neoplastic and non-neoplastic tissue compartment by a pathology expert based on morphological characteristics. **c** Gene expression-based neoplastic and non-neoplastic tissue compartment by using the in situ sequencing tumour gene signature (*EREG*, *MET*, *BIK*, *CD44*, *ITGAV*, *MYBL2*, *CCND1* and *S100A4*). **d** Overlap of the morphological- and the gene expression-based tissue compartment for neoplastic tissue. The mean overlap-value for the tumour gene signature is 0.77. **e** Ratios of the counted gene per cell value between the gene expression-based and the morphological- based neoplastic tissue compartment depicted as polar chart. Thereby, each data point shows the ratio for a certain in situ sequencing gene. **f** Projection of morphological obtained tissue compartment on the DAPI images. **g** Projection of gene expression-based tissue compartment on the DAPI images. Size bar is the same for all images

Most of the via ISS analysis detected transcripts were expressed in neoplastic- and non-neoplastic tissue compartment. However, specific genes showed clear overexpression in neoplastic vs. non-neoplastic tissue compartments in all analysed tissue samples. Based on this observation, we evaluated if a set of genes as such could be used to automatically classify tissue into neoplastic and non-neoplastic tissue compartments without future need for histopathological information. In doing so, in situ signals of the respective genes were computationally represented as dots of a certain size and computationally superimposed to form connected areas, as shown in Fig. 2. The detailed description of this approach can be found in Additional file 1: Method S3 and S4. In short, the dot like signals were expanded by 50–180 pixels, thereby merging and forming larger, connected areas (see Additional file 1: Table S4). In order to remove small gaps in connected tissue compartments, the python library openCV [25] was used. A threshold technique was subsequently applied to convert this superimposition into a binary neoplastic tissue compartment. Next, the overlap between the “morphological-based” and “gene expression-based” binary neoplastic tissue compartment was calculated for each sample. The sample overlaps were, further, combined via geometric mean to a mean overlap-value that was used to rate the set. The mean overlap-values were calculated for alternating compositions of genes. Finally, the set of ISS genes that achieved the highest mean overlap-value was selected and used for statistical testing (see Additional file 1: Method S4). The binary non-neoplastic tissue compartment of a patient sample was obtained by excluding the neoplastic one from the entire tissue compartment where latter was derived by superimposition of the dot representations of all detected ISS genes and all cell nuclei.

### Gene counting and statistical testing

We developed a script to create the tissue compartments and quantify the gene counts, namely genes-to-count (GTC-Tool) available at the repository (https://github.com/spatialhisto/GTC). To identify significances in the distribution of genes in the binary neoplastic and non-neoplastic tissue compartments, the number of genes was counted within both compartments. To take differences in sizes and cell numbers of the tissue compartments into account, counts were normalised to detected cell nuclei (see Additional file 1: Method S5).

Some areas had to be excluded from analysis due to tissue damage acquired during the sequencing procedure, high autofluorescence or bad alignment (see Additional file 1: Method S6). A two-tailed paired t-test was used for the statistical testing of differences of the gene per cell values in the neoplastic and the non-neoplastic tissue compartment. A two-tailed independent t-test was applied for statistical testing of the gene per cell value in the neoplastic tissue compartment in both the relapsed and the non-relapsed patient distributions. The statistical testing was done with a significance level α = 0.05 for the morphological-based and gene expression-based tissue compartment (see Additional file 1: Method S7).

## Results

### Colon tissue can automatically be classified by dRNA-HybISS based gene expression data into neoplastic and non-neoplastic compartments

The in situ sequencing analysis was performed on tissue samples containing neoplastic and non-neoplastic tissue including stroma and/or healthy colonic epithelium. Based on histopathological expertise we developed a gene expression-based tool called genes-to-count (GTC-tool) to identify neoplastic or non-neoplastic compartments. Thereby, a set of, in neoplastic tissue highly expressed, signature genes served as a template for defining a neoplastic tumour compartment. To do so, virtually stained H&E images of each tumour (Fig. 2a) were annotated by a board-certified, colon cancer specialized pathologist to classify neoplastic and non-neoplastic tissue compartments based on the morphology of the tissues (Fig. 2b). The gene expression-based tissue compartments were then generated based on specific expression patterns of an 8-gene set, which we refer to as tumour gene signature, containing the genes *EREG*, *MET*, *BIK*, *CD44*, *ITGAV*, *MYBL2*, *CCND1* and *S100A4*. This tumour gene signature achieved the highest mean overlap of 77% for neoplastic tissue compartments between morphological- and expression-based tissue compartments (Fig. 2c). The remaining tissue was defined as non-neoplastic tissue. Thus, the GTC-tool integrated the spatial coordinates of each decoded transcript and nucleus into its tissue compartment and automatically quantified RNA transcripts in the respective compartment. However, small areas of some patient samples, i.e. of patients 2, 6, 7 and 9 could not be used for analysis due to tissue loss/damage during the sequencing procedure or high autofluorescence and were, therefore, excluded from analysis, as shown in Fig. 2a.

The overlap of the morphological-based and gene expression-based neoplastic tissue compartments is shown in Fig. 2d for each patient sample (patient 1 = 85.7%, patient 2 = 78.1%, patient 3 = 69.4%, patient 4 = 81.9%, patient 5 = 74.2%, patient 6 = 84.7%, patient 7 = 78.8%, patient 8 = 78.3%, patient 9 = 71.4% and patient 10 = 71.0%).

The ratio of the morphological-based and gene expression-based neoplastic tissue compartment gene per cell counts are shown as polar chart in Fig. 2e. As can be seen therein, only a minor variation occurred between both neoplastic tissue compartments. For a better visualization regarding the localization of the compartments within the tissue architecture, DAPI-images were superimposed with the morphological-based (Fig. 2f) and the expression-based tissue compartment (Fig. 2g).

#### Spatially differential gene expression in neoplastic vs. non-neoplastic tissues

**Fig. 3:**
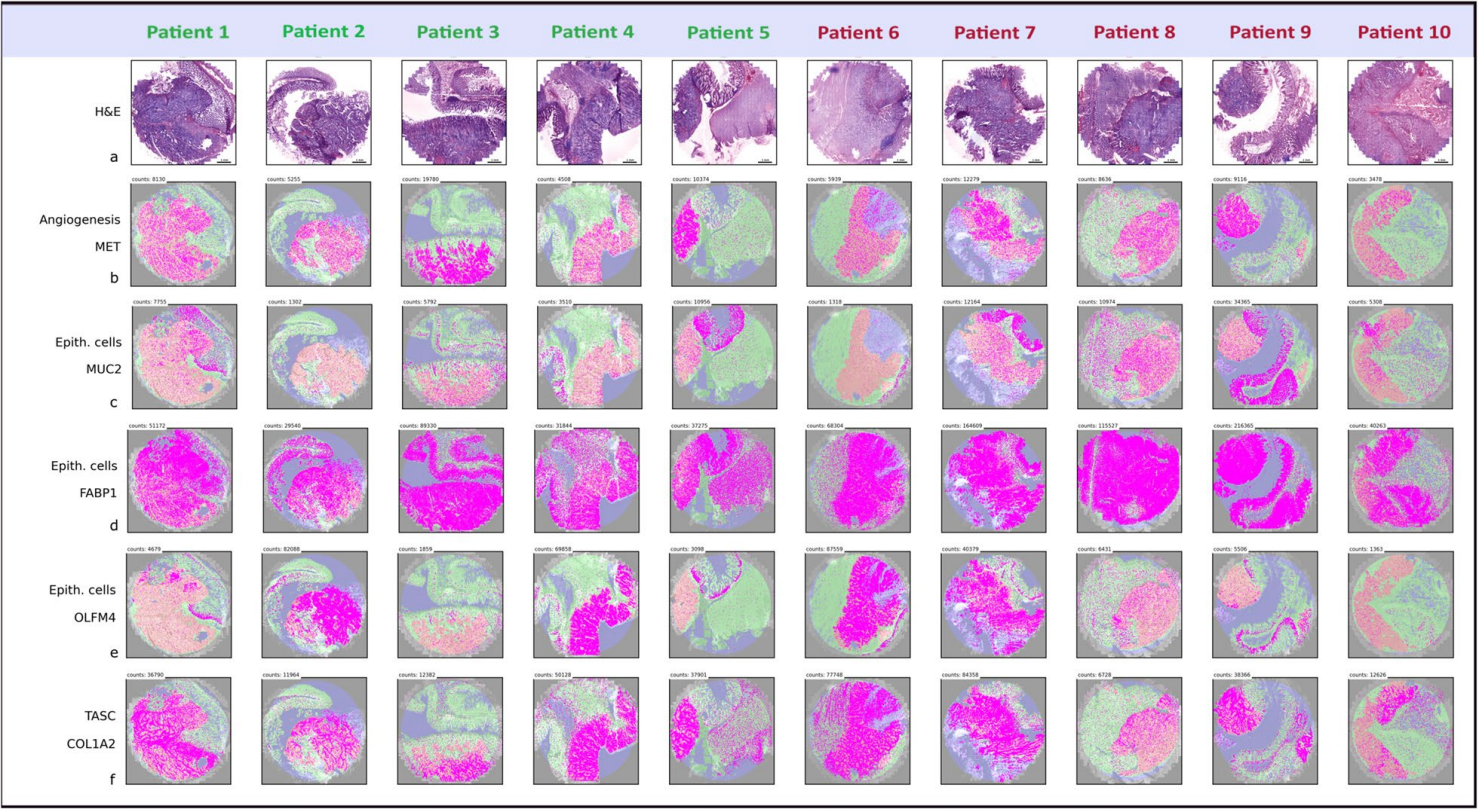
Examples of spatial distributions of 5 out of 176 genes in neoplastic and non-neoplastic tissue. **a** The virtually stained H&E images of the samples from non-relapsed (patient 1–5) and relapsed patients (patient 6–10). **b** Expression and the spatial distribution of *MET*, a gene of the tumour gene signature that was used for the creation of the neoplastic tissue compartment. **c** Exemplified expression and the spatial distribution of *MUC2*, a gene expressed in non-neoplastic epithelial- and cancer cells. **d** Exemplified expression and the spatial distribution *FABP1*, a high expressed gene in colonic tissue. **e** Expression of OLFM4, a gene associated to inflamed colonic epithelium and antiapoptotic features. **f** Expression of COL1A, a gene relevant in forming collagen and found in most connective tissues. Total counts of each transcript are depicted in each image and size bar is the same for all images

The expression level of each transcript was examined by comparing its counts per cell in the neoplastic vs. the non-neoplastic tissue compartments for 10 colon cancer patient samples (Fig. 3 and Additional file 1: Fig. S13).

**Fig. 4:**
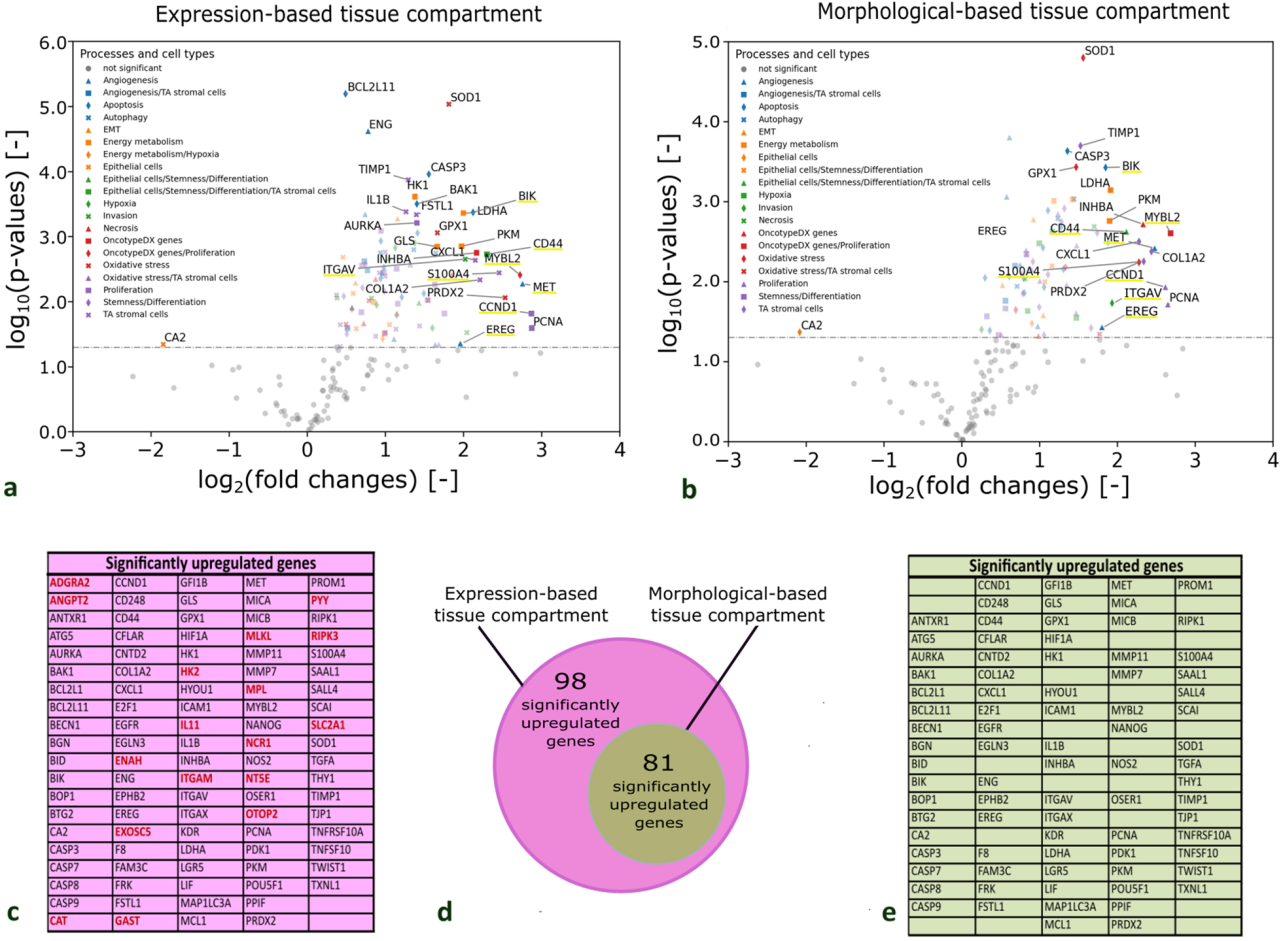
Significantly upregulated genes in neoplastic vs. non-neoplastic tissues compartments (N = 10). **a**, **b** Volcano plot of upregulated genes in the expression-based tissue compartment, and morphological-based tissue compartment. Genes which show a high significance and/or high fold change between the neoplastic and non-neoplastic tissue compartments are labelled by name. Genes belonging to different biological processes are marked with different symbols in different colours to achieve an overview of relevant processes upregulated in neoplastic tissue compartments. Each dot represents an individual gene, a two-sided paired t-test is used for statistical testing with a significance level α = 0.05 (horizontal line). **c** List of all significantly upregulated genes in the expression-based tissue compartment. Red labelled genes were only found significantly differential expressed in the expression-based tissue compartment. Black labelled genes are concordant between expression- and morphological-based tissue compartments. **d** Diagram of the amount of genes upregulated in the expression-based and the morphological tissue compartment. **e** List of all significantly upregulated genes in the morphological tissue compartment. TA stromal cells = tumour associated stromal cells, EMT = epithelial–mesenchymal transition. The 8 identified genes for the tumor gene signature are highlighted in yellow

Therefore, volcano plots with a significance level α = 0.05 were generated to define significantly upregulated genes associated with different biological processes (Fig. 4). 98 significantly upregulated genes were identified in the expression-based tissue compartment (Fig. 4c, d), whereas 81 genes were significantly upregulated in the morphological-based tissue compartment (Fig. 4d, e). All 81 upregulated genes identified by the morphological-based approach were also identified in the expression-based approach.

The morphological neoplastic tissue compartment (Fig. 4a) included: apoptosis related (*CASP3*, *BIK*), proliferation related (*CCND1*, *PCNA*, *MYBL2*), tumour associated stromal genes (*TIMP1*, *CXCL1*,* COL1A2*, *S100A4*, *CD44*), energy metabolism markers (*LDHA*, *PKM*), oxidative stress (*SOD1*, *GPX1*, *PRDX2*), stemness- (*CD44*), angiogenesis- (*MET*) associated genes, and Oncotype DX genes (*INHBA, MYBL2*).

In the expression-based neoplastic tissue compartment (Fig. 4b) the following genes displayed a highly-significant increase in expression and/or a high fold change: apoptosis related (*BCL2L11*, *ENG*, *CASP3*, *BAK*, *BIK*), proliferation related (*AURKA*, *CCND1*, *PCNA*, *MYBL2*) tumour associated stromal genes (*IL1B*, *FSTL1*, *TIMP1*, *CXCL1*, *COL1A2*, *S100A4*, *CD44*), energy metabolism markers (*HK1*, *GLS*, *LDHA*, *PKM*) oxidative stress (*SOD1*, *GPX1*, *PRDX2*), stemness- (*CD44)*, invasion- (*ITGAV*) angiogenesis- (*ENG, MET*) associated genes and Oncotype DX genes (*INHBA, MYBL2*).

#### Upregulated genes in the neoplastic tissue compartment of relapsed patients vs. non-relapsed patients

**Fig. 5:**
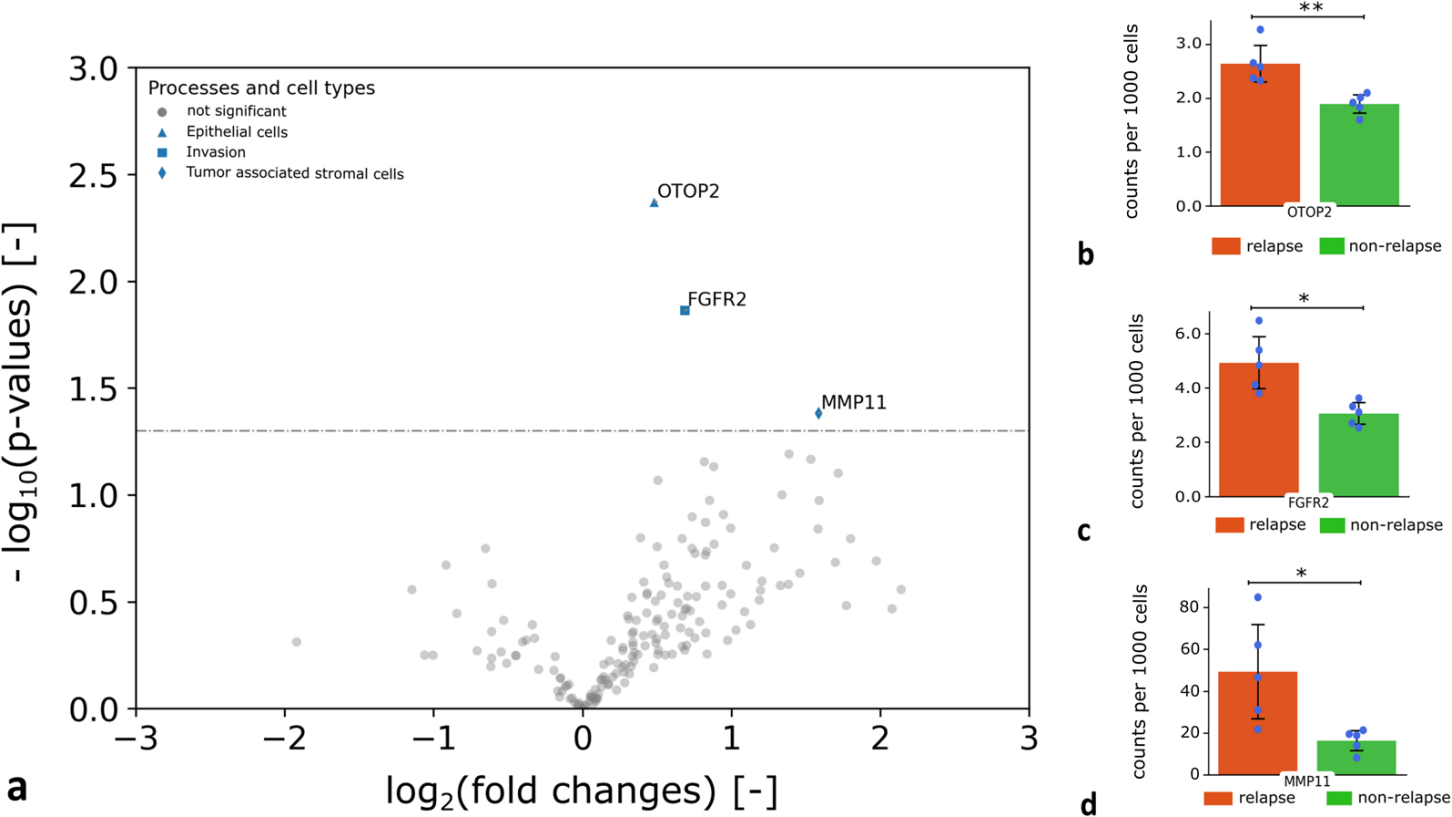
Upregulated genes in neoplastic tissue compartments in relapsed patients in comparison to non-relapsed patients (N = 10). **a** Volcano plot with a significance level α = 0.05 of significantly upregulated genes in the neoplastic tissues compartment of relapsed patients in comparison to non-relapsed patients. **b**–**d** The expression level of *OTOP2*, *FGFR* and *MMP11* in relapsed patients (orange) indicated a significant increase in comparison to non-relapsed patients (green). Significant differences (*p < 0.05 and **p < 0.005) were highlighted with bars and asterisks

A two-sided independent t-test with a significance level α = 0.05 was performed to investigate the differences in gene expression in the neoplastic tissue compartment of 5 relapsed and 5 non-relapsed colon cancer patients. The volcano plot depicted in Fig. 5a shows the outcome. Three genes showing a significant increase of expression in relapsed patients compared to non-relapsed. The expression level of *OTOP2* (Fig. 5b) indicated a significant upregulation with 2.6 counts per 1000 cells in relapsed compared to 1.9 counts per 1000 cells for non-relapsed patients (p-value = 0.0042). The expression for the transcript *FGFR2* (Fig. 5c) showed 4.9 counts per 1000 cells for relapsed and 3.1 for non-relapsed patients (p-value = 0.0137). For *MMP11* significantly elevated expression levels for relapsed patients, with 49.2 counts per 1000 cells for relapsed and to 16.4 counts per 1000 cells for non-relapsed patients (p-value = 0.0415), were observed. A list with the p-values of all transcripts can be found in Additional file 1: Table S5.

Furthermore, we quantified differences of gene expression between relapsed and non-relapsed patients but omitted the spatial tissue compartments thereby simulating bulk RNA expression profiling. In the simulated bulk RNA expression data, *OTOP2* (p-value = 0.0167) and *MMP11* (p-value = 0.0177) remained significantly differentially upregulated in the relapse group, but *FGFR2* did not show significant changes (p-value = 0.1304) (Additional file 1: Fig. S12).

## Discussion

**Fig. 6:**
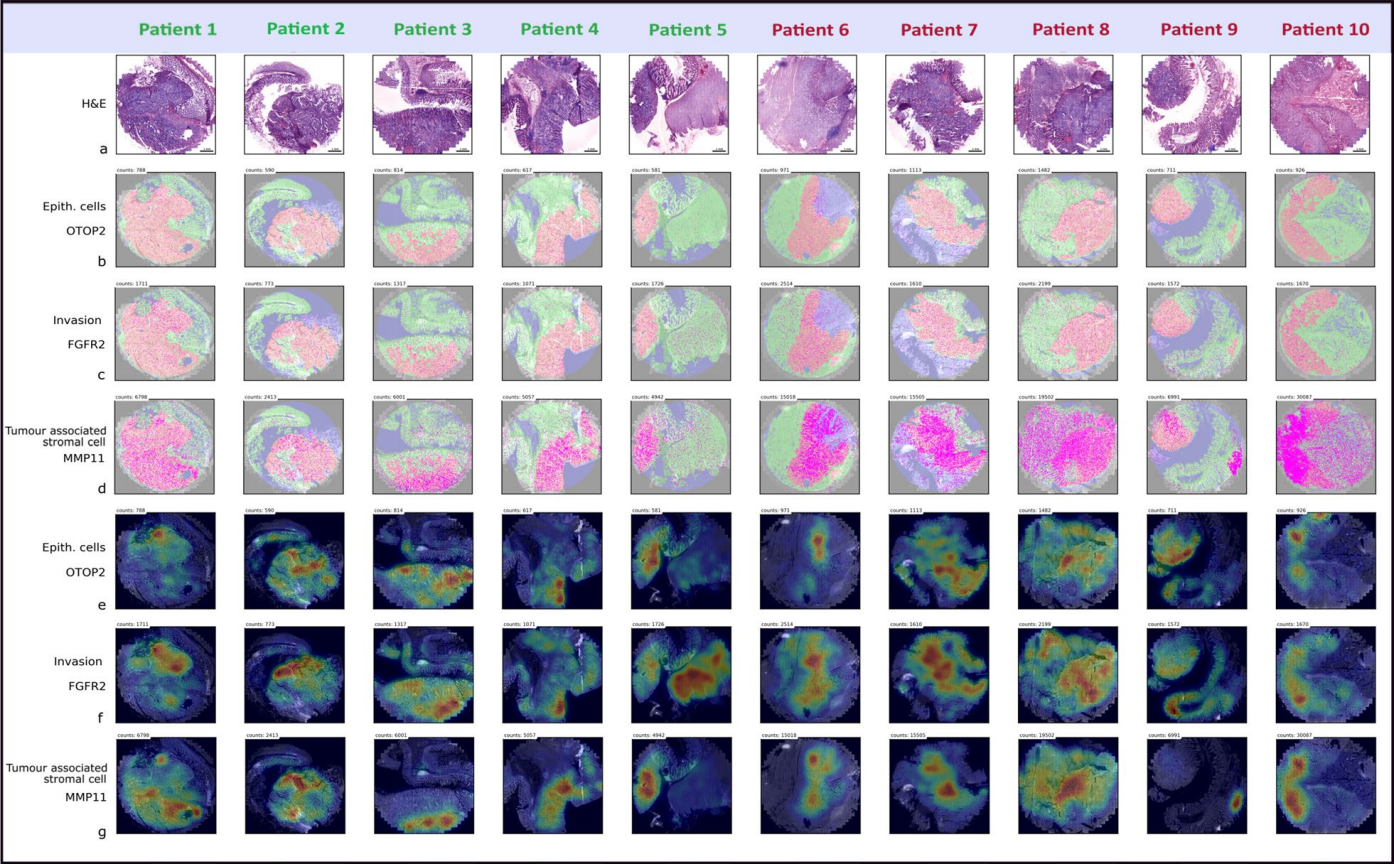
Spatial distribution and heatmaps of *OTOP2*, *FGFR2* and *MMP11*. **a** The virtually stained H&E images of the samples from non-relapsed (patient 1–5) and relapsed patients (patient 6–10). Expression and the spatial distribution of **b** *OTOP2*, **c** *FGFR2* and **d** *MMP11* and heatmaps of **e** *OTOP2*, **f** *FGFR2* and **g** *MMP11*. Total counts of each transcript are depicted in each image and size bar is the same for all images. The heatmaps visualize tumour heterogeneity, whereby each plot is normalised to its own maximum density value. The heat scale colour bar in e, patient 10 is the same for all heatmaps

With the here presented study we were the first to apply a direct RNA ISS approach in colon cancer with sub-cellular resolution investigating the spatial expression of 176 genes. We were able to identify *FGFR2*, *MMP11* and *OTOP2* as upregulated genes in tumour compartments of relapsed patients diagnosed with stage II colon cancer. Importantly, *FGFR2* and *MMP11* are druggable targets in other cancer entities and could become novel predictive biomarkers in stage II colon cancer. Moreover, we developed a genes-to-count (GTC) tool to accurately classify colon tissue into neoplastic and non-neoplastic compartments using an 8-gene tumour gene signature and to quantify spatial gene expression. The spatial ISS approach, therefore, allowed us to yield novel insights into predictive CRC biomarkers beyond bulk tissue sequencing (Fig. 6).

We observed a significantly elevated expression of the Fibroblast Growth Factor Receptor 2 (*FGFR2*) in tumour compartments of relapsed colon cancer stage II patients. Previous studied have identified this transcript as a potential therapeutic target for CRC as upon activation by ligand binding a series of downstream signalling pathways are activated involved in differentiation, survival and proliferation playing major roles in the progression of CRC [26, 27]. Interestingly, *FGFR2* has been shown to be druggable in other tumour entities. Pemigatinib and erdafitinib, two anti-*FGFR* agents, are already approved by the Food and Drug Administration (FDA) for treatment of cholangiocarcinoma and urothelial carcinoma, and various *FGFR* inhibitors are currently being evaluated in preclinical and clinical trials [28]. Due to overexpression of *FGFR2* in numerous tumours and its significant role in progression and tumorigenesis, *FGFR2* could be a promising target for treatment of stage II colon cancer patients in future [29]. Moreover, we observed significant upregulation for FGFR2 only for our spatial neoplastic compartment approach, whereas we did not identify significant changes in the simulated bulk RNA expression, i.e. expression/cell in the whole tissue section. Another interesting finding is that, although *FGFR2* is not differentially higher expressed in the neoplastic- versus non-neoplastic tissue compartment, its relative mRNA amount per cell is higher in the relapse group versus the non-relapse group (Fig. 5). This highlights the importance of quantification of spatial data sets yielding novel findings which would otherwise be easily overlooked by semiquantitative evaluation. Matrix metalloproteinase 11 (*MMP11*) belongs to the family of zinc dependent endopeptidases and displays some unique characteristics [30]. *MMP11* is secreted in an enzymatically active form while most other MMPs are released as inactive enzymes. It promotes the signal transduction of protein kinase B (*AKT*)/Forkhead box protein O1 (*FoxO1*)/insulin-like growth Factor-1 (I*GF1*) which is associated with the lysis of collagen type VI and proliferation of connective tissue around the stroma in cancerous tissues [30, 31]. Epithelial–mesenchymal-transition is a critical step in early stages of metastasis by granting tumor cells invasive potential and migratory behaviour, whereby metalloproteases play a crucial role in the degradation of ECM components. Upon the binding of NF-κB/p65 to the promoter regions of EMT transcription factors, MMP11 is activated resulting in the induction of the EMT process in human breast cancer cells [32]. These features indicate that *MMP11* plays a unique role in the development of malignant tumours, their progression and metastasis [30]. A previous study showed that *MMP11* is associated with various signalling pathways involved in tumour development in breast cancer and that high expression of *MMP11* is associated with poor prognosis for patients [33]. Additionally, *MMP11* overexpression is associated with an alteration in mitochondrial function due to increased oxidative stress and promotes a metabolic switch to aerobic glycolysis to provide metabolites for cancer cells [34]. Yang et al. identified *MMP11* as a key cancer driver in lung adenocarcinoma and, furthermore, as a potential target for antibody therapy as application of anti-*MMP11* antibodies suppressed the growth of tumours in xenograft models [35]. In contrast to *FGFR2* and *MMP11*, fewer published articles on Otopetrin 2 (*OTOP2*) in CRC are available. *OTOP2* encodes a proton-selective channel, transferring protons into the cell cytosol in response to low pH in various epithelia [36]. Recently, scRNAseq analysis identified a new subtype of cells positive for *OTOP2* and *BEST4* (calcium-sensitive chloride channel) within the intestinal crypts namely *BEST4/OTOP2* cells, that are responsible for electrolyte transportation [37]. In colorectal cancer and inflammation loss of *BEST4/OTOP2* cells has been described [37]. In our study we observed that *OTOP2* is overexpressed in relapsed stage II colon cancer patients. In contrast, Qu et al. and Guo et al. showed that elevated levels of *OTOP2* in cell line experiments lowers tumour proliferation and that high expression of *OTOP2* in bulk CRC tissues was significantly correlated with better overall survival. It is important to note, however, that the CRC cohort of Guo et al. did not focus on stage II colon cancer [38]. Therefore, the value of comparison between our cohort of stage II colon cancer and a broader cohort of CRC tissue and different stages is limited. Evaluation of tumour tissues and their histological compartments, such as tumour, stroma and immune cells need histological know-how and experience. Several powerful AI based tools have been evolved but these usually need large training sets to identify the respective tissue compartments [4]. Instead of AI based tissue classification, we made use of spatial ISS data to define tumour compartments, i.e. genes which are highly expressed in neoplastic colon tissues allowed us to generate tissue compartments of neoplastic tissue. A major advantage of the resulting compartments is that they are independent from tissue histology as they rely on gene expression only and can, therefore, be applied to other colon cancer samples without the need of large training image data sets. A similar approach was developed by Meylan et al. who identified tertiary lymphoid structures in renal cell carcinomas based on a 29-gene signature dominated by immune globulin genes, however with lower resolution of a bin size of 55 μm using Visium spatial transcriptomics (10× Genomics) [39]. When comparing our data of both approaches, i.e. morphology-based vs. expression-based, we are able to observe that the expression-based approach shows a more granular and precise representation of neoplastic tissue in three patient samples (patient 2, 3 and 10) especially at tumour border regions. Both approaches, morphological- and expression-based tissue compartments, show high concordance also in the context of differential gene expression of neoplastic vs. non-neoplastic tissue. All 81 upregulated genes identified using the morphological-based approach (neoplastic vs. non-neoplastic tissue compartments) were also identified in the expression-based approach confirming the equality in performance and precision of the created expression-based tissue compartments. Furthermore, by the use of the expression-based approach, 17 additional differentially expressed genes were found (total of 98).

Recognizing the extraordinary potential of spatial transcriptomic datasets in revealing detailed cellular- and tissue organization, data analysis remains challenging. A multitude of analysis and visualization tools for pre-processing, clustering, cell phenotyping, and cell–cell interaction are being developed continuously but a gold standard has yet to be set [40]. For example, segmentation of cells and assigning expressed transcripts to identify the underlying cell type can be performed by sophisticated methods such as Baysor [41], JSTA [42] or modified pipelines from pciSeq [43] and Scanpy [44, 45] among many others [46]. In our data the quality of ISS results strongly depended on tissue characteristics such as autofluorescence or fixation of tissue as optimal sensitivity and specificity of the ISS methodology requires bright, clear signals and low background [47]. Highly autofluorescent tissue structures such as elastin and collagen [48] can obscure or mimic in situ signals and would result in wrong base calling during decoding. Therefore, we developed and included a background subtraction step to reduce high autofluorescence especially for channels detected in shorter wavelengths such as FITC and Cy3. We observe an estimated ~ 25% higher number of correctly assigned transcripts with vs. without background subtraction. However, spatial analysis comes to its limitations if autofluorescent structures indicate higher pixel intensity values than true signals, as subtracting the background of these structures would results in a deletion of true signals (seen in a specific region in patient sample 6, described in Additional file 1: Fig. S10). In two additional patient samples, specific areas had to be excluded from further analysis, as tissue was lost during lab work. Nevertheless, these samples passed our quality control (threshold of expected/unexpected reads ratio) and enough representative tissue regions of neoplastic and non-neoplastic areas were available for these three patients (Additional file 1: Fig. S9).

Another obstacle in spatial transcriptomic data sets is the normalization of gene expression between samples. Available tools such as scran [49] or SCnorm [50] are inspired by scRNAseq studies but there is no “one-size-fits-all” solution [51] as they were not developed for sub-cellular ISS data sets. As dRNA-HybISS yields subcellular resolution, we normalised our expression data for each individual tumour sample. To do so, we segmented cells using available scripts and normalised the number of transcripts to cell counts. The segmentation of nucleus stained images and optimisation of parameters is crucial and highly dependent on the tissue type. Similar to previously described studies our approach is feasible and in accordance with our aim to investigate the spatial tissue composition of colon cancer stage II [52]. We demonstrated feasibility of in situ sequencing on clinical samples with a very focused sample cohort. The small sample size in this pilot study is comparable to other dRNA-HybISS studies performed by Janesick et al. and Svedlund et al. with < 10 samples. For further validation, larger cohort and complementary methods are needed, such as immunostaining, in vitro and in vivo studies [16, 53]. This is especially true for the three identified genes, *MMP11*, *FGFR2* and *OTOP2*, which need validation in an equivalent stage II colon cancer cohort with conventional immunostaining procedures.

This proof of principle study shows the potential of in situ sequencing revealing novel potential predictive biomarkers in colon cancer stage II, namely *MMP11*, *FGFR2* and *OTOP2*, relevant for relapse of disease. Furthermore, our newly developed, open-access available GTC-tool allows accurate capturing of the tumour compartment and quantification of gene expression in colon cancer tissue.

### Supplementary Information

**Additional file 1: Table S1:** List of clinical data. **Table S2:** List of designed gene panel. **Table S3:** Quality control of in situ sequencing data. The list contains the number and percentages of expected reads, unexpected reads and homomers of each sample calculated with a threshold of 0.1 from the MATLAB script (see methods section). **Table S4:** Parameter values used for microscope images of size (7660px, 7700px). **Table S5:** The resulting p-values for the statistical testing of relapse and non-relapse patients with the neoplastic tissue compartments. **Method S1:** virtual H&E image. **Method S2:** Compartment building by morphology. **Method S3:** Compartment building by gene expressions. **Method S4:** Gene set selection. **Method S5:** Gene counting in the compartments. **Method S6:** Excluding areas. **Method S7:** Statistical testing. **Fig. S1:** a) DAPI-stained image, b) FITC-stained image used for calculating and c) virtually stained H&E image of the tissue sample. **Fig. S2:** a) The tissue areas in the tissue sections as classified by a pathologist: red–neoplastic tissue, green–non-neoplastic tissue. The blue area marks a region that was excluded from the analysis due to high autofluorescence or lost tissue during hybridisation. The derived representative binary tissue compartment (TC) b) for the neoplastic and c) for the non-neoplastic tissue. d) The calculated TC combined in one image with the previously described colour coding. **Fig. S3:** Schematic example for the uniform kernel. **Fig. S4:** The density plots a) for FLT4, b) for BIK, c) for EREG and d) for MET. The areas with high density values (light red and yellow area) in b)-d) correlate with the areas of neoplastic tissue. **Fig. S5:** a) With the gene set S = {BIK, CCND1, CD44, EREG, ITGAV, MET, MYBL2, S100A4}, referred to as tumour gene signature, calculated tissue compartment for the neoplastic tissue. b) Hybridisation area compartment defined through a disk of radius centered in the middle of image. **Fig. S6:** Binary tissue compartments (TC) a) for all cells and b) for all ISS genes . c) The composite TC and d) the calculated representative non-neoplastic TC. **Fig. S7:** The calculated tissue compartments (TC) combined in one image: red–neoplastic TC and green–non-neoplastic TC. **Fig. S8:** The overlap (dark red) between the neoplastic tissue compartment (TC): dark red–overlap, light red–morphological-based TC with no overlap and pink–gene expression-based TC with no overlap. **Fig. S9:** Tissue damage (red) of patient sample 7 during the sequencing procedure. a) Virtually converted H&E staining of the sample. Image of tissue after b) the third c) the fourth and d) the fifth sequencing cycle. e) Shows the neoplastic and non-neoplastic compartments that were generated based on gene expression with the excluded area in blue. Tissue damage was observed after sequencing cycle 3-5. The red marked area had to be excluded from further analysis. **Fig. S10:** Highly autofluorescent areas observed in patient sample 6 (red) that showed higher pixel intensity values than truly positive signals were excluded from further analysis. a) Virtually converted H&E staining of the sample. b) Highly autofluorescent region was marked in red. c) Shows the neoplastic and non-neoplastic compartments that were generated based on gene expression with the excluded area in blue. **Fig. S11:** Alignment of spots: Alignment performed in CellProfiler by using ImageJ plugins MultiStackReg, StackReg and TurboReg. The figure shows a) correctly aligned spots and b) wrongly aligned spots. **Fig. S12:** Comparison of spatial analysis vs. bulk analysis: Volcano plots with a significance level α=0.05 of significantly upregulated genes in a) the neoplastic tissues compartment and b) the total tissue area of relapsed patients in comparison to non-relapsed patients. **Fig. S13:** Spatial distribution of 176 ISS detected transcripts.

## Data Availability

Data is openly available at the repository (https://github.com/spatialhisto/GTC). The raw tile images (several terabytes) are available from the corresponding author upon reasonable request.

## Abbreviations

CRC: Colorectal cancer
Cy3: Cyanine 3
Cy5: Cyanine 5
Cy7: Cyanine 7
DAPI: 4,6-Diamidino-2-phenylindole
dRNA-HybISS: Direct RNA-hybridisation based in situ sequencing
EMT: Epithelial–mesenchymal transition
FFPE: Formalin-fixed, paraffin-embedded
FITC: Fluorescein isothiocyanate
H&E: Hematoxylin and eosin stain
ISS: In situ sequencing
HybISS: Hybridisation based in situ sequencing
LED: Light-emitting diode
RCA: Rolling circle amplification
RCP: Rolling circle product
RNA: Ribonucleic acid
scRNA: Single cell RNA sequencing
TC: Tissue compartment
