# Supplementary material for: Spatial tumour gene signature discriminates neoplastic from non-neoplastic compartments in colon cancer: unravelling predictive biomarkers for relapse
Source: J Transl Med. 2023 Aug 5;21:528. doi: 10.1186/s12967-023-04384-0 (PMC10403907; doi:10.1186/s12967-023-04384-0)
Supplement: Supplementary file 1 — Additional file 1: Table S1: List of clinical data. Table S2: List of designed gene panel. Table S3: Quality control of in situ sequencing data. The list contains the number and percentages of expected reads, unexpected reads and homomers of each sample calculated with a threshold of 0.1 from the MATLAB script (see methods section). Table S4: Parameter values used for microscope images of size (7660px, 7700px). Table S5: The resulting p-values for the statistical testing of relapse and non-relapse patients with the neoplastic tissue compartments. Method S1: virtual H&E image. Method S2: Compartment building by morphology. Method S3: Compartment building by gene expressions. Method S4: Gene set selection. Method S5: Gene counting in the compartments. Method S6: Excluding areas. Method S7: Statistical testing. Fig. S1: a) DAPI-stained image, b) FITC-stained image used for calculating and c) virtually stained H&E image of the tissue sample. Fig. S2: a) The tissue areas in the tissue sections as classified by a pathologist: red–neoplastic tissue, green–non-neoplastic tissue. The blue area marks a region that was excluded from the analysis due to high autofluorescence or lost tissue during hybridisation. The derived representative binary tissue compartment (TC) b) for the neoplastic and c) for the non-neoplastic tissue. d) The calculated TC combined in one image with the previously described colour coding. Fig. S3: Schematic example for the uniform kernel. Fig. S4: The density plots a) for FLT4, b) for BIK, c) for EREG and d) for MET. The areas with high density values (light red and yellow area) in b)-d) correlate with the areas of neoplastic tissue. Fig. S5: a) With the gene set S = {BIK, CCND1, CD44, EREG, ITGAV, MET, MYBL2, S100A4}, referred to as tumour gene signature, calculated tissue compartment for the neoplastic tissue. b) Hybridisation area compartment defined through a disk of radius centered in the middle of image. Fig. S6: Binary tissue compartments (TC) a) [file 12967_2023_4384_MOESM1_ESM.docx]

***Table S1:* List of clinical data**

| ID | Recurrence 0=No  1=Yes | Age (at Surgery, in years) | Gender 0=Male 1=Female | Weight (in kg) | Date of Tumour OP | Tumour Location | T | Histo | Stage | Adjuvant CTX 0=No 1=Yes |
| --- | --- | --- | --- | --- | --- | --- | --- | --- | --- | --- |
| Patient 1 | 1 | 70 | 1 | 63 | 18.04.2010 | Flexura coli sinistra | 3 | Adenocarcioma | 2 | 0 |
| Patient 2 | 1 | 80 | 0 | 80 | 12.12.2012 | Colon transversum | 3 | Adenocarcioma | 2a | 0 |
| Patient 3 | 1 | 76 | 0 | 78 | 13.02.2013 | Rectum | 3 | Adenocarcioma | 2a | 0 |
| Patient 4 | 1 | 60 | 0 | 72 | 21.07.2010 | Caecum | 3 | Adenocarcioma | 2a | 0 |
| Patient 5 | 1 | 56 | 0 | 63 | 08.02.2010 | Colon transversum | 3 | Adenocarcioma | 2a | 0 |
| Patient 6 | 0 | 64 | 0 | 89 | 08.11.2010 | Left flexura | 3 | Adenocarcioma | 2a | 0 |
| Patient 7 | 0 | 74 | 1 | 77 | 12.05.2011 | Sigmoid | 3 | Adenocarcioma | 2a | 0 |
| Patient 8 | 0 | 27 | 0 | 77 | 26.03.2010 | Sigmoid | 3 | Adenocarcioma | 2a | 0 |
| Patient 9 | 0 | 73 | 1 | 80 | 10.07.2012 | Sigmoid | 3 | Adenocarcioma | 2a | 0 |
| Patient 10 | 0 | 70 | 1 | 61 | 20.12.2010 | Colon transversum | 3 | Adenocarcioma | 2 | 0 |

***Table S2:* List of designed gene panel**

| Biological process/group | | Subgroups | Gene (according to Human protein atlas [1,2]) | ENS number (derived from Ensembl genome browser) |
| --- | --- | --- | --- | --- |
| Angiogenesis | | | ANGPT1 | ENSG00000154188 |
|  |  |  | ANGPT2 | ENSG00000091879 |
|  |  |  | FLT4 | ENSG00000037280 |
|  |  |  | KDR (VEGFR-2) | ENSG00000128052 |
|  |  |  | TIE1 | ENSG00000066056 |
|  |  |  | EREG | ENSG00000124882 |
|  |  |  | ENG (CD105) | ENSG00000106991 |
|  |  |  | MET | ENSG00000105976 |
|  |  |  | CD248 (TEM1) | ENSG00000174807 |
|  |  |  | ADGRA2 (TEM5) | ENSG00000020181 |
|  |  |  | PLXDC1 (TEM7) | ENSG00000161381 |
|  |  |  | ANTXR1 (TEM8) | ENSG00000169604 |
|  |  |  | TEK | ENSG00000120156 |
|  |  |  | F8 | ENSG00000185010 |
| Apoptosis | | Pro-apoptotic | BBC3 (PUMA) | ENSG00000105327 |
|  |  |  | TNFSF10 (TRAIL) | ENSG00000121858 |
|  |  |  | BID | ENSG00000015475 |
|  |  |  | BIK | ENSG00000100290 |
|  |  |  | BCL2L11 (Bim) | ENSG00000153094 |
|  |  |  | BAK1 | ENSG00000030110 |
|  |  | Inhibitors | CFLAR (cFLIP) | ENSG00000003402 |
|  |  |  | BCL2L1 (BCLX) | ENSG00000171552 |
|  |  |  | MCL1 | ENSG00000143384 |
|  |  | Receptors | TNFRSF10A (DR4) | ENSG00000104689 |
|  |  |  | TNFRSF10B (DR5) | ENSG00000120889 |
|  |  | Caspases | CASP8 | ENSG00000064012 |
|  |  |  | CASP9 | ENSG00000132906 |
|  |  |  | CASP7 | ENSG00000165806 |
|  |  |  | CASP3 | ENSG00000164305 |
| Autophagy | | | MAP1LC3A (LC3) | ENSG00000101460 |
|  |  |  | ATG5 | ENSG00000057663 |
|  |  |  | BECN1 (Beclin 1) | ENSG00000126581 |
| Necrosis | | | RIPK3 | ENSG00000129465 |
|  |  |  | MLKL | ENSG00000168404 |
|  |  |  | HMGB1 | ENSG00000189403 |
|  |  |  | RIPK1 | ENSG00000137275 |
|  |  |  | PPIF | ENSG00000108179 |
| Proliferation | | Proliferation | PCNA | ENSG00000132646 |
|  |  |  | MCM2 | ENSG00000073111 |
|  |  |  | CNTD2 | ENSG00000105219 |
|  |  |  | EXOSC5 | ENSG00000077348 |
|  |  |  | E2F1 | ENSG00000101412 |
|  |  |  | MYBL2 | ENSG00000101057 |
|  |  |  | CCND1 | ENSG00000110092 |
|  |  |  | CCNE1 | ENSG00000105173 |
|  |  |  | GRB7 | ENSG00000141738 |
|  |  |  | RPS6KB1 | ENSG00000108443 |
|  |  |  | AURKA | ENSG00000087586 |
|  |  |  | SAAL1 | ENSG00000166788 |
|  |  |  | TGFA | ENSG00000163235 |
|  |  |  | EGFR | ENSG00000146648 |
|  |  |  | KIT | ENSG00000157404 |
|  |  |  | URGCP | ENSG00000106608 |
|  |  | Inhibitors | BOP1 | ENSG00000261236 |
|  |  |  | BTG2 | ENSG00000159388 |
|  |  |  | FRK | ENSG00000111816 |
| Oxidative stress | | | SOD1 | ENSG00000142168 |
|  |  |  | GPX1 | ENSG00000233276 |
|  |  |  | CAT | ENSG00000121691 |
|  |  |  | GSR | ENSG00000104687 |
|  |  |  | NOS2 | ENSG00000007171 |
|  |  |  | TXNL1 | ENSG00000091164 |
|  |  |  | PRDX2 | ENSG00000167815 |
|  |  |  | OSER1 | ENSG00000132823 |
| Hypoxia | | | HIF1A | ENSG00000100644 |
|  |  |  | HIF3A | ENSG00000124440 |
|  |  |  | EGLN3 | ENSG00000129521 |
|  |  |  | PDK1 | ENSG00000152256 |
|  |  |  | SLC2A1 (GLUT1) | ENSG00000117394 |
|  |  |  | HYOU1 | ENSG00000149428 |
| Stemness/Differentiation | | | ALDH1A1 | ENSG00000165092 |
|  |  |  | NANOG | ENSG00000111704 |
|  |  |  | SALL4 | ENSG00000101115 |
|  |  |  | CD44 | ENSG00000026508 |
|  |  |  | PROM1 (CD133) | ENSG00000007062 |
|  |  |  | BMI1 | ENSG00000168283 |
|  |  |  | MPL (CD110) | ENSG00000117400 |
|  |  |  | EPHB2 | ENSG00000133216 |
|  |  |  | LGR5 | ENSG00000139292 |
|  |  |  | IL6 | ENSG00000136244 |
|  |  |  | POU5F1 | ENSG00000204531 |
|  |  |  | SOX2 | ENSG00000181449 |
| Invasion | | | LAMC2 (Laminin-5, γ2) | ENSG00000058085 |
|  |  |  | ITGAV | ENSG00000138448 |
|  |  |  | L1CAM | ENSG00000198910 |
|  |  |  | MMP7 | ENSG00000137673 |
|  |  |  | ENAH (MENA) | ENSG00000154380 |
|  |  |  | FGFR2 | ENSG00000066468 |
|  |  |  | SCAI | ENSG00000173611 |
|  |  |  | MIEN1 | ENSG00000141741 |
|  |  |  | TIAM1 | ENSG00000156299 |
| Epithelial-Mesenchymal Transition | | | TJP1 | ENSG00000104067 |
|  |  |  | FN1 (Fibronectin) | ENSG00000115414 |
|  |  |  | TWIST1 | ENSG00000122691 |
|  |  |  | FOXC2 | ENSG00000176692 |
|  |  |  | ZEB1 | ENSG00000148516 |
|  |  |  | FAM3C | ENSG00000196937 |
| Energy metabolism | | | SLC2A1 (GLUT1) (already in table 1, other biological process) | ENSG00000117394 |
|  |  |  | HK1 | [ENSG00000156515](https://www.ensembl.org/Homo_sapiens/Location/View?r=10:69269984-69401884:1) |
|  |  |  | HK2 | ENSG00000159399 |
|  |  |  | PKM2 | ENSG00000067225 |
|  |  |  | LDHA | ENSG00000134333 |
|  |  |  | GLS | ENSG00000115419 |
|  |  |  | GLUD1 | ENSG00000148672 |
| OncotypeDX genes | | Cell cycle genes | MYBL2 (already in table 1, other biological process) | ENSG00000101057 |
|  |  | Stromal genes | BGN | ENSG00000182492 |
|  |  |  | INHBA | ENSG00000122641 |
|  |  | Early response gene | GADD45B | ENSG00000099860 |
| Epithelial cells | | Colonocytes (Enterocytes) | ANPEP | ENSG00000166825 |
|  |  |  | CA1 | ENSG00000133742 |
|  |  |  | CA2 | ENSG00000104267 |
|  |  |  | FABP1 | ENSG00000163586 |
|  |  |  | BEST4 | ENSG00000142959 |
|  |  |  | OTOP2 | ENSG00000183034 |
|  |  |  | GUCA2B | ENSG00000044012 |
|  |  |  | SLC26A3 | ENSG00000091138 |
|  |  | Goblet cells | MUC2 | ENSG00000198788 |
|  |  |  | MUC5AC | ENSG00000215182 |
|  |  |  | SPDEF | ENSG00000124664 |
|  |  |  | KLF4 | ENSG00000136826 |
|  |  |  | TFF1 | ENSG00000160182 |
|  |  |  | TFF3 | ENSG00000160180 |
|  |  | Enteroendocrine cells | CHGA | ENSG00000100604 |
|  |  |  | TBXT | ENSG00000164458 |
|  |  |  | ZGLP1 | ENSG00000220201 |
|  |  |  | GIP | ENSG00000159224 |
|  |  |  | SST | ENSG00000157005 |
|  |  |  | NTS | ENSG00000133636 |
|  |  |  | PYY | ENSG00000131096 |
|  |  |  | GAST | ENSG00000184502 |
|  |  |  | MLN | ENSG00000096395 |
|  |  | Tuft cells | DCLK1 | ENSG00000133083 |
|  |  |  | TRPM5 | ENSG00000070985 |
|  |  |  | POU2F3 | ENSG00000137709 |
|  |  |  | GFI1B | ENSG00000165702 |
|  |  | Stem cells | OLFM4 | ENSG00000102837 |
|  |  |  | CD44 (already in table 1, other biological process) | ENSG00000026508 |
|  |  |  | PROM1 (CD133) (already in table 1, other biological process) | ENSG00000007062 |
|  |  |  | LGR5 (already in table 1, other biological process) | ENSG00000139292 |
|  |  |  | ALDH1A1 (already in table 1, other biological process) | ENSG00000165092 |
| Tumor-associated stromal cells (TASC) | Cancer- associated fibroblasts (CAFs) | Mesenchymal stem cell-like | CD105 (already in table 1, other biological process) | ENSG00000106991 |
|  |  |  | THY1 (CD90) | ENSG00000154096 |
|  |  |  | NT5E (CD73) | ENSG00000135318 |
|  |  |  | CD44 (already in table 1, other biological process) | ENSG00000026508 |
|  |  | Endothelial-like | ICAM1 (CD54) | ENSG00000090339 |
|  |  |  | TEK (TIE2) | ENSG00000120156 |
|  |  |  | VCAM1 (CD106) | ENSG00000162692 |
|  |  |  | CDH5 (CD144) | ENSG00000179776 |
|  |  | MyoFibroblast-like | TNC | ENSG00000041982 |
|  |  |  | TAGLN | ENSG00000149591 |
|  |  |  | PDGFA | ENSG00000197461 |
|  |  |  | TGFB3 | ENSG00000119699 |
|  |  | Pericyte-like | CSPG4 (NG2) | ENSG00000173546 |
|  |  | Matrix remodelling | S100A4 (FSP1) | ENSG00000196154 |
|  |  |  | MMP2 | ENSG00000087245 |
|  |  |  | DCN | ENSG00000011465 |
|  |  |  | COL1A2 | ENSG00000164692 |
|  |  | others | FSTL1 | ENSG00000163430 |
|  |  |  | TIMP1 | ENSG00000102265 |
|  |  |  | LIF | ENSG00000128342 |
|  |  |  | IL11 | ENSG00000095752 |
|  | Cancer-associated adipocytes (CAAs) | | IGFBP2 | ENSG00000115457 |
|  |  |  | MMP11 | ENSG00000099953 |
|  |  |  | IL6 (already in table 1, other biological process) | ENSG00000136244 |
|  |  |  | IL1B | ENSG00000125538 |
|  |  |  | IL8 | ENSG00000169429 |
|  |  |  | FABP4 | ENSG00000170323 |
|  | Cancer-associated endothelial cells (CAECs) | | S100A4 (FSP1) (already in table 1, other biological process) | ENSG00000196154 |
|  |  |  | CXCL1 | ENSG00000163739 |
|  |  |  | CD248 (TEM1) (alredy in list) | ENSG00000174807 |
|  |  |  | ADGRA2 (TEM5) (already in table 1, other biological process) | ENSG00000020181 |
|  |  |  | PLXDC1 (TEM7) (already in table 1, other biological process) | ENSG00000161381 |
|  |  |  | ANTXR1 (TEM8) (already in table 1, other biological process) | ENSG00000169604 |
|  | metastasis-associated fibroblasts (MAFs) | | CXCL12 (SDF1) | ENSG00000107562 |
| Th1 response | | | TNF | ENSG00000232810 |
| Macrophages /MDSC gene profile | | | NOS1 | ENSG00000089250 |
|  |  |  | IL8 (already in table 1, other biological process) | ENSG00000169429 |
|  |  |  | CD83 | ENSG00000112149 |
|  |  |  | CD86 | ENSG00000114013 |
| T cell inhibition | | | IDO2 | ENSG00000188676 |
|  |  |  | IL7R | ENSG00000168685 |
|  |  |  | BTLA | ENSG00000186265 |
|  |  |  | NCAM1 | ENSG00000149294 |
| General immunosuppression | | | BTLA (already in table 1, other biological process) | ENSG00000186265 |
|  |  |  | IDO2 (already in table 1, other biological process) | ENSG00000188676 |
| Inflammatory | | | NOS2 (already in table 1, other biological process) | ENSG00000007171 |
|  |  |  | CD83 (already in table 1, other biological process) | ENSG00000112149 |
| Natural killer cells | | | NCR1 | ENSG00000189430 |
|  |  |  | MICA | ENSG00000204520 |
|  |  |  | MICB | ENSG00000204516 |
|  |  |  | KLRK1 | ENSG00000213809 |
|  |  |  | NCAM1 (already in table 1, other biological process) | ENSG00000149294 |
| Dendritic cells | | | ITGAM | ENSG00000169896 |

***Table S3: Quality control of in situ sequencing data.*** The list contains the number and percentages of expected reads, unexpected reads and homomers of each sample calculated with a threshold of 0.1 from the MATLAB script (see methods section).

|  | total counts with threshold 0.1 | | | Percentage distribution (%) | | | reads in total |
| --- | --- | --- | --- | --- | --- | --- | --- |
|  | expected reads | unexpected reads | homomer | expected reads | unexpected reads | homomer |  |
| **183KS** | 891999 | 254404 | 62085 | 74 | 21 | 5 | 1208488 |
| **184KS** | 985008 | 390797 | 105300 | 67 | 26 | 7 | 1481105 |
| **186KS** | 942977 | 365210 | 20150 | 71 | 27 | 2 | 1328337 |
| **189KS** | 992282 | 269708 | 31233 | 77 | 21 | 2 | 1293223 |
| **190KS** | 723538 | 277071 | 33743 | 70 | 27 | 3 | 1034352 |
| **180KS** | 1072757 | 372653 | 124511 | 68 | 24 | 8 | 1569921 |
| **181KS** | 1684402 | 756442 | 120427 | 66 | 30 | 5 | 2561271 |
| **185KS** | 1697772 | 624136 | 116621 | 70 | 26 | 5 | 2438529 |
| **188KS** | 1337611 | 556580 | 21780 | 70 | 29 | 1 | 1915971 |
| **192KS** | 804678 | 251427 | 17182 | 75 | 23 | 2 | 1073287 |

*Summarization of the main steps of the computationally analysis:*

*Morphology-based method: A pathologist, to whom the virtually stained H&E images were shown, coloured the tissue areas which he classified as neoplastic and as non-neoplastic in high detail with the colours red and green by using a drawing software (see Fig. S2). These coloured H&E images were then used to build the binary compartments for the neoplastic and non-neoplastic tissue:*

*The binary compartment for the neoplastic tissue is a black (represented by the value 0) and white (represented by the value 1) image, where the white areas are identical to neoplastic areas classified by the pathologist.*
 *The binary compartment for the non-neoplastic tissue is a black and white image, where the white areas represent the non-neoplastic tissue that are identical to the non-neoplastic areas classified by the pathologist.*
*The binary compartments can be used to determine whether a transcript of a gene lies in, e.g., the neoplastic tissue: by looking up if the pixel at the position of the transcript in the binary neoplastic compartment is white or black. Hence, binary tissue compartments can be used to count the number of transcripts within the neoplastic and non-neoplastic tissue.*

*Gene expression based method: The transcripts of the genes of the gene set are represented as dots of a certain diameter. If all the transcripts, e.g., within the binary neoplastic compartment, are overlapped, connected areas are formed by the dots, i.e., in regions with a high transcript density. The threshold then can be used to define how high the transcript density must be for connected-areas to appear. The connected areas can then be used to obtain binary tissue compartments.*
*The transcripts of certain genes appear especially in the neoplastic tissue and hence can be used for the automatically tissue classification where the transcripts of the genes of a gene set are used for the building of the connected areas.*

*Gene-expression based tissue compartments are calculated with gene sets of varying length and composition. For each set, the neoplastic tissue compartments is calculated for all tissue samples. Afterwards, the overlap between the tissue compartment and the morphology-based compartment of each sample is calculated. There, a high overlap value means a high congruence between the two compartments. A combined overlap value of all samples is then calculated for a gene set. Thereafter, all gene sets are ranked according to their combined overlap and the gene set with the highest value is chosen for the analysis.*

**Method S1: virtual H&E image**

The microscopic images obtained with DAPI and FITC excitation (see Fig. S1) can be used to produce a similar dyed image with the computer as one would get using the H&E staining technique in the laboratory. For this, every pixel value of the grey-scale DAPI image (which ranges from 0 to 255) is multiplied with a fixed rgb-colour vector, representing the haematoxylin staining, while each pixel of the FITC image is multiplied with a fixed rgb-colour vector representing the eosin staining. When these two pictures are combined, a so-called “virtually stained” image is obtained. This can be done since the DAPI excitation holds the information about the cell nuclei and the FITC excitation autofluorescence holds the information about the tissue.

The virtually stained hematoxylin and eosin (H&E) image, as shown in Fig. S1, is calculated from the DAPI-stained and the FITC-stained image using the method described in [3]:

| $\begin{matrix} R_{i,j}=exp[-k\left( a_{R}*D_{i,j}+b_{R}*F_{i,j} \right)] \\ G_{i,j}=exp[-k\left( a_{G}*D_{i,j}+b_{G}*F_{i,j} \right)] \\ B_{i,j}=exp[-k(a_{B}*D_{i,j}+b_{B}*F_{i,j})] \end{matrix}$ | (1) |
| --- | --- |

wherein $R_{i,j}$, $G_{i,j}$ and $B_{i,j}$ are the pixels of the red, the green and the blue channel of the H&E image, $D_{i,j}$ and $F_{i,j}$ are the pixels from the grey channel of the DAPI and the FITC image, respectively. The constants $k$, $a=\left( a_{R}, a_{G}, a_{B} \right)$ and $b=\left( b_{R}, b_{G}, b_{B} \right)$ are used for the colour and contrast adjustment of the virtually stained H&E image.


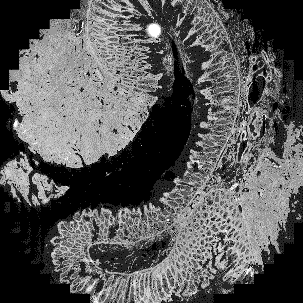

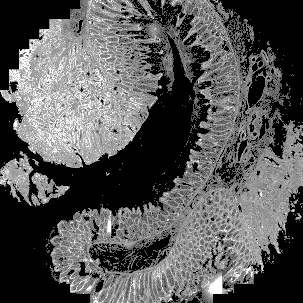

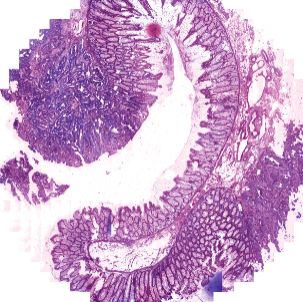


**c**

**b**

**Fig. S1**: a) DAPI-stained image, b) FITC-stained image used for calculating and c) virtually stained H&E image of the tissue sample.

**a**

1mm

1mm

1mm

The parameters $a=\left( 0.30, 1.00, 0.86 \right)$ and $b=\left( 0.54, 1.00, 0.05 \right)$ are kept constant while the value of $k$ changes with each tissue sample so that a maximum value $p<255$ is not exceeded in $R_{i,j}$, $G_{i,j}$ and $B_{i,j}$.

The virtually stained H&E images are then shown to a pathologist specialised on CRC, who classified the tissue in certainly neoplastic and non-neoplastic areas, i.e. by using the colours red and green respectively (see Fig. S2).

**Method S2: Compartment building by morphology**

For the computational tissue compartment (TC) building, areas with same colour are converted together into the same binary TC $C_{i,j}^{\mathrm{tissue}}$, as shown in Fig. S2. Thereby, $C_{i,j}^{\mathrm{tissue}}$ holds only the values 0 and 1 for all pixels

| $C_{i,j}^{\mathrm{tissue}}= \left\{ \begin{matrix} 1 & TC in pixel \left( i,j \right) \\ 0 & TC not in pixel (i,j) \end{matrix} \right.$ | (2) |
| --- | --- |

To take into account that particular areas $B_{i,j}$ of the image cannot be used for the analysis, e.g. due to too high autofluorescence of the tissue or lost tissue during hybridisation, these areas have to be excluded from all TC, i.e. by marking them with the colour blue as shown in Fig. S2:

| $C_{i,j}^{\mathrm{analysis}}=C_{i,j}^{\mathrm{tissue}} \backslash B_{i,j}$ | (3) |
| --- | --- |


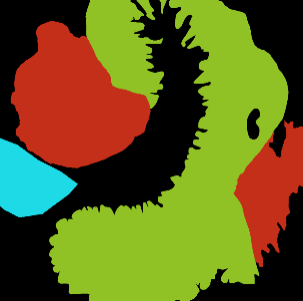

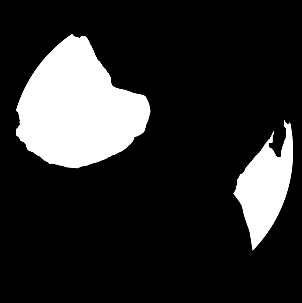

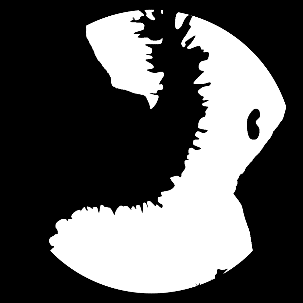

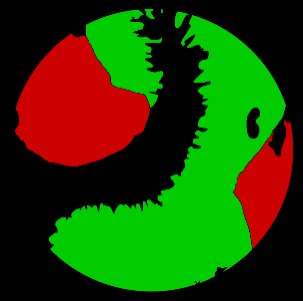


**c**

**b**

**Fig S2:** a) The tissue areas in the tissue sections as classified by a pathologist: red – neoplastic tissue, green – non-neoplastic tissue. The blue area marks a region that was excluded from the analysis due to high autofluorescence or lost tissue during hybridisation. The derived representative binary tissue compartment (TC) b) for the neoplastic and c) for the non-neoplastic tissue. d) The calculated TC combined in one image with the previously described colour coding.

**a**

**d**

1mm

1mm

1mm

1mm

**Method S3: Compartment building by gene expressions**

In a pre-step, image analyses are performed on both microscope images of the pixel size *(*$I, J)$, on the *in situ* sequencing (ISS) images and on the DAPI-stained images:

The fractional image coordinates $\vec{x}_{c}$ for the nuclei $c$ centre positions of the cells $C$ and with 0<$\vec{x}_{c}$<1 are determined with the software tool CellProfiler™ through segmentation analysis. Further, the fractional image coordinates $\vec{x}_{g}$ for the positions of the ISS transcripts $g$ of gene $G$, e.g. HK1, are determined with the software MATLAB®.

Utilising $\vec{x}_{g}$, the distribution of all transcripts of gene $G$ in the tissue sample can be visualized through a density plot. For this purpose, each transcript is represented as disk-shaped element of radius $r$ with $\vec{x}_{g}$ as its centre’ coordinate. A transcript’s density $\rho_{i,j}^{g}$ at the pixel $(i,j)$ with the centre’ coordinate $\vec{x}_{i,j}$ is then calculated by applying a uniform distribution kernel (see Fig. S3):

| $\rho_{i,j}^{g}(r)=\left\{ \begin{matrix} \frac{1}{2\pi r^{2}} & \left\vert\vec{x}_{i,j}-\vec{x}_{g} \right\vert<r \\ 0 & \left\vert\vec{x}_{i,j}-\vec{x}_{g} \right\vert\geq r \end{matrix} \right.$ | (4) |
| --- | --- |

| 0.0 | 0.0 | 0.0 | 1.0 | 0.0 | 0.0 | 0.0 |
| --- | --- | --- | --- | --- | --- | --- |
| 0.0 | 1.0 | 1.0 | 1.0 | 1.0 | 1.0 | 0.0 |
| 1.0 | 1.0 | 1.0 | 1.0 | 1.0 | 1.0 | 1.0 |
| 1.0 | 1.0 | 1.0 | 1.0 | 1.0 | 1.0 | 1.0 |
| 1.0 | 1.0 | 1.0 | 1.0 | 1.0 | 1.0 | 1.0 |
| 0.0 | 1.0 | 1.0 | 1.0 | 1.0 | 1.0 | 0.0 |
| 0.0 | 0.0 | 0.0 | 1.0 | 0.0 | 0.0 | 0.0 |

**Fig S3:** Schematic example for the uniform kernel.

The density distribution $\rho_{i,j}^{G}$ for all transcripts of gene $G$ in the image is then given by the sum of all single transcript densities $\rho_{i,j}^{g}$:

| $\rho_{i,j}^{G}(r)=\sum_{\forall g\in G} \rho_{i,j}^{g}(r)$ | (5) |
| --- | --- |

When the obtained density plots, as shown in Fig. S4, are compared with the stained image of the tissue sample, some genes show a more or uniform density distribution in the whole tissue sample, e.g. FLT4 in neoplastic and non-neoplastic, while others show high densities only in certain tissue areas, e.g. MET predominately in neoplastic tissue.

For some genes, the high-density areas reveal a good correlation with the neoplastic tissue, as classified by a pathologist specialised on CRC in view of its morphological appearance. For example, the comparison of Fig. S2 and Fig S4 shows that the distribution of BIK, EREG and MET can be associated with the neoplastic tissue.


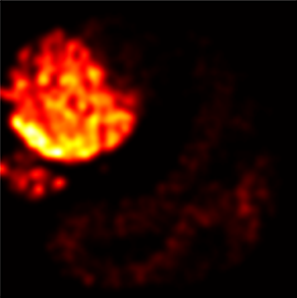


**d**


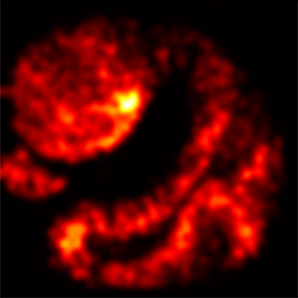

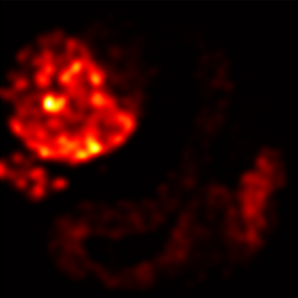

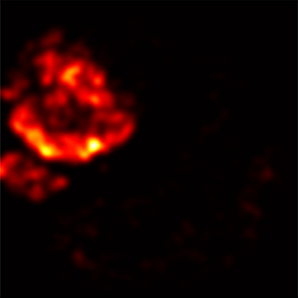


***Fig. S4*:** The density plots a) for FLT4, b) for BIK, c) for EREG and d) for MET. The areas with high density values (light red and yellow area) in b)-d) correlate with the areas of neoplastic tissue.

**a**

**c**

**b**

1mm

1mm

1mm

1mm

Based on this observation, a set of selected genes can be used for the automatic classification of the neoplastic area in tissue sections. Consequently, the remaining tissue represents then the non-neoplastic tissue.

In a first step, the genes with a high correlation are identified and combined to a gene set $S=\{G_{1},G_{2},\ldots,G_{n}\}$. In a second step, the densities $\rho_{i,j}^{G}$ of the genes in $S$ are summed up to a total gene set density $\rho_{i,j}^{S}$, whereat $r$ is kept constant for all genes part of the set:

| $\rho_{i,j}^{S}(r)=\sum_{\forall G\in S} \rho_{i,j}^{G}(r)$ | (6) |
| --- | --- |

Next, in order to convert the combined density plot into a TC $T_{i,j}$, a threshold is applied to the density value of each pixel $(i,j)$:

| $T_{i,j}=\left\{ \begin{matrix} 1 & \vartheta\leq\rho_{i,j}^{S}(r) \\ 0 & \vartheta>\rho_{i,j}^{S}(r) \end{matrix} \right.$ | (7) |
| --- | --- |

Therein, the absolute threshold value $\vartheta$ is given as product of the relative threshold $\theta$ with the maximum value of $\rho_{i,j}^{G}$ in the whole image:

| $\begin{matrix} \vartheta=\theta\cdot\max_{I, J} [\rho_{i,j}^{S}] & \mathrm{with}0\leq\theta\leq1 \end{matrix}$ | (8) |
| --- | --- |

The threshold values used for the calculations are summarized in table 1.


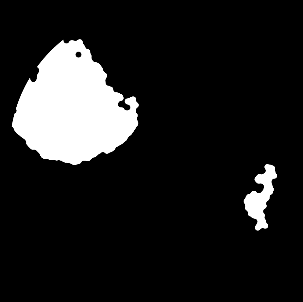

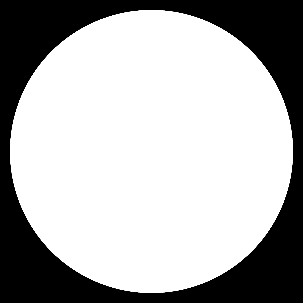


***Fig. S5***: a) With the gene set *S = {BIK, CCND1, CD44, EREG, ITGAV, MET, MYBL2, S100A4},* referred to as tumour gene signature, calculated tissue compartment $T_{i,j}$ for the neoplastic tissue. b) Hybridisation area compartment $S_{i,j}$ defined through a disk of radius $R$ centered in the middle of image.

**a**

**b**

1mm

1mm

Disregarding the number of detected ISS transcripts and thus the resulting $\rho_{i,j}^{S}$, the values of the disk radius$r$ and of the relative threshold parameter $\theta$ determine the resolution of details of the generated TC. By increasing $r$, more and more disks merge, forming larger and connected areas. However, above a certain value, too many details of the tissue sample are lost. Similarly, a too high $\theta$ also causes a loss of details, while a too low value falsely depicts the TC by dispersed or displaced genes. With $\theta=0.0$, all pixels $\rho_{i,j}^{S}>0$ are part of the TC. The parameter values used for the calculations in this paper are summarized in table 1.

Further, to achieve an improvement of the binary TC, morphological transformations from the python library openCV [4] are used to fill up small gaps in the TC, i.e. by a closing operation followed by an immediate opening operation.

***Table S4*:** **Parameter values used for microscope images of size (7660px, 7700px).**

| Tissue compartment | Disk radius *r* | Relative threshold $\theta$ |
| --- | --- | --- |
| neoplastic | 180px | 0.10 |
| composite cells | 50px | 0.00 |
| composite ISS genes | 50px | 0.00 |

During the ISS hybridisation of the tissue, a secure-seal hybridization chamber (Sigma-Adrich) is mounted on every tissue sample. However, as the tissues are not well covered by the hybridisation solution near the chamber boundary, the number of detected transcripts is not representative in this area. Hence, a secure area $S_{i,j}$ with a certain indention from the secure seal boundary is defined where the number of detected transcripts is assumed to be representative for the tissue (see Fig. S5).

Thus, the secure area is defined as disk-shaped compartment:

| $S_{i,j}(R)=\left\{ \begin{matrix} 1 & \left\vert\vec{x}_{i,j}-\vec{C} \right\vert<R \\ 0 & \left\vert\vec{x}_{i,j}-\vec{C} \right\vert\geq R \end{matrix} \right.$ | (9) |
| --- | --- |

where $\vec{C}=\left( I/2,J/2 \right)$ and $R$ represent the centre of the image and the radius of the compartment, respectively.

The representative TC $C_{i,j}^{\mathrm{tissue}}$ is then given through the intersection of $S_{i,j}$ with $T_{i,j}$*:*

| $C_{i,j}^{\mathrm{tissue}}=T_{i,j}\cap S_{i,j}(R)$ | (10) |
| --- | --- |

The non-neoplastic TC is obtained by building a compartment for the whole (composite) tissue sample $C_{i,j}^{\mathrm{composite}}$ and then subtracting the neoplastic one.

First, $C_{i,j}^{\mathrm{composite}}$ is calculated through intersection of the compartment $C_{i,j}^{ISS genes}$ obtained from all detected ISS transcripts (of all genes) and the compartment $C_{i,j}^{\mathrm{cells}}$ derived from all cell nuclei positions, as shown in Fig. S6:

| $T_{i,j}^{\mathrm{composite}}=T_{i,j}^{\mathrm{cells}}\cap T_{i,j}^{ISS genes}$ | (11) |
| --- | --- |

This approach reflects the fact that both tissue and ISS padlock reagents must be present for the correct detection of a gene. The parameters used for the calculations of the compartments are shown in table 1. Thereby, $T_{i,j}^{ISS genes}$ as well as $T_{i,j}^{\mathrm{cells}}$ are calculated with the same procedure as described above for the neoplastic TC. However, due to the high number of observed ISS genes and cell nuclei, smaller values are used for $r$ and $\theta$ to preserve the details of the tissue sample.


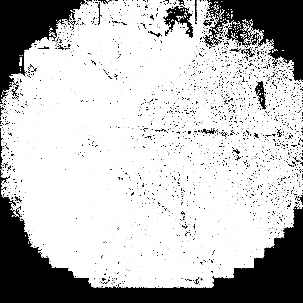

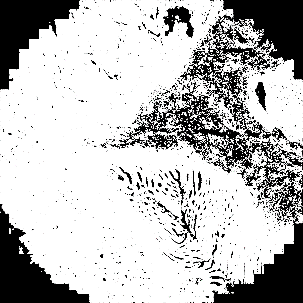


***Fig. S6*:** Binary tissue compartments (TC) a) for all cells $T_{i,j}^{\mathrm{cells}}$ and b) for all ISS genes $T_{i,j}^{ISS genes}$. c) The composite TC $C_{i,j}^{\mathrm{composite}}$ and d) the calculated representative non-neoplastic TC.

**a**

**b**


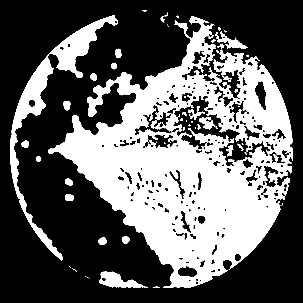

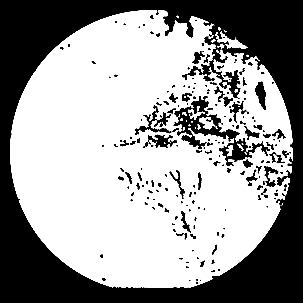


**c**

**d**

1mm

1mm

1mm

1mm

The secure area of the composite TC $C_{i,j}^{\mathrm{composite}}$ is obtained by intersection of $T_{i,j}^{\mathrm{composite}}$ with the secure area compartment $S_{i,j}$, as described for the neoplastic tissue. In the next step, the representative non-neoplastic binary TC $C_{i,j}^{non-neoplastic}$ is calculated through subtraction of the neoplastic TC from the composite TC:

| $C_{i,j}^{non-neoplastic}=C_{i,j}^{\mathrm{composite}} \backslash C_{i,j}^{\mathrm{neoplastic}}$ | (12) |
| --- | --- |

To take into account that particular areas of the image $B_{i,j}$ cannot be used for the analysis, as described in the previous chapter, these areas have to be excluded from $C_{i,j}^{non-neoplastic}$ and $C_{i,j}^{\mathrm{neoplastic}}$:

| $C_{i,j}^{\mathrm{analysis}}=C_{i,j} \backslash B_{i,j}$ | (13) |
| --- | --- |

Finally, Fig. S7 shows both obtained TC in one image whereby the same colour coding is used as in Fig. S2.


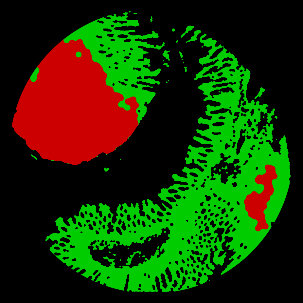


***Fig. S7*:** The calculated tissue compartments (TC) combined in one image: red – neoplastic TC and green – non-neoplastic TC.

1mm

**Method S4: Gene set selection**

The overlap $O$ between the morphological and the gene expression-based neoplastic TC, as shown in Fig. S8, is calculated for an image of the pixel size ($I, J)$ via:

| $O=\frac{1}{A_{\mathrm{morph}}^{\mathrm{neoplastic}}A_{\mathrm{gene}}^{\mathrm{neoplastic}}}\sum_{\begin{aligned} \forall i\in I, \\ \forall j\in J \end{aligned}} \left( C_{i,j, \mathrm{morph}}^{\mathrm{neoplastic}}\cap C_{i,j,\mathrm{gene}}^{\mathrm{neoplastic}} \right)^{2}$ | (14) |
| --- | --- |

where $A_{\mathrm{morph}}^{\mathrm{neoplastic}}$ and $A_{\mathrm{gene}}^{\mathrm{neoplastic}}$ are the areas of the morphological and the gene expression-based neoplastic TC, respectively. The TC areas within are obtained by summing up all pixels of the TC:

| $A^{\mathrm{tissue}}=\sum_{\begin{aligned} \forall i\in I, \\ \forall j\in J \end{aligned}} C_{i,j}^{\mathrm{tissue}}$ | (15) |
| --- | --- |


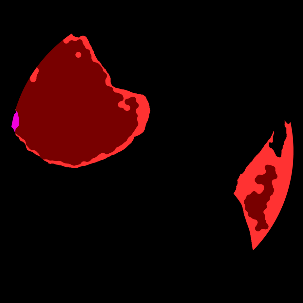


***Fig. S8:*** The overlap (dark red) between the neoplastic tissue compartment (TC): dark red – overlap, light red – morphological-based TC with no overlap and pink – gene expression-based TC with no overlap.

1mm

The composition of the gene set $S$ is alternated and the overlaps are calculated for all $N$ patient samples. The mean overlap used is then used for the rating of $S$:

| $O_{\mathrm{mean}}=\sqrt[N]{O_{1}O_{2}\ldots O_{N}}$ | (16) |
| --- | --- |

Finally, the gene set consisting of the 8 genes *S = {BIK, CCND1, CD44, EREG, ITGAV, MET, MYBL2, S100A4}* and referred to as tumour gene signature, achieved the highest $O_{\mathrm{mean}}$ value is therefore selected for statistical testing.

**Method S5: Gene counting in the compartments**

The following function is used for the counting of transcripts or cells within a specific TC$C^{\mathrm{tissue}}$ in which $\vec{x}_{g}$ is the fractional coordinate of the transcript’s position in the image:

| $f\left( \vec{x}_{g},C^{\mathrm{tissue}} \right)= \left\{ \begin{matrix} 1 & \vec{x}_{g}\in C^{\mathrm{tissue}} \\ 0 & \vec{x}_{g}\notin C^{\mathrm{tissue}} \end{matrix} \right.$ | (17) |
| --- | --- |

The number of transcripts of a certain gene $G$ per TC is then given through:

| $N_{G}^{\mathrm{tissue}}=\sum_{\forall g\in G} f(\vec{x}_{g,}C^{\mathrm{tissue}})$ | (18) |
| --- | --- |

Similarly, the number of cells per compartment is given by:

| $N_{C}^{\mathrm{tissue}}=\sum_{\forall c\in C} f(\vec{x}_{c,}C^{\mathrm{tissue}})$ | (19) |
| --- | --- |

The density per cell $D_{G,C}^{\mathrm{tissue}}$ for the transcripts of gene $G$ is then given by:

| $D_{G}^{\mathrm{tissue}}=N_{G}^{\mathrm{tissue}}/N_{C}^{\mathrm{tissue}}$ | (20) |
| --- | --- |

**Method S6: Excluding areas**

Some areas had to be excluded from analysis due to tissue damage during the sequencing procedure, high autofluorescence of specific structures or wrong alignment. If this areas would have not been excluded, this could result in wrong base calling and detection of false positive genes.

- Tissue damage seen in patient sample 7


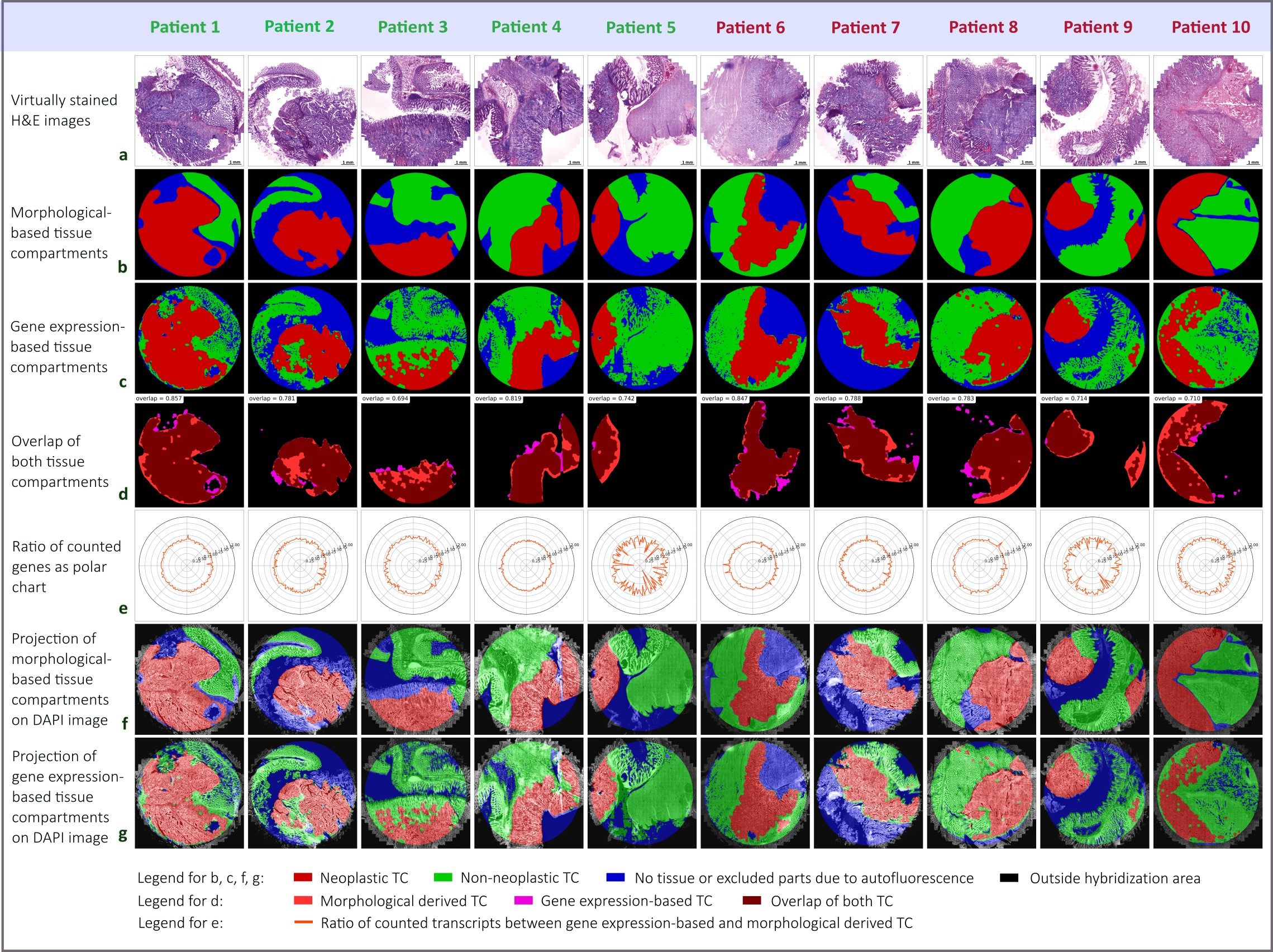

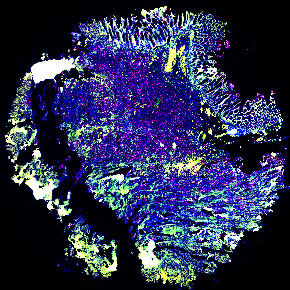

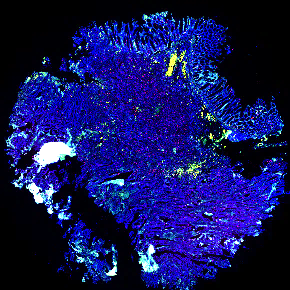

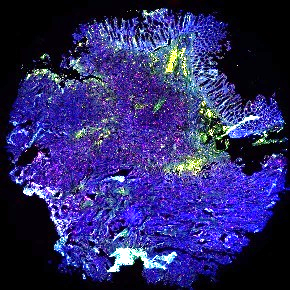

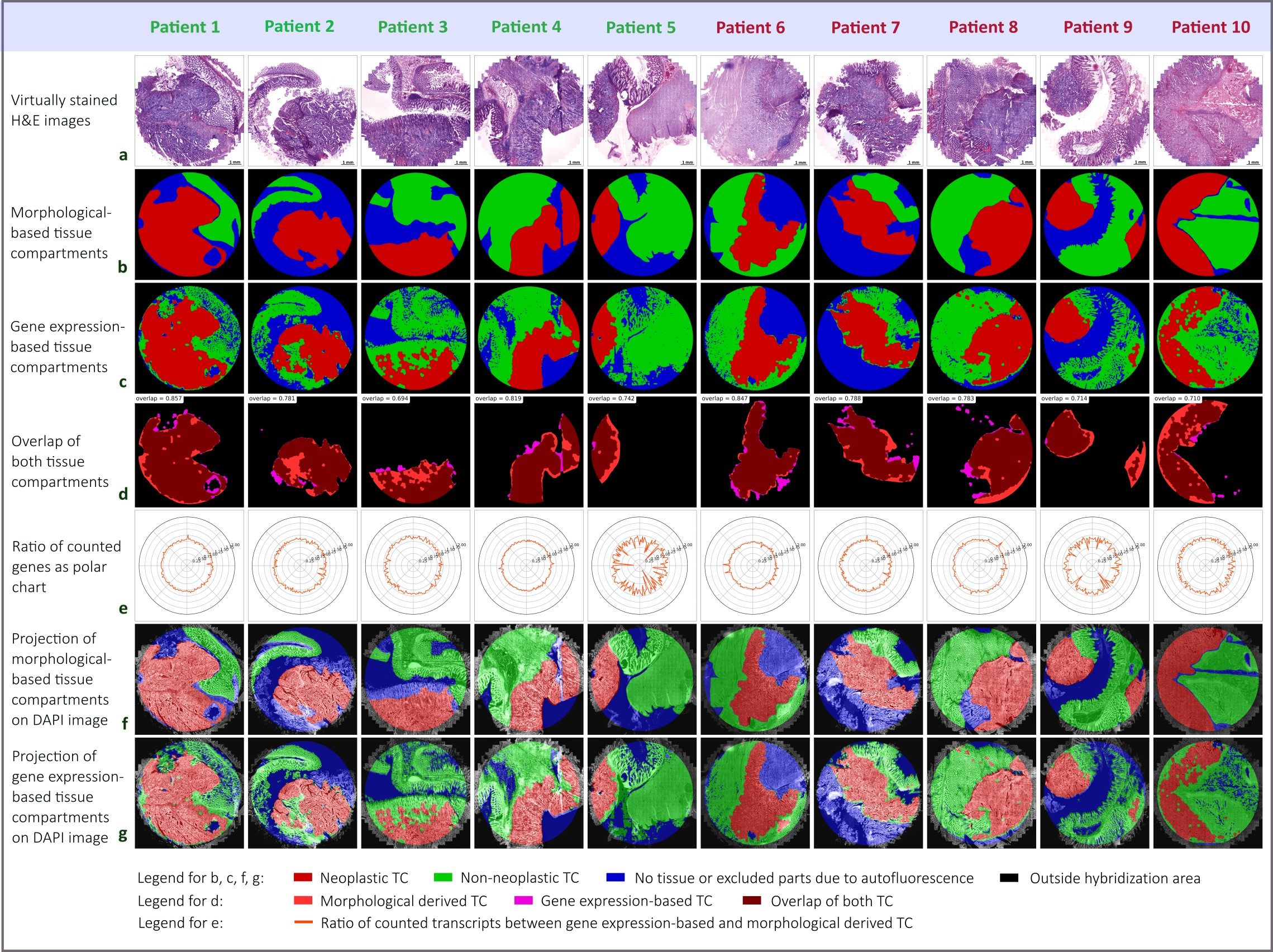
During the sequencing procedure, some parts of tissue areas detached from the slides while removing the coverslip, whereas others were damaged by accidently touching the tissue with the tip of the pipette. In Fig. S9 images of the same patient sample after different sequencing cycles can be seen, that clearly depict the issue of losing tissue.

**Cycle 4**

**Cycle 5**

H&E image

**Cycle 3**

e

a

d

c

b

***Fig. S9:*** Tissue damage (red) of patient sample 7 during the sequencing procedure. a) Virtually converted H&E staining of the sample. Image of tissue after b) the third c) the fourth and d) the fifth sequencing cycle. e) Shows the neoplastic and non-neoplastic compartments that were generated based on gene expression with the excluded area in blue. Tissue damage was observed after sequencing cycle 3-5. The red marked area had to be excluded from further analysis.

- High autofluorescence seen in patient sample 6


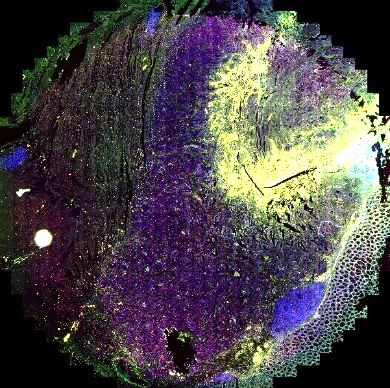

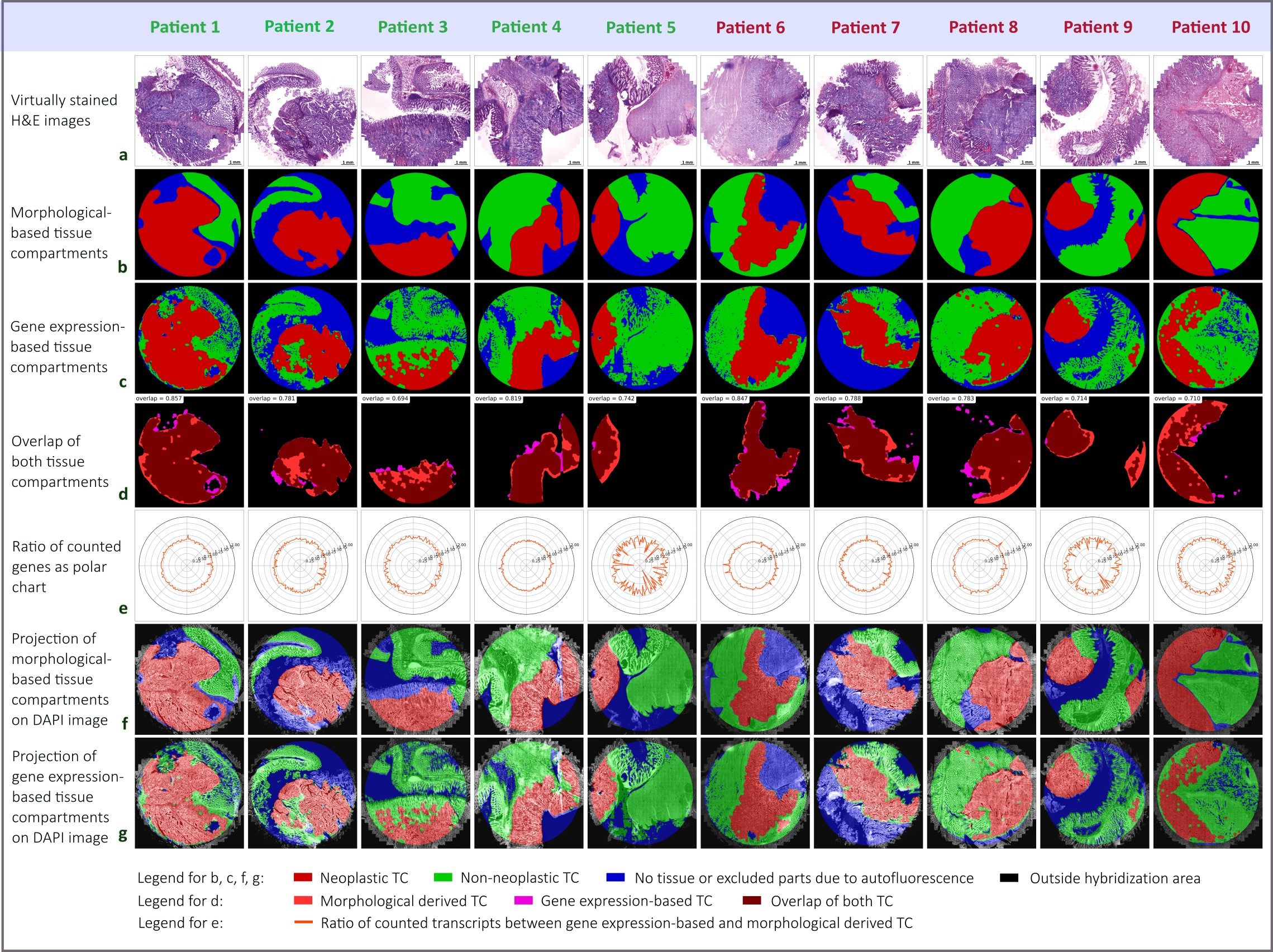

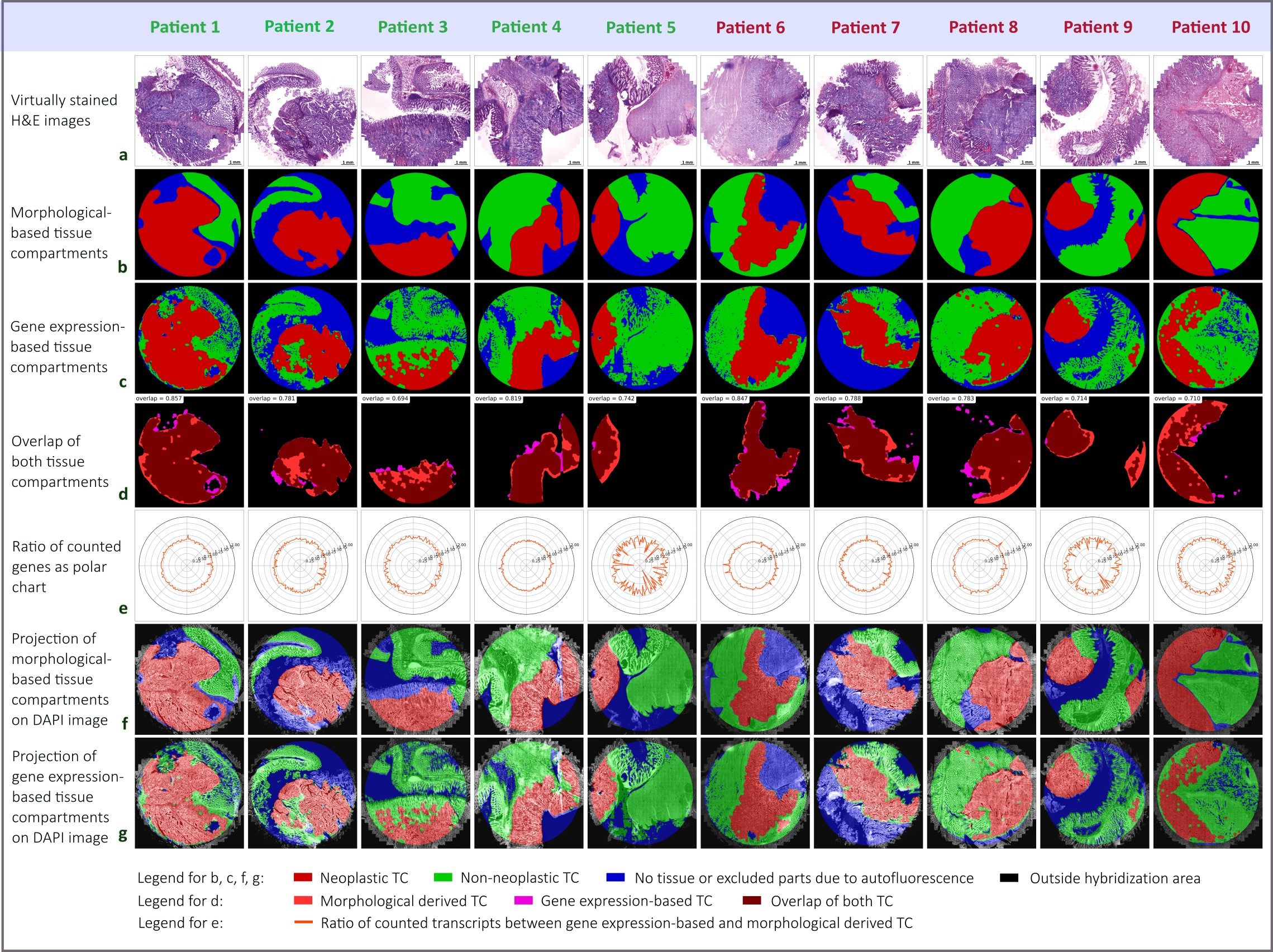
Some of the samples show highly fluorescent tissue structures that were excluded from analysis. The decision to exclude areas was made based on the fact that some autofluorescent areas showed pixel intensity values exceeding the maximum pixel intensity values of true positive signals. E.g.: if the maximum pixel intensities measured for Cy3 in one cycle was 2000, areas that exceeded this value were excluded from analysis.

a

b

c

***Fig. S10:*** Highly autofluorescent areas observed in patient sample 6 (red) that showed higher pixel intensity values than truly positive signals were excluded from further analysis. a) Virtually converted H&E staining of the sample. b) Highly autofluorescent region was marked in red. c) Shows the neoplastic and non-neoplastic compartments that were generated based on gene expression with the excluded area in blue.

- Low alignment of in situ signals

During the second alignment step performed in CellProfiler using the ImageJ plugins MultiStackReg, StackReg and TurboReg, some of the tiles showed low alignment. Fig. S11a shows a perfectly aligned tile whereas Fig. S11b depicts a wrongly aligned tile that needs to be excluded from further analysis.


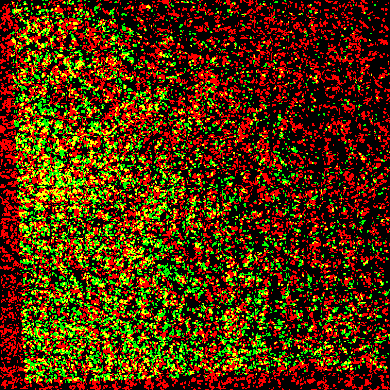

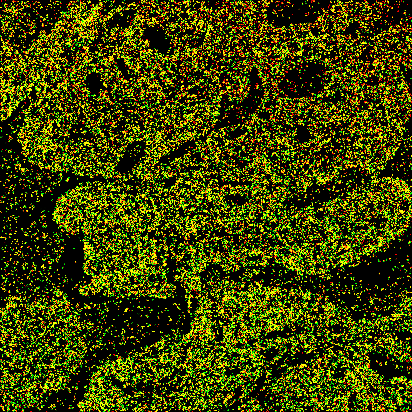


***Fig. S11:*** *Alignment of spots:* Alignment performed in CellProfiler by using ImageJ plugins MultiStackReg, StackReg and TurboReg. The figure shows a) correctly aligned spots and b) wrongly aligned spots.

1mm

b

a

**Method S7: Statistical testing**

A two-tailed paired t-test is used for the statistical significance testing of the transcript distribution in the neoplastic $D_{G}^{\mathrm{neoplastic}}$and the non-neoplastic $D_{G}^{non-neoplastic}$ tissues of the $N$ patients:

| $\begin{matrix} H_{0}: \mu_{d}=0 \\ H_{1}: \mu_{d}\neq0 \end{matrix}$ | (21) |
| --- | --- |

with $d_{n}=D_{G}^{\mathrm{neoplastic}}-D_{G}^{non-neoplastic}$ for $\alpha=0.05.$

Further, a two-tailed independent t-test is applied for statistical significance testing of the transcript distribution in the neoplastic tissue of the $N/2$ patients in the relapse group with the mean ${\mu_{\mathrm{relapse}}=\bar{D}}_{G,relapse}^{\mathrm{neoplastic}}$ and the non-relapse group with the mean $\mu_{no-relapse}=\bar{D}_{G,no-relapse}^{\mathrm{neoplastic}}$, yielding:

| $\begin{matrix} H_{0}: \mu_{\mathrm{relapse}}=\mu_{no-relapse} \\ H_{1}: \mu_{\mathrm{relapse}}\neq\mu_{no-relapse} \end{matrix}$ | (22) |
| --- | --- |

for $\alpha=0.05.$

The statistical testing is done for both the morphological as well as for the by gene expression-based TC using the implemented tests in the Python package scipy.

***Table S5:*** The resulting p-values for the statistical testing of relapse and non-relapse patients with the neoplastic tissue compartments.

| **genes** | **p-value** | **mean relapse patients** | **mean non-relapse patients** |
| --- | --- | --- | --- |
| ADGRA2 | 0.4380 | 0.0017 | 0.0013 |
| ALDH1A1 | 0.9271 | 0.0021 | 0.0019 |
| ANGPT1 | 0.9514 | 0.0010 | 0.0010 |
| ANGPT2 | 0.2625 | 0.0104 | 0.0040 |
| ANPEP | 0.5374 | 0.0021 | 0.0034 |
| ANTXR1 | 0.5995 | 0.0764 | 0.0673 |
| ATG5 | 0.4726 | 0.0041 | 0.0029 |
| AURKA | 0.7366 | 0.0092 | 0.0083 |
| BAK1 | 0.9219 | 0.0087 | 0.0084 |
| BBC3 | 0.8933 | 0.0893 | 0.0937 |
| BCL2L1 | 0.1268 | 0.0601 | 0.0360 |
| BCL2L11 | 0.7712 | 0.0039 | 0.0041 |
| BECN1 | 0.6856 | 0.0043 | 0.0036 |
| BEST4 | 0.3816 | 0.0058 | 0.0046 |
| BGN | 0.5344 | 0.0093 | 0.0065 |
| BID | 0.2140 | 0.0429 | 0.0200 |
| BIK | 0.4501 | 0.0260 | 0.0188 |
| BMI1 | 0.3415 | 0.0084 | 0.0020 |
| BOP1 | 0.8287 | 0.0327 | 0.0366 |
| BTG2 | 0.1062 | 0.0519 | 0.0287 |
| BTLA | 0.4055 | 0.0005 | 0.0006 |
| CA1 | 0.3870 | 0.0022 | 0.0017 |
| CA2 | 0.5645 | 0.0012 | 0.0017 |
| CASP3 | 0.0700 | 0.0100 | 0.0057 |
| CASP7 | 0.0682 | 0.0609 | 0.0210 |
| CASP8 | 0.5583 | 0.0034 | 0.0024 |
| CASP9 | 0.2590 | 0.0041 | 0.0028 |
| CAT | 0.8154 | 0.0054 | 0.0050 |
| CCND1 | 0.8206 | 0.0243 | 0.0207 |
| CCNE1 | 0.5620 | 0.0411 | 0.0279 |
| CD248 | 0.4689 | 0.0030 | 0.0037 |
| CD44 | 0.9015 | 0.0186 | 0.0196 |
| CD80 | 0.6438 | 0.0001 | 0.0001 |
| CD83 | 0.5719 | 0.0016 | 0.0018 |
| CD86 | 0.0738 | 0.0011 | 0.0006 |
| CDH5 | 0.2937 | 0.0026 | 0.0019 |
| CFLAR | 0.2040 | 0.0032 | 0.0008 |
| CHGA | 0.1433 | 0.0076 | 0.0038 |
| CNTD2 | 0.5664 | 0.0091 | 0.0124 |
| COL1A2 | 0.6891 | 0.1772 | 0.1424 |
| CSPG4 | 0.3571 | 0.0077 | 0.0057 |
| CXCL1 | 0.2984 | 0.0069 | 0.0042 |
| CXCL12 | 0.8857 | 0.0076 | 0.0073 |
| CXCL8 | 0.4133 | 0.0058 | 0.0039 |
| DCLK1 | 0.2134 | 0.0012 | 0.0008 |
| DCN | 0.7197 | 0.0016 | 0.0018 |
| E2F1 | 0.8938 | 0.0094 | 0.0088 |
| EGFR | 0.3622 | 0.0165 | 0.0109 |
| EGLN3 | 0.2134 | 0.0066 | 0.0125 |
| ENAH | 0.2609 | 0.0263 | 0.0400 |
| ENG | 0.6169 | 0.0630 | 0.0534 |
| ENTPD1 | 0.4883 | 0.0013 | 0.0049 |
| EPHB2 | 0.1347 | 0.0030 | 0.0017 |
| EREG | 0.2798 | 0.0085 | 0.0037 |
| EXOSC5 | 0.5477 | 0.0307 | 0.0241 |
| F8 | 0.5569 | 0.0041 | 0.0027 |
| FABP1 | 0.1063 | 0.3312 | 0.1101 |
| FABP4 | 0.2537 | 0.0015 | 0.0006 |
| FAM3C | 0.5082 | 0.0042 | 0.0033 |
| FGFR2 | 0.0137 | 0.0049 | 0.0031 |
| FLT4 | 0.3144 | 0.0252 | 0.0179 |
| FN1 | 0.2072 | 0.0830 | 0.0256 |
| FOXC2 | 0.1784 | 0.0009 | 0.0006 |
| FRK | 0.7101 | 0.0015 | 0.0014 |
| FSTL1 | 0.7359 | 0.0068 | 0.0063 |
| GADD45B | 0.3688 | 0.0076 | 0.0062 |
| GAST | 0.0856 | 0.0046 | 0.0032 |
| GFI1B | 0.4393 | 0.0023 | 0.0014 |
| GIP | 0.1603 | 0.0011 | 0.0007 |
| GLS | 0.5713 | 0.0131 | 0.0104 |
| GLUD1 | 0.6640 | 0.0028 | 0.0032 |
| GPX1 | 0.6236 | 0.0280 | 0.0229 |
| GRB7 | 0.2775 | 0.0115 | 0.0026 |
| GSR | 0.6764 | 0.0213 | 0.0176 |
| GUCA2B | 0.2908 | 0.0024 | 0.0012 |
| HIF1A | 0.7241 | 0.0043 | 0.0048 |
| HIF3A | 0.1704 | 0.0037 | 0.0020 |
| HK1 | 0.4892 | 0.0145 | 0.0115 |
| HK2 | 0.5087 | 0.0157 | 0.0117 |
| HMGB1 | 0.3301 | 0.0080 | 0.0023 |
| HYOU1 | 0.4569 | 0.0457 | 0.0344 |
| ICAM1 | 0.7122 | 0.0051 | 0.0044 |
| IDO2 | 0.1770 | 0.0009 | 0.0004 |
| IGFBP2 | 0.7922 | 0.0483 | 0.0441 |
| IL11 | 0.3020 | 0.0034 | 0.0027 |
| IL1B | 0.1749 | 0.0265 | 0.0187 |
| IL6 | 0.3527 | 0.0019 | 0.0009 |
| IL7R | 0.4805 | 0.0017 | 0.0015 |
| INHBA | 0.9982 | 0.0038 | 0.0038 |
| ITGAM | 0.8851 | 0.0022 | 0.0021 |
| ITGAV | 0.6371 | 0.0158 | 0.0126 |
| ITGAX | 0.3278 | 0.0010 | 0.0005 |
| KDR | 0.7293 | 0.0027 | 0.0024 |
| KIT | 0.4801 | 0.0010 | 0.0005 |
| KLF4 | 0.1445 | 0.2326 | 0.0777 |
| KLRK1 | 0.8630 | 0.0007 | 0.0007 |
| L1CAM | 0.1590 | 0.0040 | 0.0031 |
| LAMC2 | 0.5822 | 0.0051 | 0.0078 |
| LDHA | 0.1784 | 0.0052 | 0.0081 |
| LGR5 | 0.7745 | 0.0043 | 0.0038 |
| LIF | 0.3691 | 0.0055 | 0.0034 |
| MAP1LC3A | 0.6020 | 0.0214 | 0.0168 |
| MCL1 | 0.3200 | 0.0178 | 0.0114 |
| MCM2 | 0.4943 | 0.0118 | 0.0084 |
| MET | 0.4367 | 0.0327 | 0.0499 |
| MICA | 0.4789 | 0.0023 | 0.0030 |
| MICB | 0.2659 | 0.0157 | 0.0062 |
| MIEN1 | 0.1606 | 0.0099 | 0.0029 |
| MLKL | 0.9694 | 0.0375 | 0.0370 |
| MLN | 0.2325 | 0.0053 | 0.0019 |
| MMP11 | 0.0415 | 0.0492 | 0.0164 |
| MMP2 | 0.8319 | 0.0019 | 0.0021 |
| MMP7 | 0.8832 | 0.0061 | 0.0068 |
| MPL | 0.1839 | 0.0010 | 0.0006 |
| MUC2 | 0.2674 | 0.0124 | 0.0080 |
| MUC5AC | 0.2946 | 0.0008 | 0.0005 |
| MYBL2 | 0.4202 | 0.0091 | 0.0057 |
| NANOG | 0.2675 | 0.0042 | 0.0024 |
| NCAM1 | 0.6225 | 0.0117 | 0.0106 |
| NCR1 | 0.7844 | 0.0010 | 0.0010 |
| NOS1 | 0.4461 | 0.0129 | 0.0102 |
| NOS2 | 0.6146 | 0.0076 | 0.0109 |
| NT5E | 0.8994 | 0.0027 | 0.0025 |
| NTS | 0.3922 | 0.0013 | 0.0008 |
| OLFM4 | 0.6367 | 0.0911 | 0.1395 |
| OSER1 | 0.4527 | 0.0102 | 0.0069 |
| OTOP2 | 0.0043 | 0.0026 | 0.0019 |
| PCNA | 0.5341 | 0.0125 | 0.0077 |
| PDGFA | 0.1237 | 0.0302 | 0.0157 |
| PDK1 | 0.4882 | 0.0024 | 0.0032 |
| PKM | 0.7688 | 0.0922 | 0.0817 |
| PLXDC1 | 0.5181 | 0.0062 | 0.0051 |
| POU2F3 | 0.5070 | 0.1033 | 0.0636 |
| POU5F1 | 0.5592 | 0.5020 | 0.3878 |
| PPIF | 0.3442 | 0.0056 | 0.0035 |
| PRDX2 | 0.7580 | 0.1062 | 0.0872 |
| PRF1 | 0.2779 | 0.0005 | 0.0011 |
| PROM1 | 0.0792 | 0.0086 | 0.0026 |
| PYY | 0.3809 | 0.0027 | 0.0019 |
| RIPK1 | 0.7866 | 0.0047 | 0.0051 |
| RIPK3 | 0.2560 | 0.0153 | 0.0115 |
| RPS6KB1 | 0.5633 | 0.0004 | 0.0008 |
| S100A4 | 0.0645 | 0.0308 | 0.0118 |
| SAAL1 | 0.3395 | 0.0018 | 0.0011 |
| SALL4 | 0.6568 | 0.0019 | 0.0023 |
| SCAI | 0.1913 | 0.0047 | 0.0026 |
| SLC26A3 | 0.9402 | 0.1045 | 0.1083 |
| SLC2A1 | 0.3493 | 0.0006 | 0.0004 |
| SOD1 | 0.9646 | 0.0983 | 0.0971 |
| SOX2 | 0.8878 | 0.0138 | 0.0131 |
| SPDEF | 0.2998 | 0.0039 | 0.0023 |
| SST | 0.5133 | 0.0011 | 0.0007 |
| TAGLN | 0.1000 | 0.0039 | 0.0015 |
| TBXT | 0.4052 | 0.0027 | 0.0012 |
| TEK | 0.9708 | 0.0005 | 0.0005 |
| TFF1 | 0.5438 | 0.0092 | 0.0134 |
| TFF3 | 0.4431 | 0.0703 | 0.0396 |
| TGFA | 0.1879 | 0.0057 | 0.0034 |
| TGFB3 | 0.9421 | 0.0032 | 0.0033 |
| THY1 | 0.6415 | 0.0036 | 0.0030 |
| TIAM1 | 0.2424 | 0.0009 | 0.0006 |
| TIE1 | 0.2877 | 0.0013 | 0.0010 |
| TIMP1 | 0.9941 | 0.0257 | 0.0256 |
| TJP1 | 0.4679 | 0.0133 | 0.0079 |
| TNC | 0.8990 | 0.0073 | 0.0068 |
| TNF | 0.5561 | 0.0010 | 0.0006 |
| TNFRSF10A | 0.4299 | 0.0071 | 0.0035 |
| TNFRSF10B | 0.3869 | 0.0028 | 0.0041 |
| TNFSF10 | 0.8549 | 0.0077 | 0.0071 |
| TRPM5 | 0.3110 | 0.0058 | 0.0025 |
| TWIST1 | 0.5346 | 0.0256 | 0.0212 |
| TXNL1 | 0.3908 | 0.0069 | 0.0049 |
| URGCP | 0.5626 | 0.0002 | 0.0004 |
| VCAM1 | 0.9583 | 0.0007 | 0.0007 |
| ZEB1 | 0.2654 | 0.0014 | 0.0007 |
| ZGLP1 | 0.3593 | 0.0026 | 0.0046 |

***Fig. S12:*** *Comparison of spatial analysis vs. bulk analysis:* Volcano plots with a significance level α=0.05 of significantly upregulated genes in a) the neoplastic tissues compartment and b) the total tissue area of relapsed patients in comparison to non-relapsed patients.

**b**

**a**

c


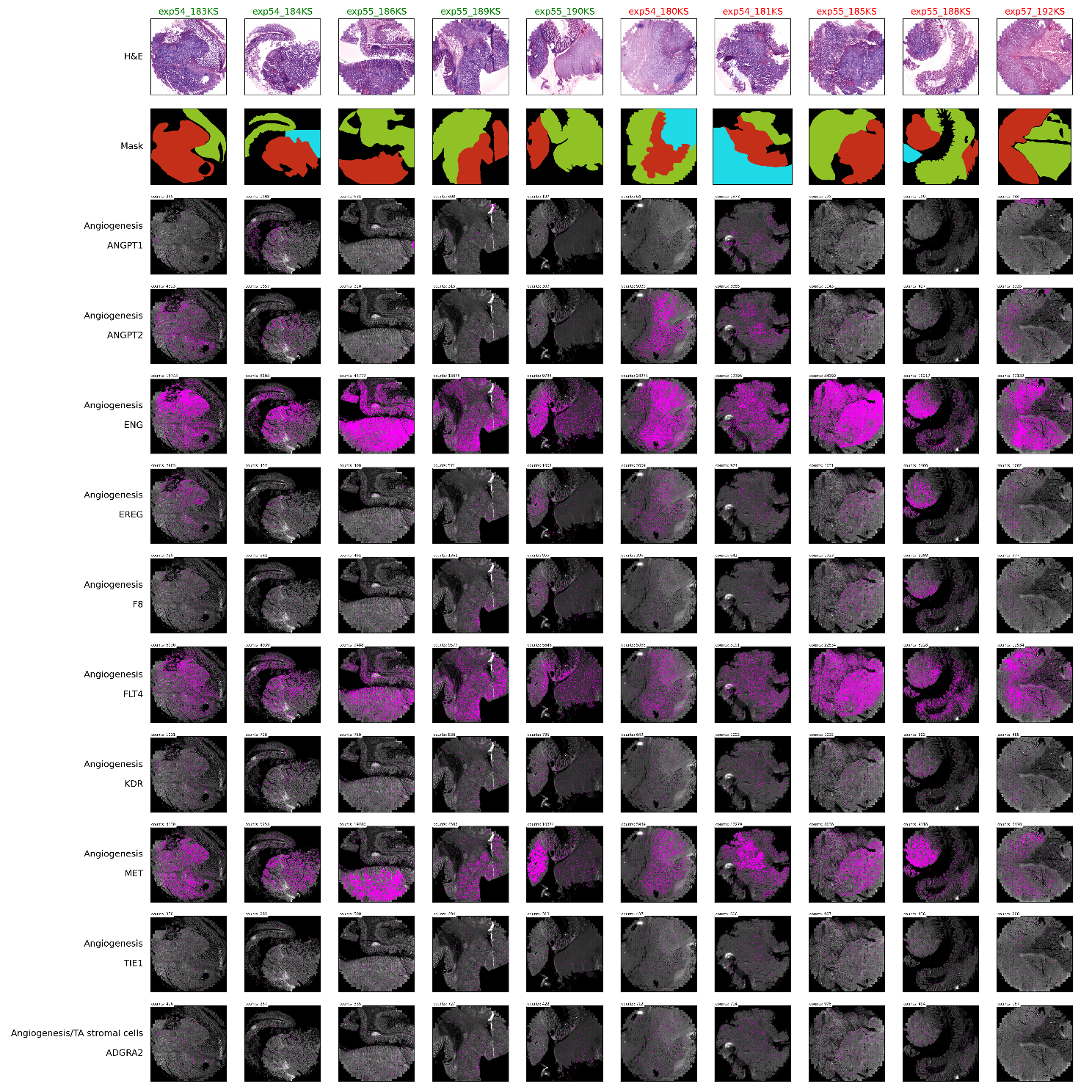


**a**

**Patient 1 Patient 2 Patient 3 Patient 4 Patient 5 Patient 6 Patient 7 Patient 8 Patient 9 Patient 10**

c


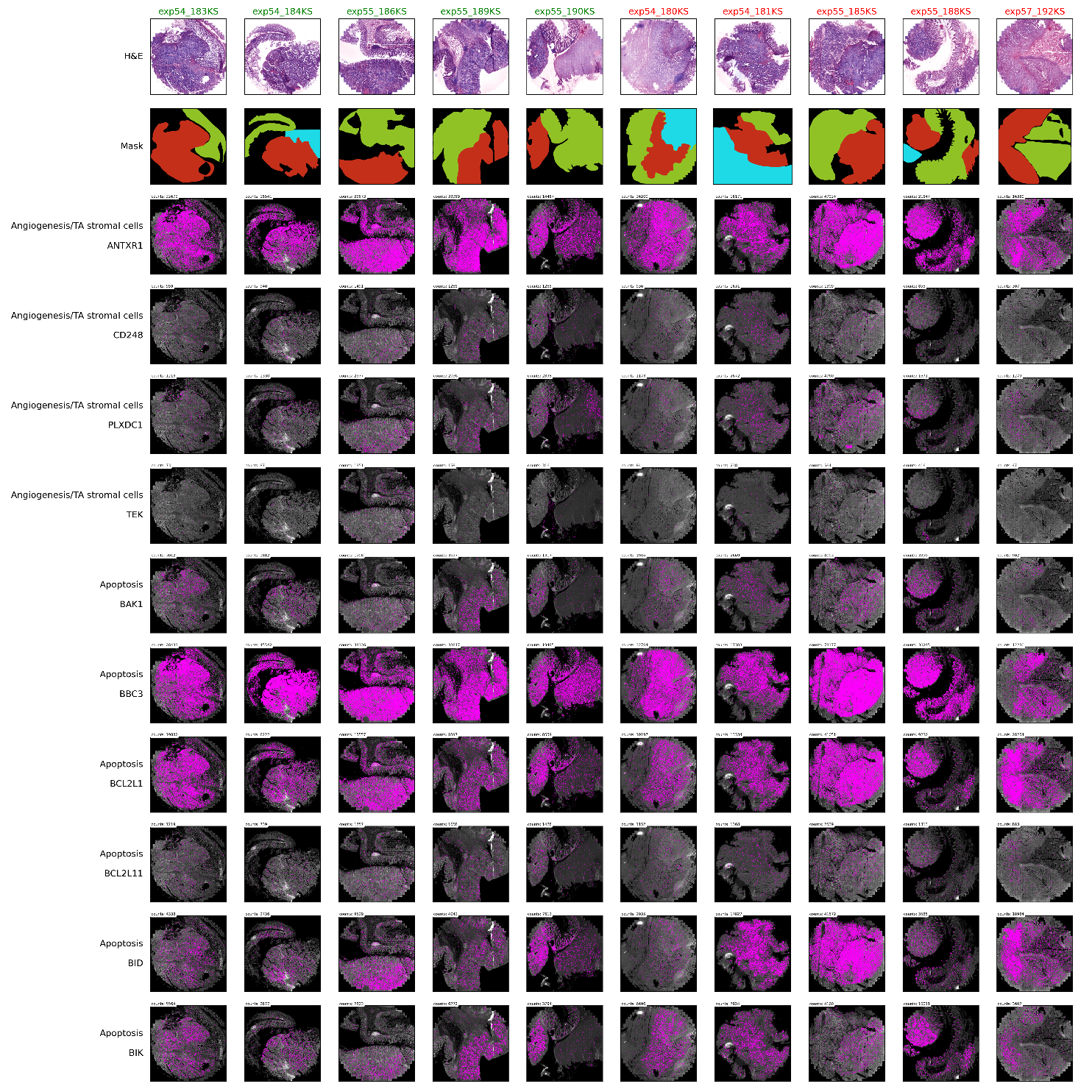


**b**

**Patient 1 Patient 2 Patient 3 Patient 4 Patient 5 Patient 6 Patient 7 Patient 8 Patient 9 Patient 10**


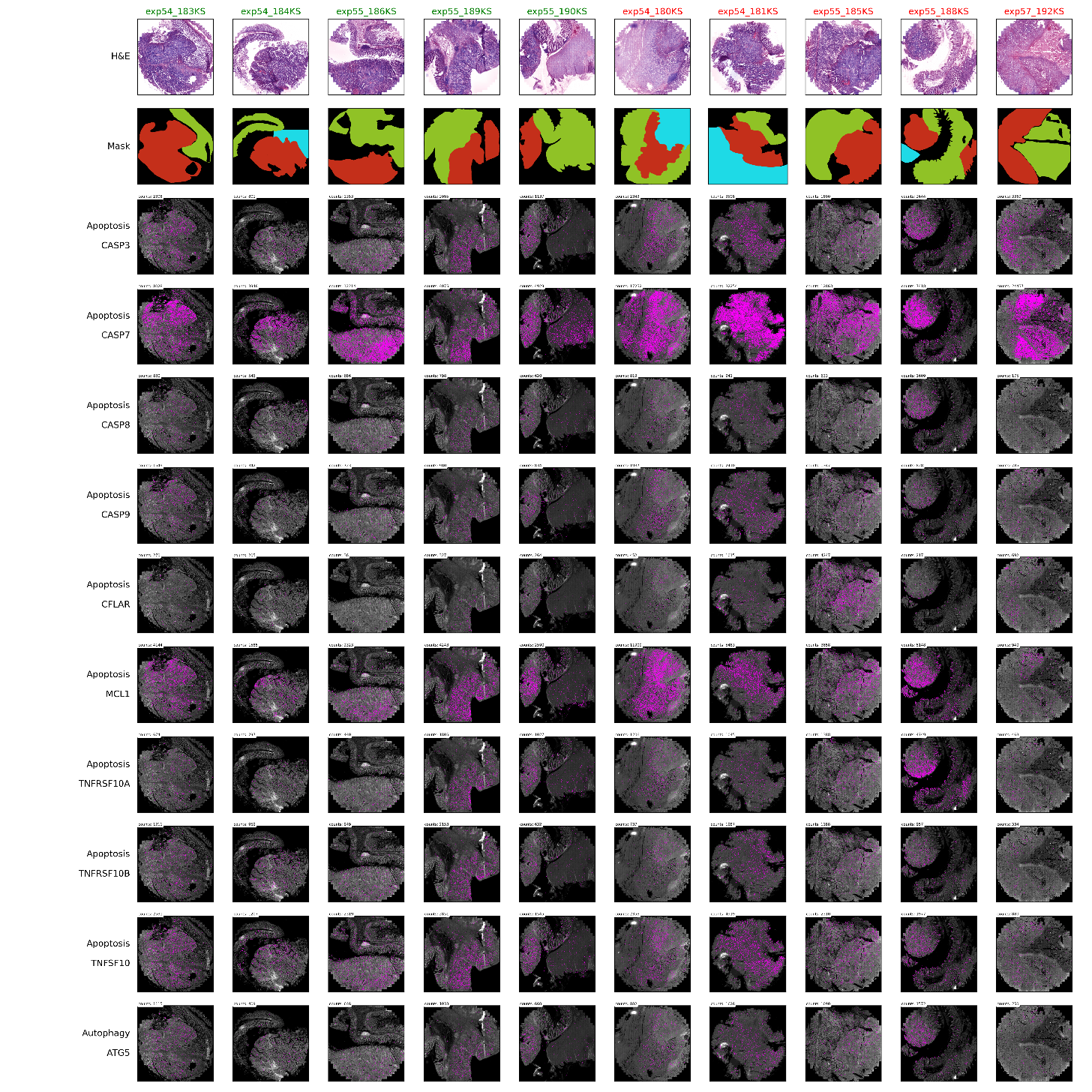


**c**

**Patient 1 Patient 2 Patient 3 Patient 4 Patient 5 Patient 6 Patient 7 Patient 8 Patient 9 Patient 10**

**d**


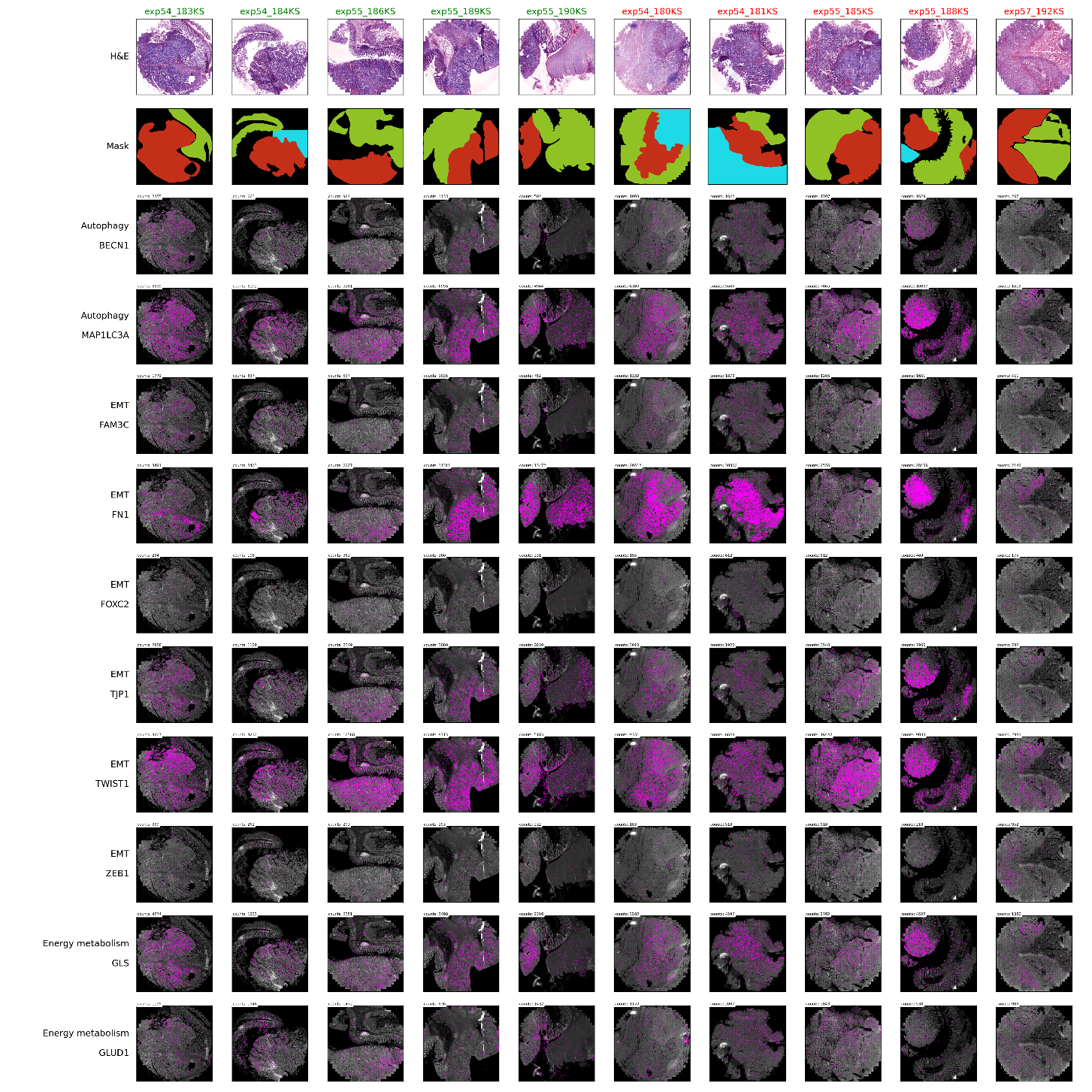


**d**

**Patient 1 Patient 2 Patient 3 Patient 4 Patient 5 Patient 6 Patient 7 Patient 8 Patient 9 Patient 10**


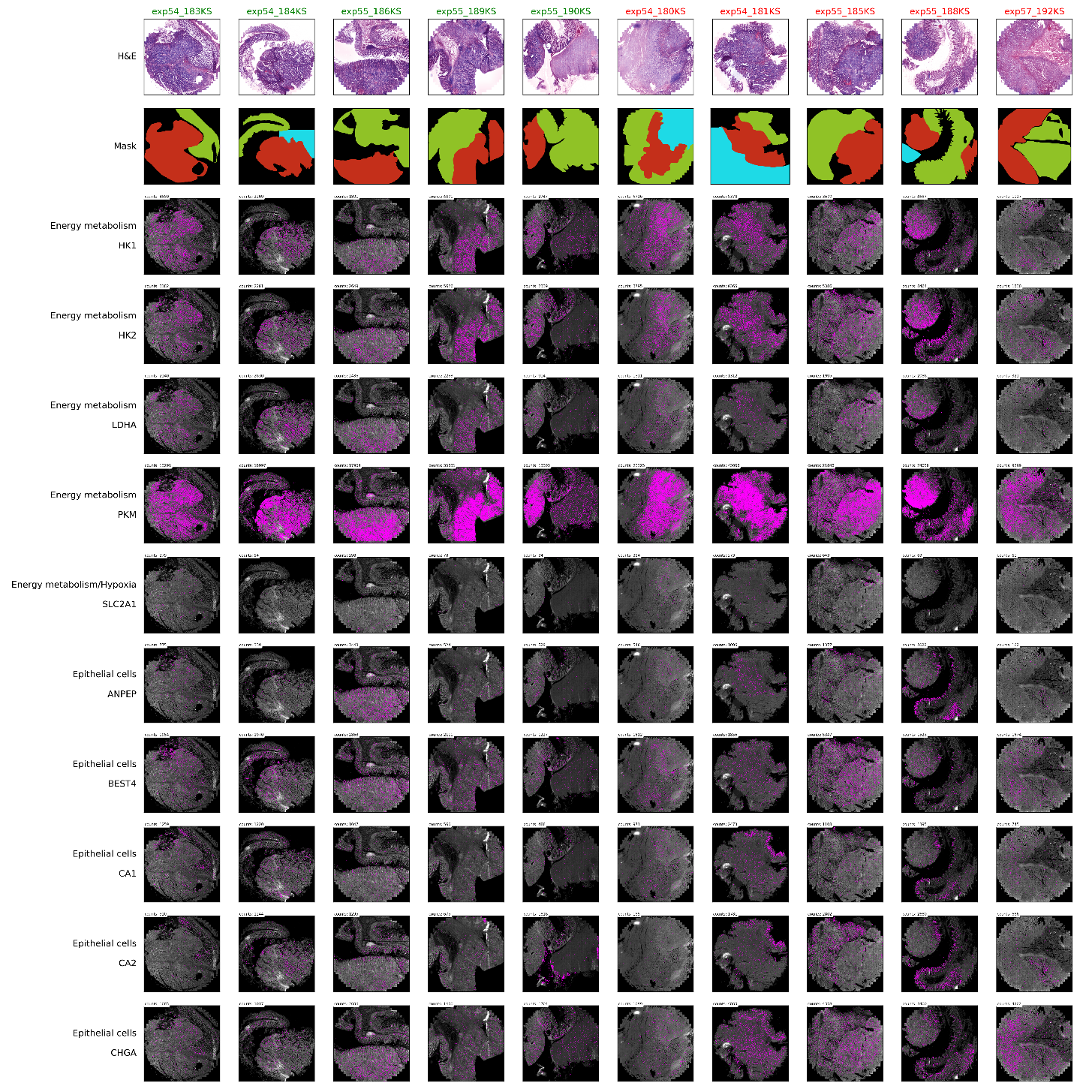


**e**

**Patient 1 Patient 2 Patient 3 Patient 4 Patient 5 Patient 6 Patient 7 Patient 8 Patient 9 Patient 10**


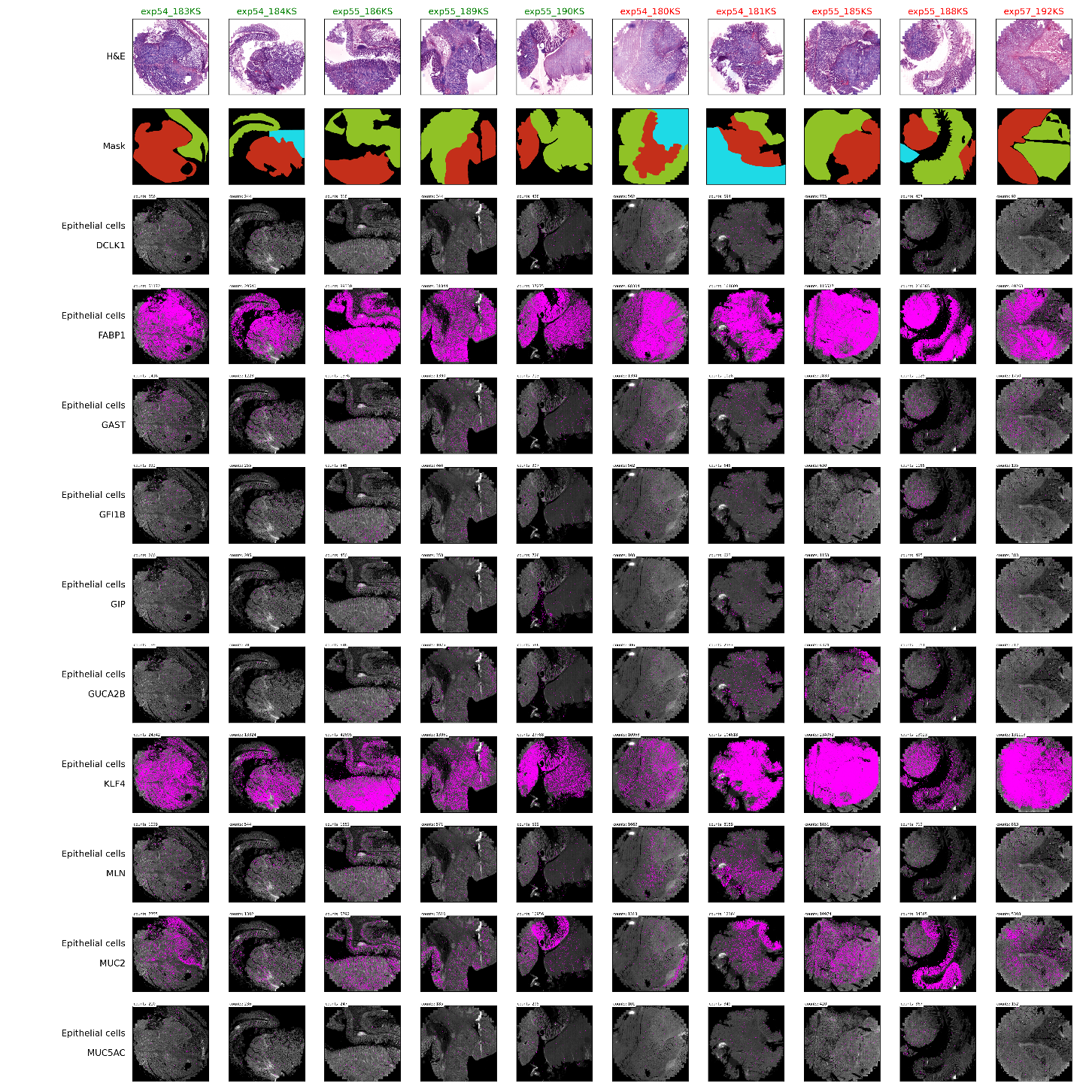


**f**

**Patient 1 Patient 2 Patient 3 Patient 4 Patient 5 Patient 6 Patient 7 Patient 8 Patient 9 Patient 10**


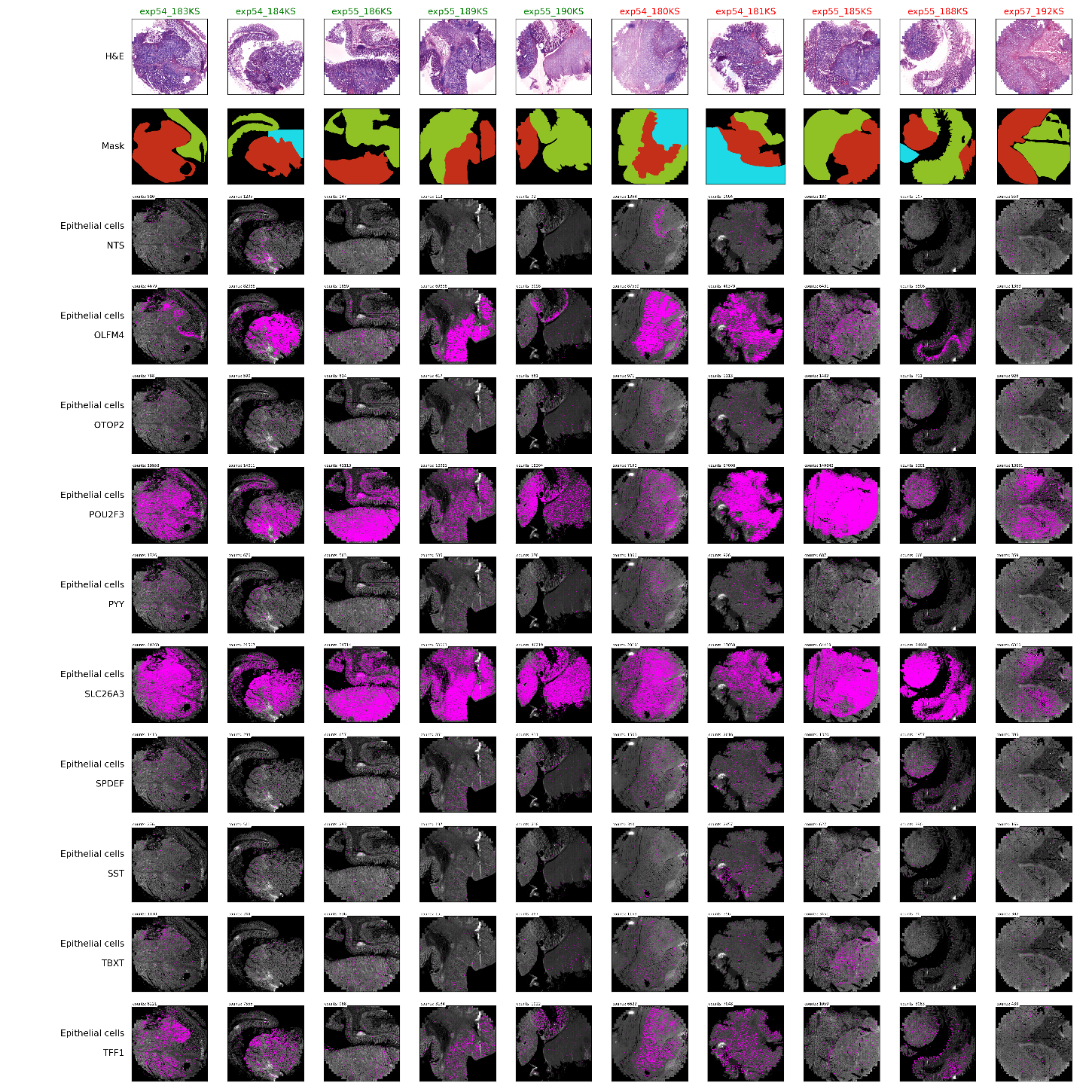


**g**

**Patient 1 Patient 2 Patient 3 Patient 4 Patient 5 Patient 6 Patient 7 Patient 8 Patient 9 Patient 10**


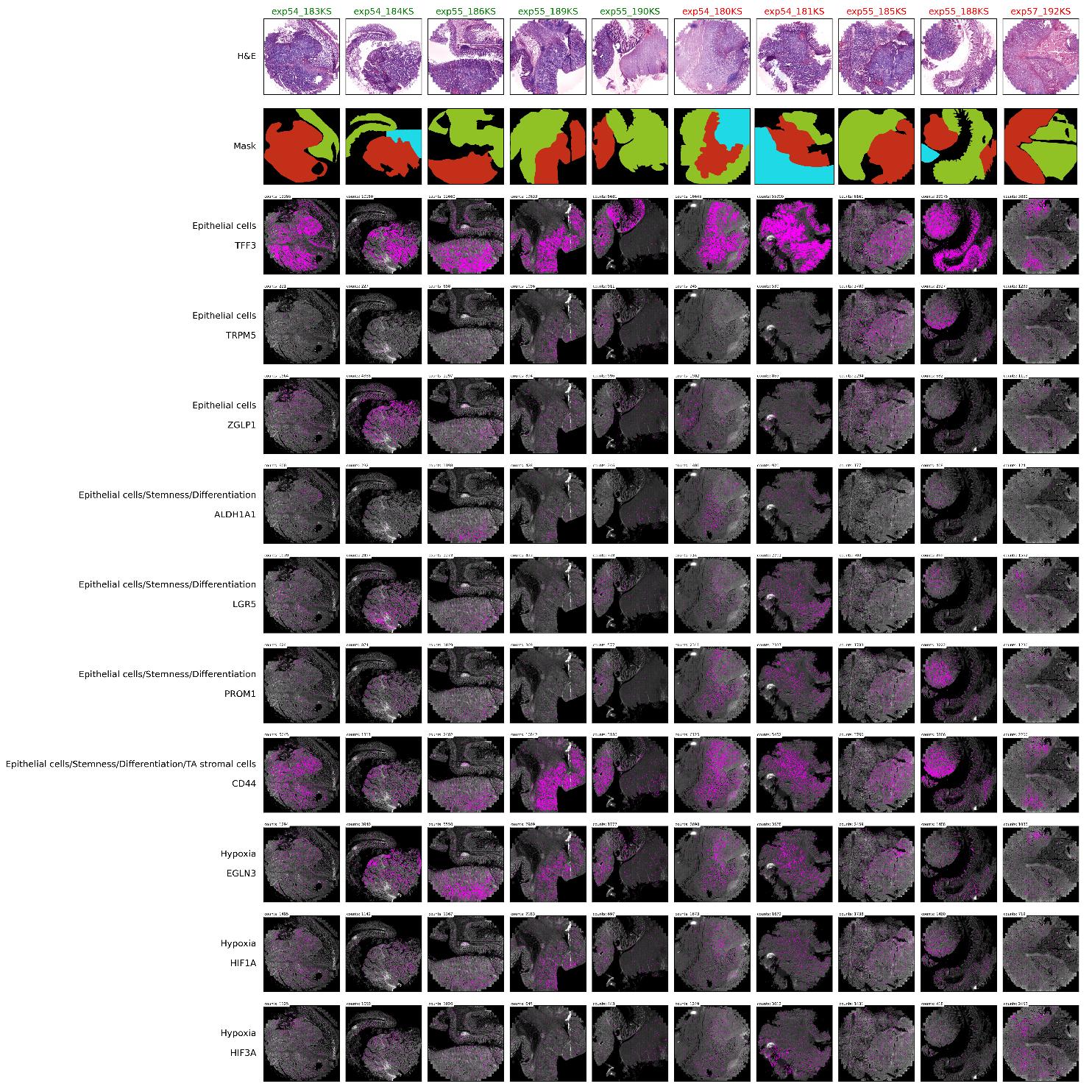


**h**

**Patient 1 Patient 2 Patient 3 Patient 4 Patient 5 Patient 6 Patient 7 Patient 8 Patient 9 Patient 10**

**i**


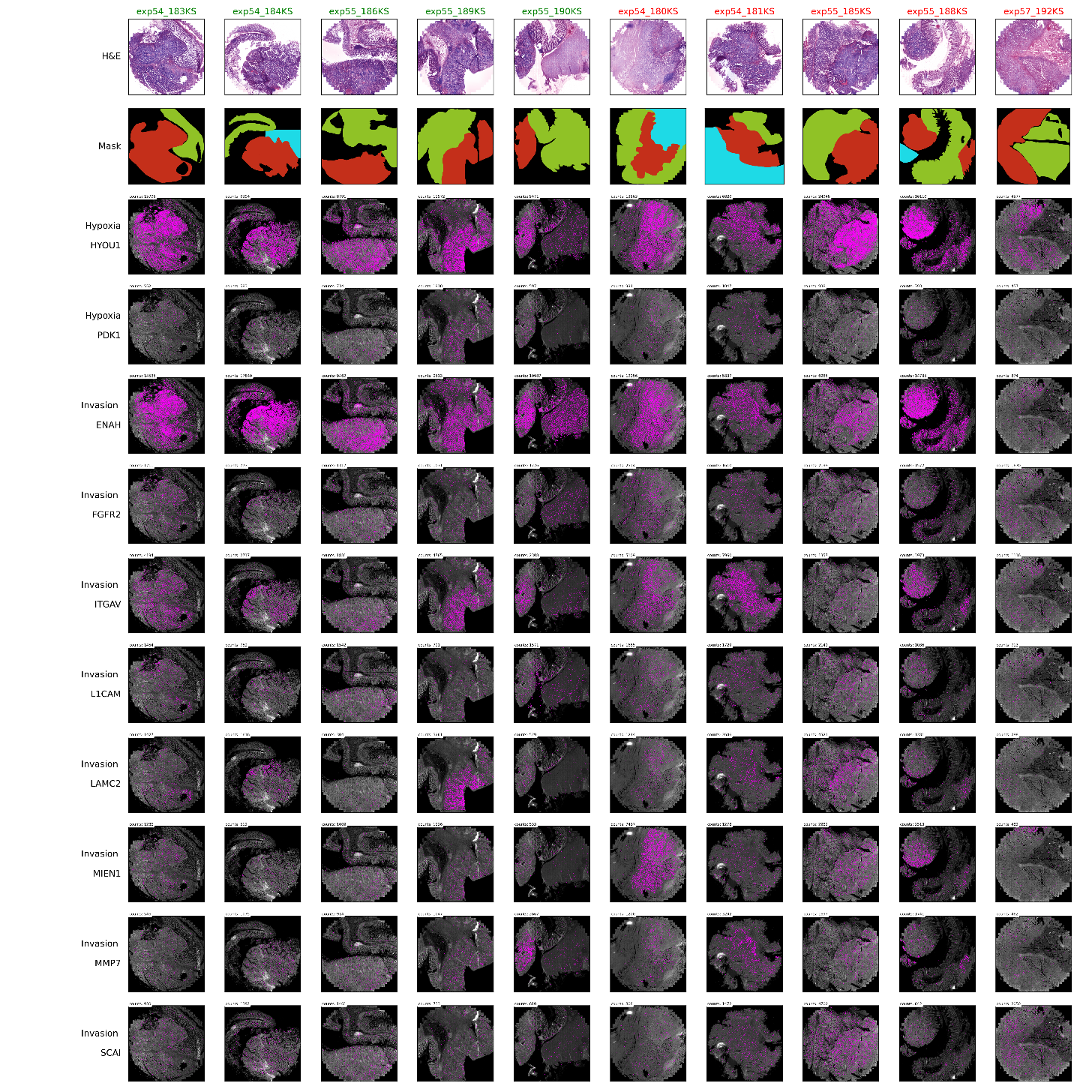


**i**

**Patient 1 Patient 2 Patient 3 Patient 4 Patient 5 Patient 6 Patient 7 Patient 8 Patient 9 Patient 10**

**j**


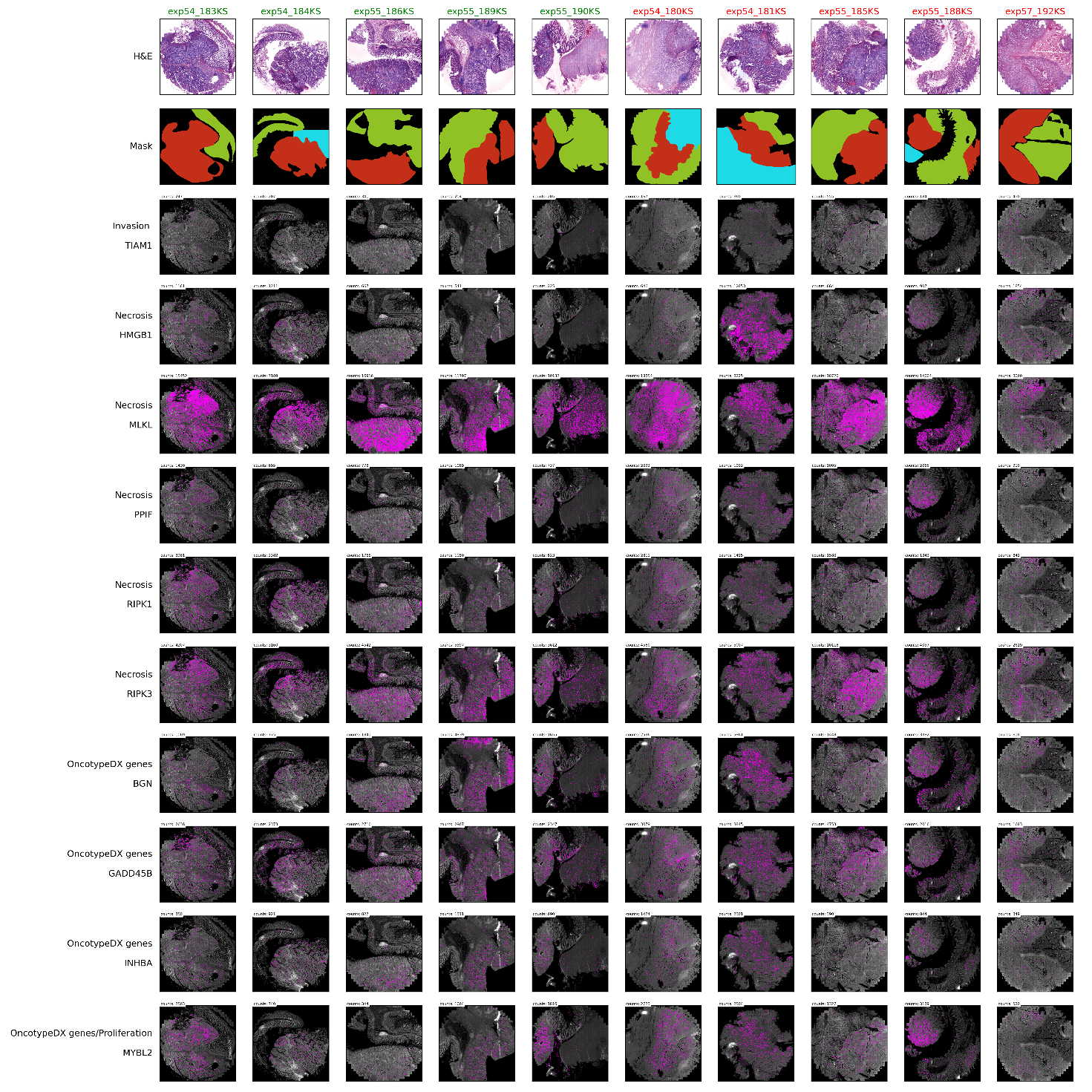


**j**

**Patient 1 Patient 2 Patient 3 Patient 4 Patient 5 Patient 6 Patient 7 Patient 8 Patient 9 Patient 10**


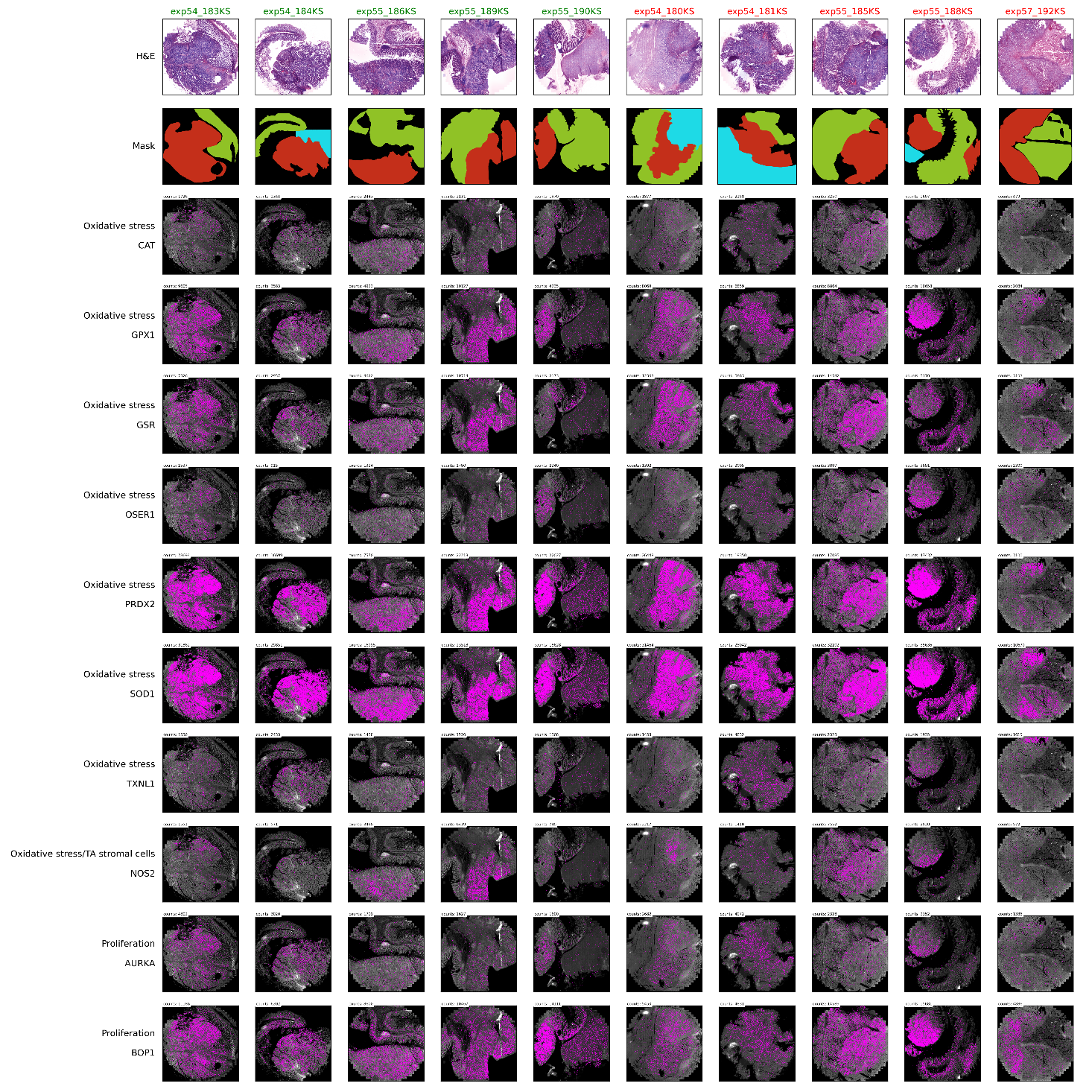


**k**

**Patient 1 Patient 2 Patient 3 Patient 4 Patient 5 Patient 6 Patient 7 Patient 8 Patient 9 Patient 10**


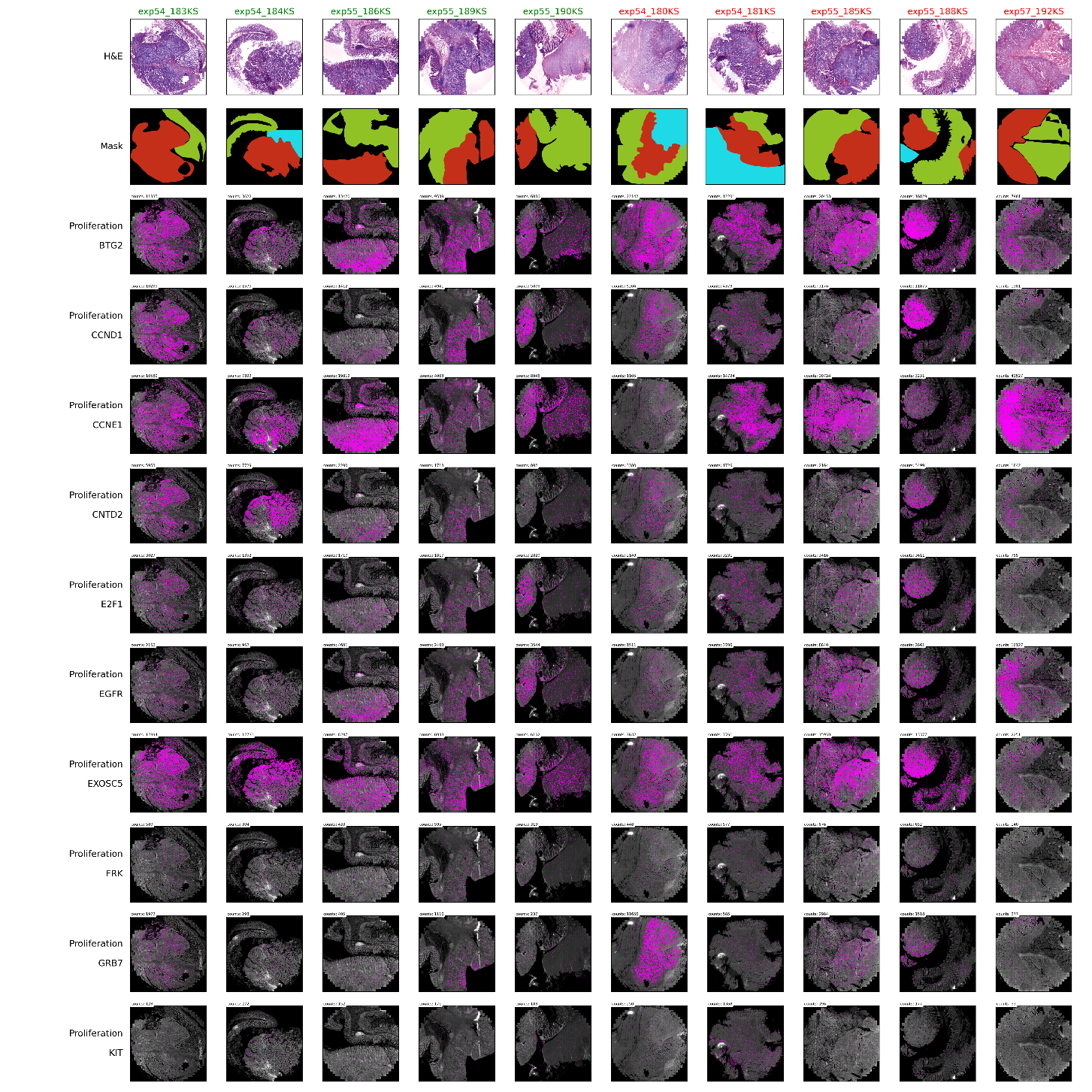


**l**

**Patient 1 Patient 2 Patient 3 Patient 4 Patient 5 Patient 6 Patient 7 Patient 8 Patient 9 Patient 10**


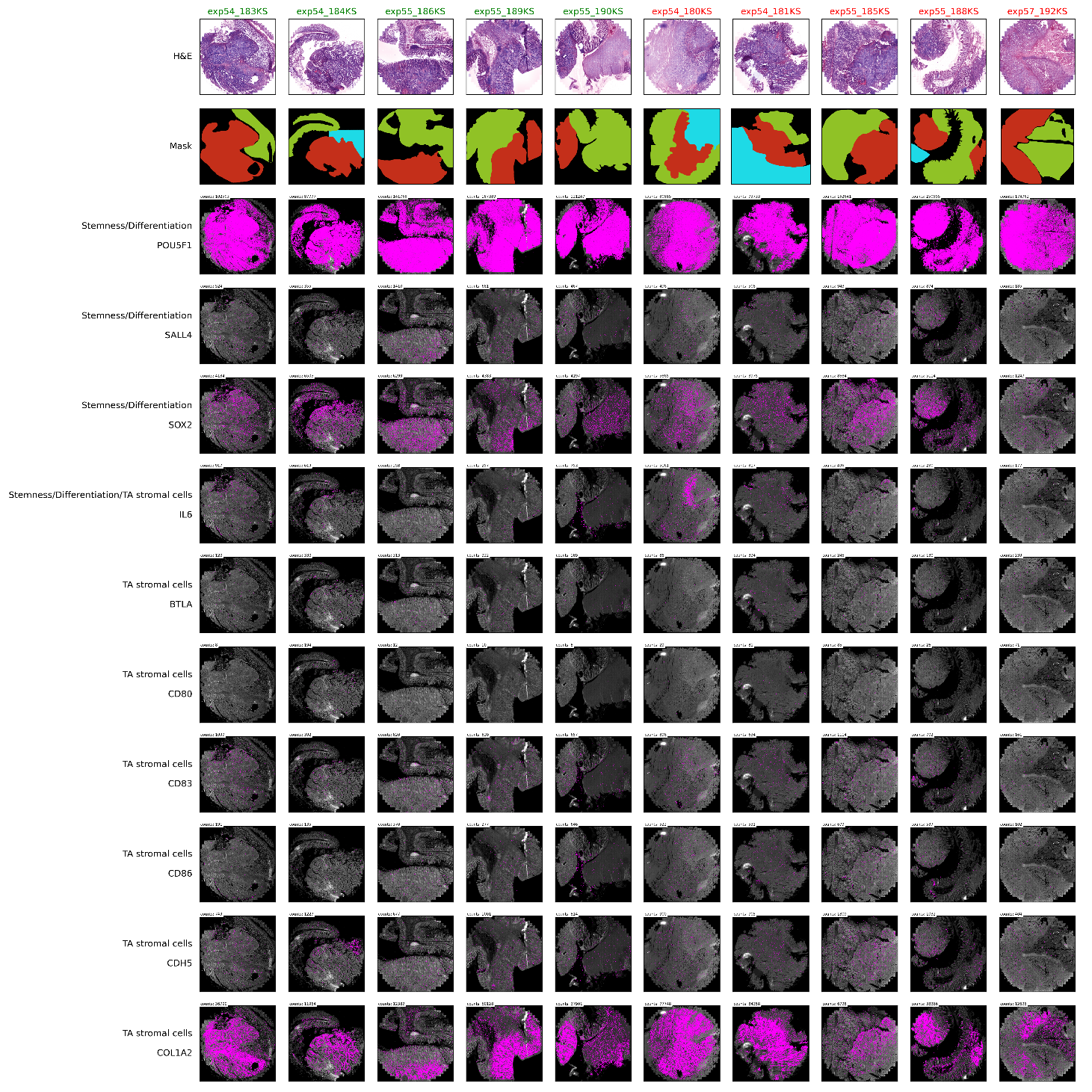


**m**

**Patient 1 Patient 2 Patient 3 Patient 4 Patient 5 Patient 6 Patient 7 Patient 8 Patient 9 Patient 10**


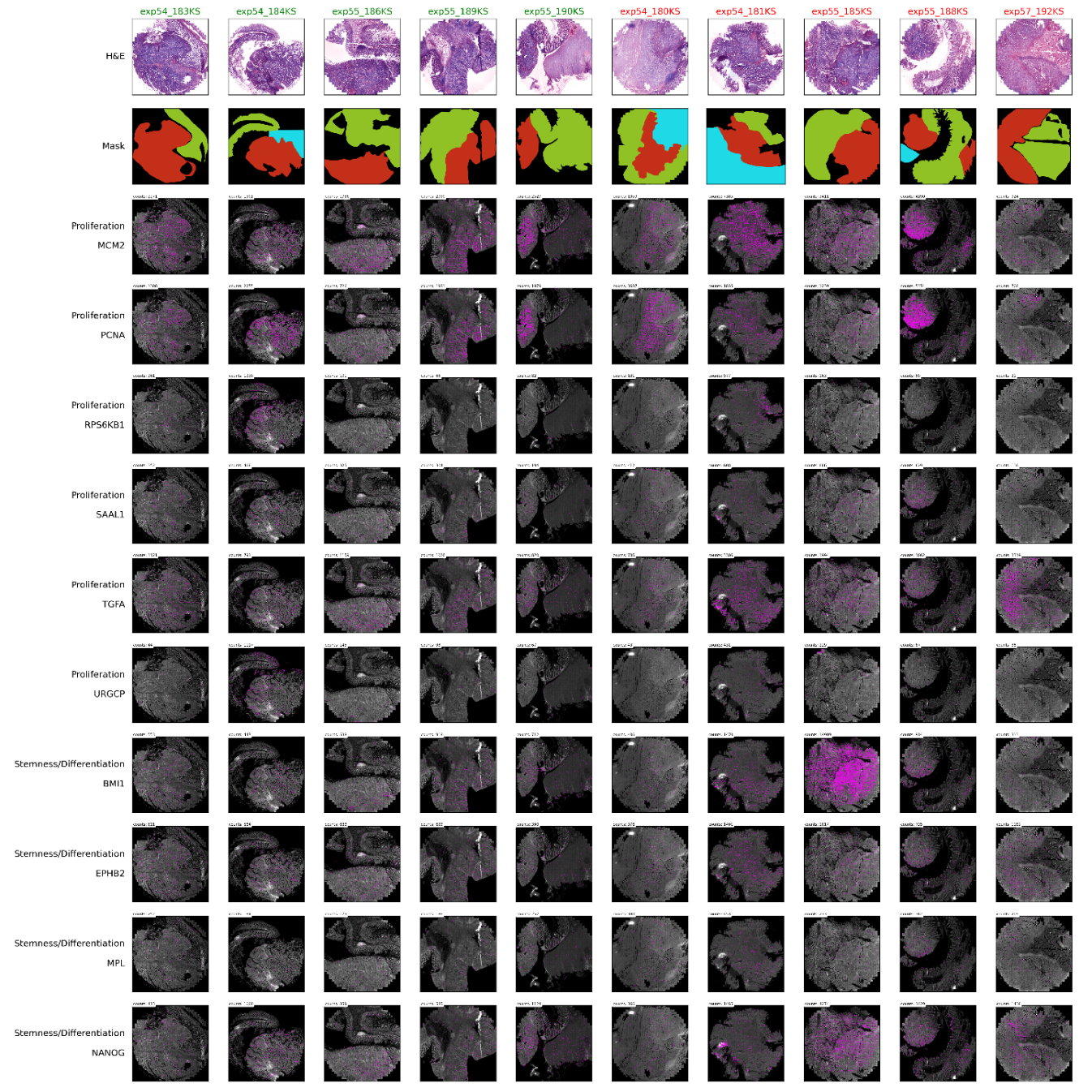


**n**

**Patient 1 Patient 2 Patient 3 Patient 4 Patient 5 Patient 6 Patient 7 Patient 8 Patient 9 Patient 10**


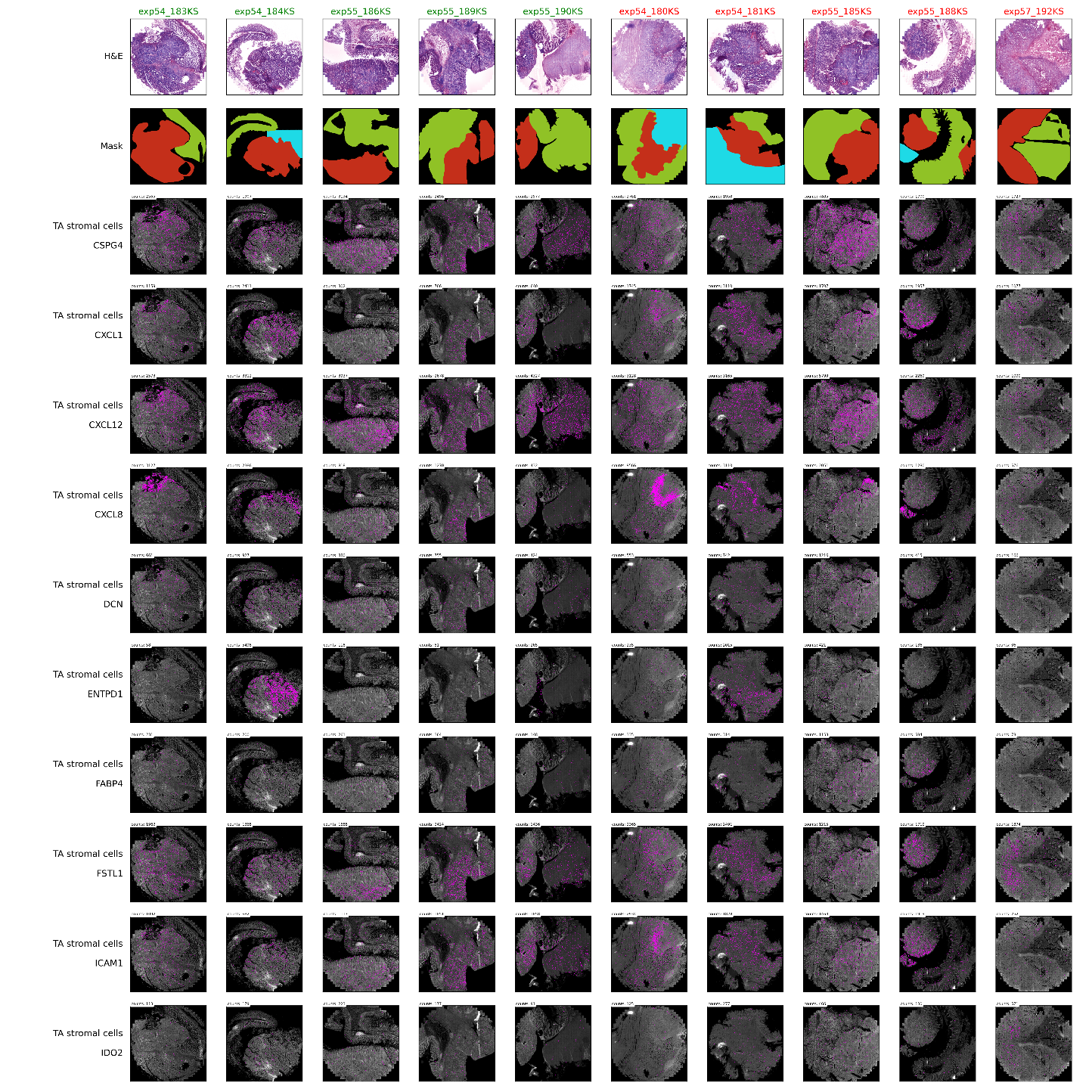


**o**

**Patient 1 Patient 2 Patient 3 Patient 4 Patient 5 Patient 6 Patient 7 Patient 8 Patient 9 Patient 10**


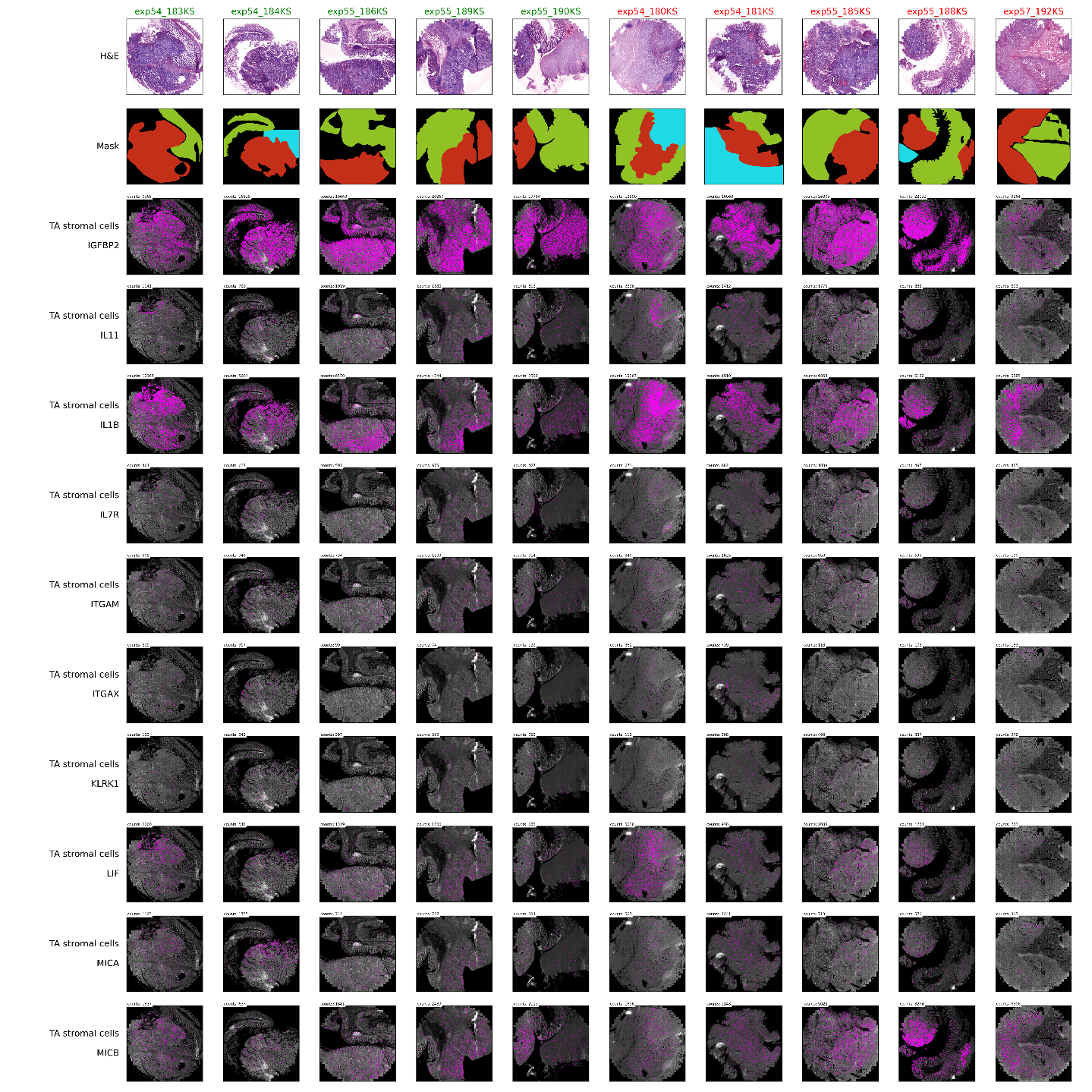


**p**

**Patient 1 Patient 2 Patient 3 Patient 4 Patient 5 Patient 6 Patient 7 Patient 8 Patient 9 Patient 10**


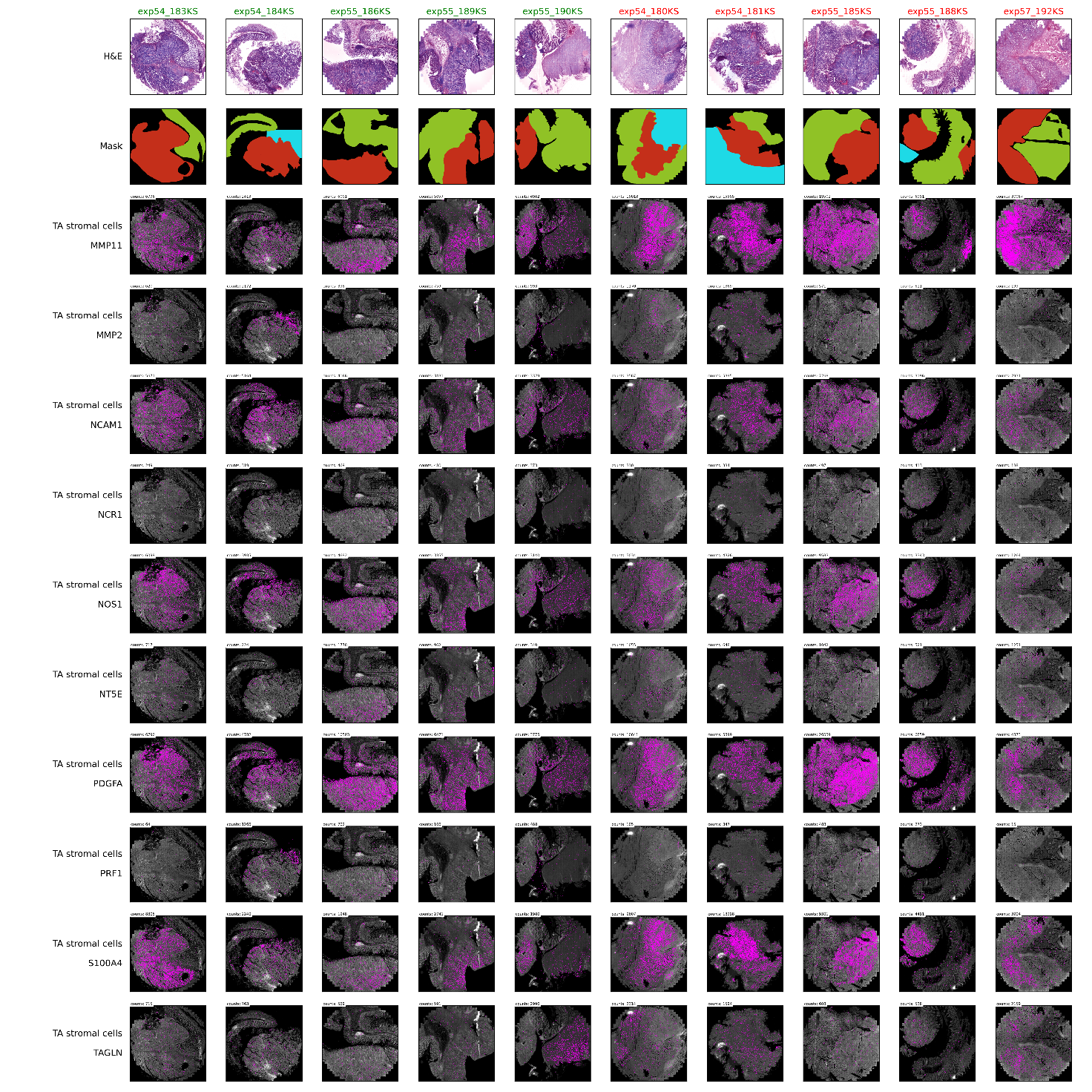


**q**

**Patient 1 Patient 2 Patient 3 Patient 4 Patient 5 Patient 6 Patient 7 Patient 8 Patient 9 Patient 10**


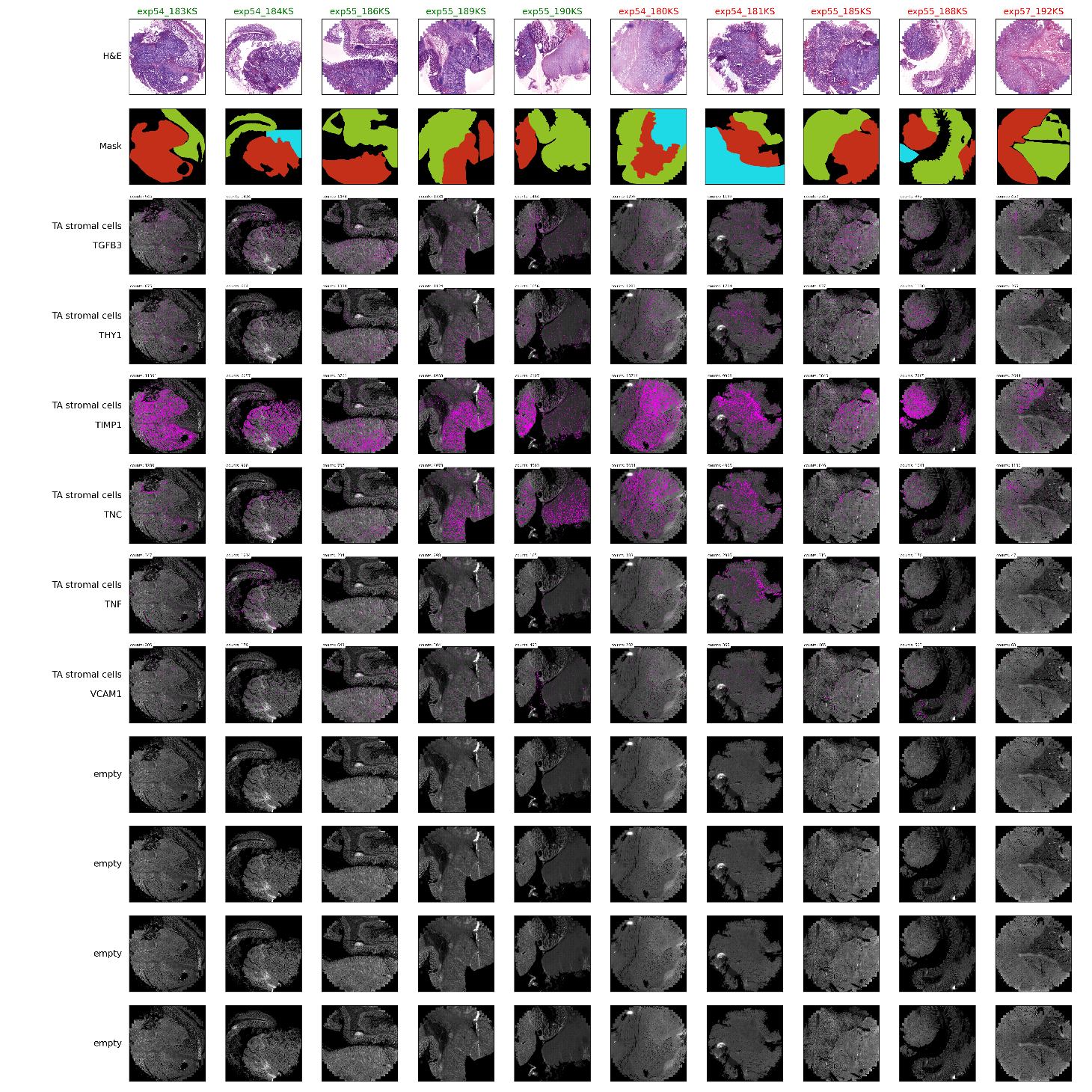


**r**

***Fig. S13:*** *Spatial distribution of 176 ISS detected transcripts.*

**Patient 1 Patient 2 Patient 3 Patient 4 Patient 5 Patient 6 Patient 7 Patient 8 Patient 9 Patient 10**

**Literature citations**

1. Uhlén M, Fagerberg L, Hallström BM, Lindskog C, Oksvold P, Mardinoglu A, Sivertsson Å, Kampf C, Sjöstedt E, Asplund A, Olsson I, Edlund K, Lundberg E, Navani S, Szigyarto CA-K, Odeberg J, Djureinovic D, Takanen JO, Hober S, et al.: **Proteomics. Tissue-based map of the human proteome.** *Science* 2015, **347**:1260419.

2. Human Protein Atlas proteinatlas.org

3. Giacomelli MG, Husvogt L, Vardeh H, Faulkner-Jones BE, Hornegger J, Connolly JL, Fujimoto JG: **Virtual Hematoxylin and Eosin Transillumination Microscopy Using Epi-Fluorescence Imaging**. *PLoS One* 2016; 11:e0159337

4. Bradski G: **The OpenCV Library**. *Dr Dobb’s Journal of Software Tools* 2000
